# Synthesis and In Vitro Comparison of DOTA, NODAGA and 15-5 Macrocycles as Chelators for the ^64^Cu-Labelling of Immunoconjugates

**DOI:** 10.3390/molecules28010075

**Published:** 2022-12-22

**Authors:** Aurélie Maisonial-Besset, Tiffany Witkowski, Mercedes Quintana, Sophie Besse, Vincent Gaumet, Axel Cordonnier, Cyrille Alliot, Aurélien Vidal, Caroline Denevault-Sabourin, Sébastien Tarrit, Sophie Levesque, Elisabeth Miot-Noirault, Jean-Michel Chezal

**Affiliations:** 1 Université Clermont Auvergne, Inserm, Imagerie Moléculaire et Stratégies Théranostiques, UMR 1240, F-63000 Clermont-Ferrand, France; 2 GIP Arronax, F-44800 Saint-Herblain, France; 3 GICC EA7501, Team IMT, Université de Tours, UFR de Médecine, Bâtiment Vialle, 10 Boulevard Tonnellé, BP 3223, CEDEX 01, 37032 Tours, France; 4 Department of Nuclear Medicine, Jean Perrin Comprehensive Cancer Centre, F-63011 Clermont-Ferrand, France

**Keywords:** copper-64, chelating macrocycles, NODAGA, DOTA, 15-5, trastuzumab

## Abstract

The development of ^64^Cu-based immuno-PET radiotracers requires the use of copper-specific bifunctional chelators (BFCs) that contain functional groups allowing both convenient bioconjugation and stable copper complexes to limit in vivo bioreduction, transmetallation and/or transchelation. The excellent in vivo kinetic inertness of the pentaazamacrocyclic [^64^Cu]Cu-15-5 complex prompted us to investigate its potential for the ^64^Cu-labelling of monoclonal antibodies (mAbs), compared with the well-known NODAGA and DOTA chelators. To this end, three NODAGA, DOTA and 15-5-derived BFCs, containing a pendant azadibenzocyclooctyne moiety, were synthesised and a robust methodology was determined to form covalent bonds between them and azide-functionalised trastuzumab, an anti-HER2 mAb, using strain-promoted azide-alkyne cycloaddition. Unlike the DOTA derivative, the NODAGA- and 15-5-mAb conjugates were radiolabelled with ^64^Cu, obtaining excellent radiochemical yields, under mild conditions. Although all the radioimmunoconjugates showed excellent stability in PBS or mouse serum, [^64^Cu]Cu-15-5- and [^64^Cu]Cu-NODAGA-trastuzumab presented higher resistance to transchelation when challenged by EDTA. Finally, the immunoreactive fraction of the radioimmunoconjugates (88–94%) was determined in HER-2 positive BT474 human breast cancer cells, confirming that the bioconjugation and radiolabelling processes implemented had no significant impact on antigen recognition.

## 1. Introduction

In addition to conventional cancer treatments (i.e., chemotherapy and radiotherapy), a range of targeted therapies has emerged for the treatment of distinct molecular subgroups of patients based on their tumour genotype profile. This field of precision medicine requires predictive tools to enable the accurate identification of potentially responsive patient subpopulations. Positron emission tomography (PET), a non-invasive, functional molecular imaging technique for the whole body, offers the unique advantage of evaluating the target status of both the primary tumour site and distant metastatic lesions. Among the broad range of targeting ligands that can be used as carriers for PET radionuclides, monoclonal antibodies (mAbs) and their derived fragments (e.g., diabodies, affibodies, minibodies, scFvs, etc.) present the advantages of excellent affinity and specificity, most often associated with high and long-lasting accumulation at the target site. However, the pharmacokinetics of immuno-PET agents are highly dependent on their physicochemical (e.g., molecular weight, size, charge, lipophilicity) and biological (e.g., immune interaction, route of clearance, metabolism) properties. Moreover, the bioconjugation strategies employed to graft radionuclides (direct or indirect, random or site-specific) can induce significant structural modifications on the targeting scaffold and thus, have a strong influence on their biodistribution profiles [1,2]. The radiotracers derived from full-length mAbs and, to a lesser extent, Fab-based fragments, spend a long time in the bloodstream, and it can take several hours or even days post injection (p.i.) to achieve a sufficient contrast between target and non-target tissues. β^+^-emitting radionuclides with a suitable half-life are, therefore, essential, such as iodine-124 (t_1/2_ = 4.2 d, E_β+max_ = 1532 keV (11%), 2135 keV (11%)), yttrium-86 (t_1/2_ = 14.7 h, E_β+max_ = 3141 keV (33%)), zirconium-89 (t_1/2_ = 3.27 d, E_β+max_ = 897 keV (23%)) or copper-64 (^64^Cu, t_1/2_ = 12.7 h, E _β+max_ = 655 keV (17%)). The last option is of particular interest due to its low maximum positron energy, which results in a short positron linear range in tissues, leading to high spatial resolution. This radiometal can be produced from biomedical cyclotrons as copper(II) chloride solution, in large quantities and with high molar activity for well-known coordination chemistry processes [3,4,5]. Moreover, this radionuclide can be used in theranostic strategies by combining PET and targeted radionuclide therapy alone, owing to its dual decay profile (E_β+max_ = 655 keV (17%)/E_β −_ max = 573 keV (39%)) [6], or preferably by using the radioisotope pair copper-64 (PET imaging)/copper-67 (radiotherapy, t_1/2_ = 2.58 d, E_β−max_ = 562 keV) [7]. 

**Figure 1:**
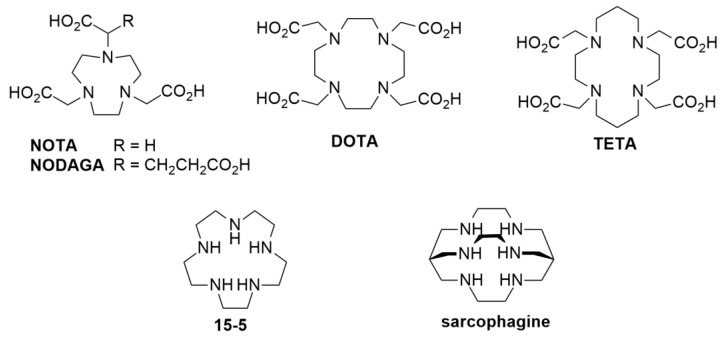
Structures of some macrocyclic chelators used for ^64^Cu-chelation.

The development of ^64^Cu-based immuno-PET radiotracers requires copper-specific chelators, modified with functional groups allowing bioconjugation and commonly known as bifunctional chelators (BFCs). The stability and kinetic inertness of copper-chelator complexes are of the utmost importance to limit in vivo Cu(II)/Cu(I) bioreduction, transmetallation and/or transchelation with human copper-binding proteins. The radiochemical conditions required for efficient copper chelation, particularly temperature, represent another limiting factor, as they may not be compatible with sensitive biomolecules such as mAbs or derived fragments. Much work has been done to circumvent these drawbacks to allow us to benefit from the macrocyclic effect [8], and considerable progress has been made or is in the making by modifying polyazamacrocycles from the 1,4,7-triazacyclononane (TACN), cyclen, cyclam or sarcophagine family (Figure 1) with various cross-bridges and/or pendant arms (e.g., acetate, methylphosphonate, picolinate) [3,4,5].

Surprisingly, despite preclinical evidence of the limited in vitro and in vivo stability of its copper complex [9,10], 2,2′,2″,2‴-(1,4,7,10-tetraazacyclododecane-1,4,7,10-tetrayl)tetraacetic acid (DOTA) is still widely used in ^64^Cu-based immuno-PET clinical trials [11,12].

**Figure 2:**
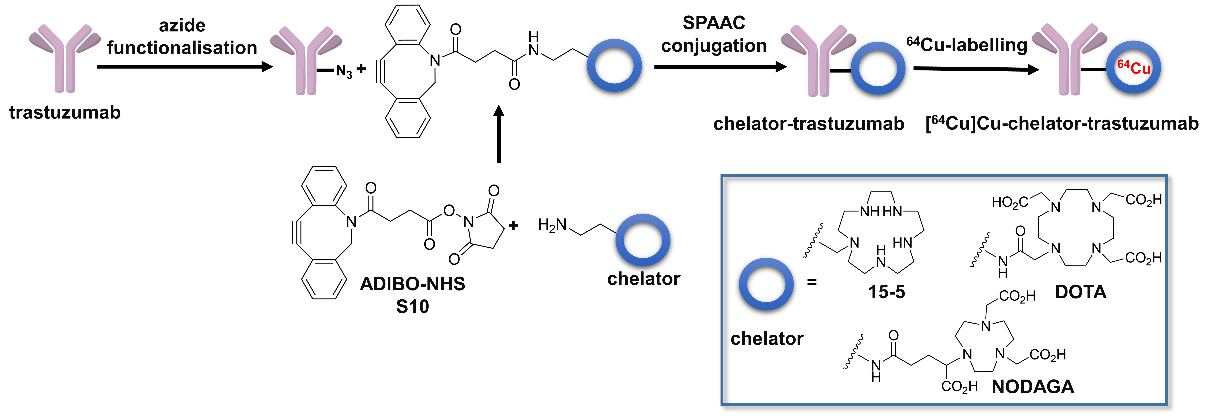
Schematic overview illustrating the strategy used for the design of ^64^Cu-labelled 15-5-, DOTA- and NODAGA-trastuzumab.

We recently developed a series of polyazamacrocyclic chelators conjugated to quaternary ammonium (QA) for ^64^Cu-based PET imaging of the cartilage function and associated diseases (osteoarthritis, arthritis, chondrosarcoma) [13]. Among all the chelators investigated, the ^64^Cu-complex of 15-5 pentaazamacrocycle *N*,*N*,*N*-triethyl-3-(1,4,7,10,13-pentaazacyclopentadecan-1-yl)propan-1-aminium chloride ([^64^Cu]Cu-15-5-QA) showed the highest target-to-non-target tissue ratio in swarm rat chondrosarcoma models, with negligible hepatic accumulation at 1 h p.i., compared with ^64^Cu-labelled cyclam and cyclen derivatives. Similarly, PET imaging and γ-counting-based ex vivo analyses in a healthy rabbit model demonstrated a high uptake of radioactivity in the cartilage, with values of 8.46 and 4.81 injected dose/gram (%ID/g) at 15 min and 1 h p.i., respectively, associated with a rapid hepatic clearance rate of below 0.05%ID/g in the liver as early as 1 h p.i.. Since the liver is the main organ involved in copper metabolism and storage [14,15], hepatic radioactivity uptake is an important indicator of the stability of the radiolabelled copper complex in vivo. It partly reflects the release of uncoordinated ^64^Cu^2+^ from the complex or its transchelation by copper-binding proteins such as the superoxide dismutase enzyme (SOD). Our findings, therefore, demonstrate the excellent in vivo kinetic inertness of the [^64^Cu]Cu-15-5 complex. Moreover, the latter was obtained with a high radiochemical yield (RCY, >99%) and purity (RCP, >99%) under mild radiolabelling conditions (25 °C, 0.1 M sodium citrate buffer) and with a short reaction time (15 min). These results prompted us to continue to explore the use of the 15-5 macrocycle for the ^64^Cu-labelling of biomolecules, as it may encourage radiochemical conversion (RCC) and enhance the in vitro and in vivo stability of the resulting radioimmunoconjugates. To test this hypothesis, a series of three BFCs were selected based on the following ^64^Cu-chelators: 15-5 and the commonly used NODAGA and DOTA azamacrocycles, used as references (Figure 2).

For optimal comparison, we chose strain-promoted azide-alkyne cycloaddition (SPAAC) to ensure covalent linking of the BFCs to the biomolecule [16]. This strategy was intended to produce, from a single batch of azido bioconjugate, the same number of BFCs attached to the targeting macromolecule irrespective of the nature of the chelators used, thus limiting immunoreactivity variation. The BFCs were then conjugated to azido-functionalised anti-HER2 mAb (i.e., trastuzumab), used as a biomolecule model, and the ^64^Cu-labelling kinetics of each chelator-trastuzumab conjugate were evaluated. To compare the influence of chelator moieties on the physicochemical and biological properties of the radioimmunoconjugates, the in vitro stability and HER2-binding affinity of the corresponding ^64^Cu-complexes were also investigated.

## 2. Results and Discussion

As well as the structural differences of each chelator that will affect the physicochemical properties of the resulting immunoconjugates (steric hindrance, net charge, lipophilicity), the bioconjugation strategy can also have a considerable incidence on the in vivo behaviour and antigen-binding ability of the functionalised mAb. Numerous comparison studies have shown that the direct conjugation of various BFCs to the same mAb can result in significant differences in terms of the number of chelators grafted, in spite of efforts to maintain constant reaction conditions [10,16,17,18]. To address this issue, we implemented a two-step strategy involving the pre-functionalisation of trastuzumab onto lysine residues by a common azide-NHS ester coupling before grafting 15-5-, DOTA- and NOTAGA-ADIBO derivatives by SPAAC conjugation (Figure 2). On this basis, a similar number of chelators *per* mAb from the same batch of azide-functionalised trastuzumab, irrespective of the nature of the chelating agent, was achievable.

### 2.1. Organic synthesis

**Scheme 1:**
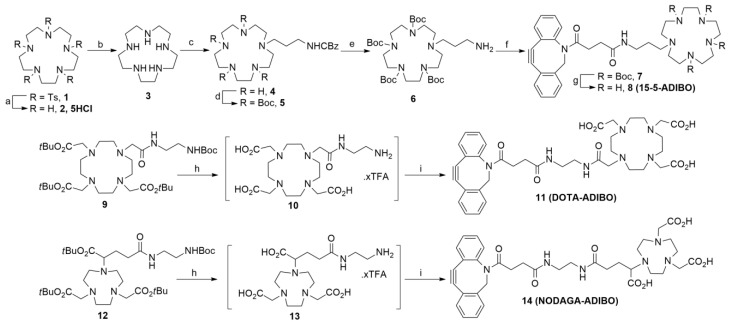
Synthesis of 15-5 precursor **6** and 15-5-, DOTA- and NODAGA-ADIBO conjugates **8**, **11** and **14**: (a) (i) conc. H_2_SO_4_, 110 °C, 48 h; (ii) conc. aq. HCl, RT, 15 h; (b) aq. NaOH, toluene, Dean-Stark, overnight; (c) benzyl 3-bromopropylcarbamate (**S11**), Et_3_N, CHCl_3_, RT, 34 h; (d) Boc_2_O, Et_3_N, CHCl_3_, RT, 18.5 h then 50 °C, 1 h; (e) H_2_, Pd/C 10%, *i*PrOH, RT, 48 h; (f) ADIBO-NHS **S10**, DIPEA, CH_3_CN, RT, 30 h; (g) TFA, anisole, CH_2_Cl_2_, 0 °C, 2 h then RT, 1 h; (h) TFA, anisole, −10 °C then RT, 20–23 h; (i) ADIBO-NHS **S10**, DIPEA, DMSO, RT, 2.5–3 h.

To synthesise the 15-5-, DOTA- and NODAGA-ADIBO conjugates **8**, **11** and **14** we opted for a convergent synthetic approach involving the ADIBO active ester **S10** (see Supplementary Materials) and the corresponding primary amine-functionalised chelators **6**, **10** and **13** as key intermediates (Figure 2, Scheme 1).

Briefly, the free base form of the 15-5 macrocycle **3** was prepared by acidic hydrolysis of the pentatosylate derivative **1** [19] followed by the neutralisation of the resulting hydrochloride salt **2** with an aqueous sodium hydroxide solution and azeotropic drying with toluene (Dean-Stark procedure). The introduction of a primary amine-functionalised pendant arm by monoalkylation of the pentaazamacrocyle **3** with benzyl (3-bromopropyl)carbamate (**S11**) was then investigated. To reduce the generation of polyalkylated side-products without using an excessive amount of **3**, the reaction conditions described by Massue et al. [20] for the *N*-alkylation of cyclen were optimised, affording carbamate **4** with a 57% yield. The secondary amine functions had to be protected before deprotection of the primary amine group and amide coupling with ADIBO active ester **S10**. We naturally turned to the *tert*-butoxycarbonyl (Boc) *N*-protecting group, orthogonal to the hydrogenolysis conditions commonly used for benzyloxycarbonyl (Cbz) removal. Compound **4** was, therefore, converted in tetra-*N*-Boc intermediate **5** in the presence of an excess of (Boc)_2_O and subsequently hydrogenated to afford amine **6** with an excellent overall yield (89%). The latter was then treated with ADIBO-NHS **10** to produce *N*-Boc fully protected 15-5-ADIBO conjugate **7**. Final deprotection of the *N*-Boc groups using a mixture of trifluoroacetic acid (TFA)/dichloromethane solution and anisole (used as a scavenger), followed by preparative RP-HPLC purification and freeze-drying, afforded the free base 15-5-ADIBO conjugate **8** with a 71% yield. It is worth mentioning the high reactivity and instability of the cyclooctynes (e.g., bicyclo [6.1.0]non-4-yne (BCN) or ADIBO group) under strongly acidic conditions, which usually lead to rapid decomposition [21,22]. This meant that the TFA concentration, temperature and reaction time had to be carefully controlled to minimize product degradation. Considering that the rate of acid-catalysed deprotection of a *tert*-butyl ester is lower than that of the Boc-protected amine. DOTA and NODAGA precursors **9** and **12** (see Supplementary Materials) were fully deprotected in amines **10** and **13**, respectively, before the reaction with the ADIBO active ester **S10**. DOTA- and NODAGA-ADIBO conjugates **11** and **14** were thus obtained with 48 and 67% yields, respectively, after RP-HPLC purification and freeze–drying. Each chelator-ADIBO sample can be stored, protected from light and moisture, at ambient temperature for 3 months without significant degradation.

### 2.2. Immunoconjugation

**Figure 3:**
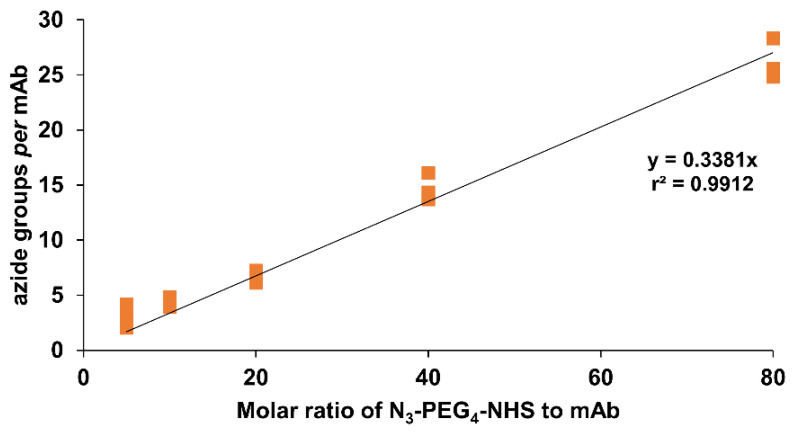
Average number of azide groups *per* trastuzumab calculated by MALDI-TOF MS analysis as a function of the initial molar ratio of N_3_-PEG_4_-NHS:mAb (n = 3–4 independent experiments). Each reaction was performed at room temperature in phosphate buffer (pH 9.2) for 15 min before SEC-HPLC purification. Pearson correlation coefficient r^2^ and equation of linear regression are shown.

With the chelator-ADIBO intermediates **8**, **11** and **14** available, we explored the amide coupling of N_3_-PEG_4_-NHS **S32** to lysine residues of trastuzumab, chosen as our model mAb. The first experiments investigated the influence of pH, reaction time and temperature for a fixed 40:1 molar ratio of **S32**:trastuzumab. After SEC-HPLC purification and MALDI-TOF-MS analysis, it became apparent that acylation was accelerated under alkaline conditions, producing the same number of azide residues *per* mAb (13.8 ± 0.1, n = 3) in 15 min at pH 9.2 as overnight at pH 7.4. This is consistent with the literature, which indicates that increasing pH facilitates primary amine reactivity but at the cost of enhanced hydrolysis of the NHS ester [23]. With a short and efficient conjugation method, larger amounts of azide active ester **S32** (i.e., 5-, 10-, 20-, 40- and 80-fold molar excess compared with trastuzumab) were assessed. Figure 3 shows the good linear relationship observed between the number of grafted azide groups *per* mAb, determined after SEC-HPLC purification and MALDI-TOF-MS analysis, and the initial molar ratio of **S32** to trastuzumab (r^2^ = 0.991).

Increasing the number of metal-binding sites *per* mAb offers the advantage of enhancing the molar activity of the radiotracer but can also have a dramatic impact on the antigen-binding affinity and the in vivo pharmacokinetics of the immunoconjugate. To optimise the preservation of the functional properties of the mAb, the trastuzumab was conjugated with azide **S32** at a molar ratio of 1:10. In these conditions, the average number of azide functions grafted to a single mAb was 4.18 ± 0.32. Having achieved reproducible access to N_3_-functionalised trastuzumab, we focused our efforts on the subsequent coupling step with the ADIBO-chelator derivatives. The SPAAC conjugation was carried out overnight at 4 °C in PBS, with a 50-time molar excess of 15-5-, DOTA- or NODAGA-ADIBO conjugate **8**, **11** or **14**, respectively, compared with N_3_-trastuzumab. Any remaining unreacted chelator-ADIBO was then removed by ultrafiltration and SEC-HPLC. These conditions produced an average number of chelators *per* trastuzumab of 3.74 ± 0.18, 3.64 ± 0.16 and 3.89 ± 0.22 for the 15-5-, DOTA- and NODAGA-trastuzumab immunoconjugates, respectively (Table 1), determined by MALDI-TOF-MS in three independent experiments performed in triplicate.

Overall, the narrow range of values obtained, slightly lower but close to those found for the parent pre-functionalised N_3_-trastuzumab, confirmed that the two-step functionalisation strategy implemented was highly efficient and reproducible, regardless of the structure of the chelator.

### 2.3. ^64^Cu-Labelling of Chelator-Trastuzumab Conjugates

**Figure 4:**
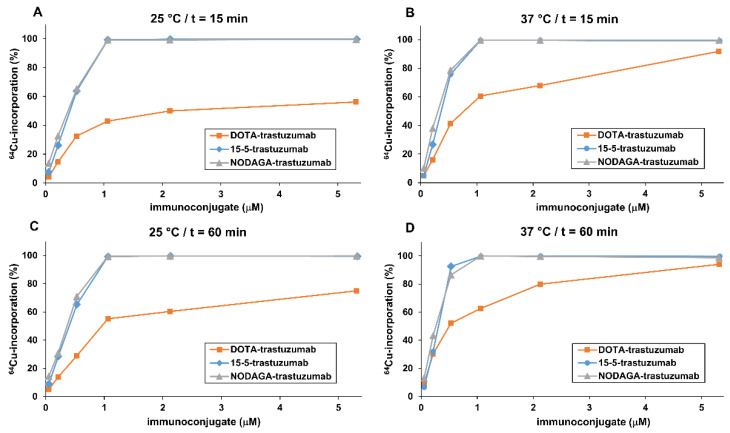
Percentage of ^64^Cu-incorporation for the radiolabelling of 15-5-trastuzumab (blue line, diamond dots), DOTA-trastuzumab (orange line, square dots) and NODAGA-trastuzumab (grey line, triangle dots) as a function of time (15 min: **A**,**B**; 60 min: **C**,**D**), temperature (25 °C: **A**,**C**; 37 °C: **B**,**D**) and concentration of immunoconjugates (n = 3). For all experiments, the activity of [^64^Cu]CuCl_2_ (3.4 MBq) and the final volumes were held constant.

The radiolabelling reaction conditions of the 15-5-, DOTA- and NODAGA-trastuzumab immunoconjugates with copper-64 chloride ([^64^Cu]CuCl_2_) were then investigated. The concentration of chelator-trastuzumab, reaction time and temperature were monitored while maintaining constant the pH (ca. 5.4–5.5), volume and activity of [^64^Cu]CuCl_2_ (3.4 MBq) (Figure 4).

RCC was monitored by radio-instant thin-layer chromatography (radio-ITLC) eluted with 0.1 M citrate buffer (pH 4.5). Initial conditions were maintained at 25 °C for 15 min with increasing concentrations of immunoconjugates from 53 nM to 5.3 µM, corresponding to 0.25 μg to 25 μg of mAb *per* sample (Figure 4A). While DOTA-trastuzumab showed limited complexation of copper-64, even at the maximal concentration of immunoconjugates (i.e., 56% at 5.3 µM), full ^64^Cu-incorporation was achieved from 1.1 µM of 15-5- and NODAGA-trastuzumab. Increasing the reaction time (from 15 to 60 min, Figure 4B), the reaction temperature (from 25 to 37 °C, Figure 4C) or both (Figure 4D) had a positive impact on the radiolabelling rate of DOTA-trastuzumab for all the concentrations tested. The best result was obtained for the largest concentration of immunoconjugate at 37 °C for 60 min (i.e., 94% at 5.3 µM). These results are consistent with previous studies showing that ^64^Cu-labelling of DOTA-immunoconjugates is generally carried out at temperatures as high as 40–43 °C with prolonged reaction times [10,12,24]. Both temperature and reaction time also proved to have a slight influence on the ^64^Cu-complexation of 15-5- and NODAGA-trastuzumab. This was particularly noticeable when using 0.53 µM immunoconjugates with RCC of 92 and 87% at 37 °C for 60 min vs. 64 and 65% at 25 °C for 15 min for 15-5- and NODAGA-functionalised trastuzumab, respectively. At this stage, when comparing the three immunoconjugates harbouring the same average number of chelators *per* mAb, it was clear that the 15-5 and NODAGA macrocycles were significantly superior to DOTA in terms of RCC.

**Figure 5:**
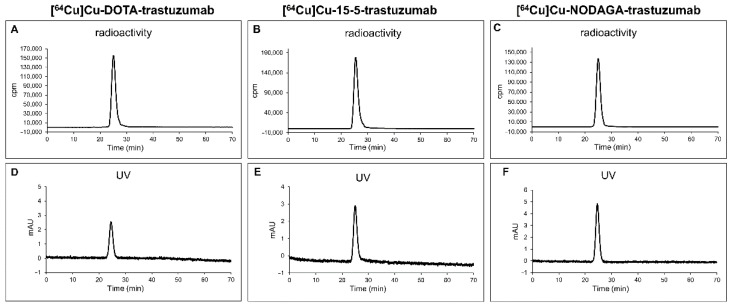
Analytical radio-HPLC chromatograms of [^64^Cu]Cu-DOTA-trastuzumab (obtained post-purification) (**A**), [^64^Cu]Cu-15-5-trastuzumab (**B**) and [^64^Cu]Cu-NODAGA-trastuzumab (**C**), compared to their analytical UV-HPLC chromatograms (**D**–**F**), respectively. The radio-HPLC detector was connected in series after the UV detector accounting for the slight difference in retention times (~0.5 min) observed between UV and radioactive signals.

For the next steps, the radiolabelling conditions were adapted to give the best labelling yield and purity for each conjugate, while maintaining constant molar activity. The least efficient chelating agent, i.e., DOTA, was, therefore, used as the basis to set the initial activity of the ^64^Cu:chelator-mAb ratio at ~20 GBq/µmol. At the end of the reaction, the radiolabelling mixture was incubated with a 7.5-fold molar excess of EDTA for 5 min to remove all traces of unbound ^64^Cu^2+^. For [^64^Cu]Cu-DOTA-trastuzumab, radio-ITLC monitoring indicated that the initial RCC of 94% obtained after heating the radiolabelling mixture to 37 °C for 60 min was significantly decreased by 10 to 30% after the EDTA competition step. The transchelation observed with EDTA could not be attributed entirely to the non-specific binding of ^64^Cu^2+^ to native mAb, estimated at 3% after trastuzumab incubation at 37 °C for 30 min with [^64^Cu]CuCl_2_ in sodium acetate buffer (0. 2 M, pH 5.5) and radio-ITLC analysis. One explanation could be the formation of weak and non-specific ^64^Cu-complexes with both DOTA carboxymethyl pendant arms and amino-acid side chains of the protein as ligands. The radioimmunoconjugate was then purified by SEC on a PD-10 column to afford [^64^Cu]Cu-DOTA-trastuzumab in 53 ± 5% RCY with RCP >98% estimated by either radio-SEC-HPLC or radio-ITLC (Figure 5A and Figure S1).

For [^64^Cu]Cu-15-5-trastuzumab and [^64^Cu]Cu-NODAGA-trastuzumab, no purification step was required as excellent RCC (>99%) were obtained after a reaction time of 15 min at room temperature, without any detection of unbound ^64^Cu^2+^ upon competition with EDTA, as confirmed by radio-ITLC (Figure S1, Supplementary Materials) and radio-SEC-HPLC analyses (Figure 5B,C).

### 2.4. In Vitro Stability of ^64^Cu-Labelled Immunoconjugates

**Figure 6:**
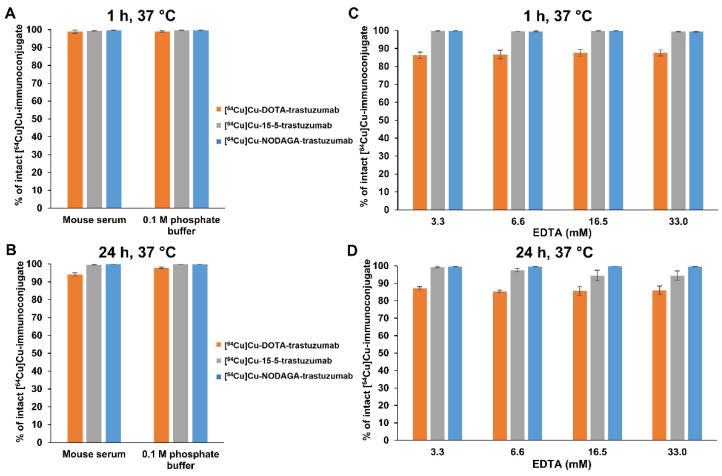
Complex stability of [^64^Cu]Cu-chelator-trastuzumab in 0.1 M phosphate buffer and mouse serum after incubation at 37 °C for 1 h (**A**) and 24 h (**B**). [^64^Cu]Cu-chelator-trastuzumab competition against increasing concentrations of EDTA at 37 °C for 1 h (**C**) and 24 h (**D**). The stability was represented as the percentage of intact ^64^Cu-labelled conjugate as determined by radio-ITLC in three independent experiments (n = 3).

The in vitro stability of the three radioimmunoconjugates was then investigated over 24 h at 37 °C in 0.1 M phosphate buffer, mouse serum and EDTA challenge solutions (Figure 6).

The percentage of copper-64 loss from ^64^Cu-labelled immunoconjugates was assessed by radio-ITLC with 1 mM EDTA in PBS eluent. Both radioimmunoconjugates displayed high stability in 0.1 M phosphate buffer and mouse serum over 1 h, with more than 98% radioimmunoconjugates remaining intact (Figure 6A). After 24 h incubation (Figure 6B), only a slight but significant release of copper-64 was visible for [^64^Cu]Cu-DOTA-trastuzumab, which was more pronounced in the mouse serum than in the phosphate buffer, with 6 and 2% losses, respectively. To assess the effect of transchelation, the radioimmunoconjugates were incubated at 37 °C with increasing concentrations of EDTA ranging from 3.3 to 33 mM (corresponding to 5000 to 50,000 EDTA:mAb molar ratios) (Figure 6C,D). The [^64^Cu]Cu-NODAGA-trastuzumab complex remained highly stable over 24 h, up to 33 mM EDTA solution (>99%), indicating its remarkable kinetic inertness. The same trend was observed for [^64^Cu]Cu-15-5-trastuzumab over 1 h (>99%), with only slight transchelation occurring at the 24 h time point and for the highest concentrations of EDTA studied (i.e., ~5.5% for 16.5 and 33 mM EDTA solution). For [^64^Cu]Cu-DOTA-trastuzumab, significant amounts of copper-64 were rapidly released into the solution and trapped by EDTA after incubation of just 1 h (12–14%), irrespective of the EDTA concentration used. Prolonged incubation time did not significantly improve the transchelation of copper-64.

### 2.5. In Vitro Receptor Binding Assays

As previously stated, chelator conjugation as well as radiolabelling procedures could significantly alter the biological properties of mAbs, particularly their immunoreactivity. The most widely used method for determining the IRF of a radioimmunoconjugate is based on the Lindmo assay [25]. According to this protocol, extrapolating the saturation binding of radiolabelled mAb at infinite antigen excess, IRF can be determined as the inverse of the y-intercept value of the linear regression line obtained by plotting (B+S)/B as a function of 1/[cells], where B and S are the radioactivity counted in pellets and supernatants, respectively. However, the Lindmo method is dependent on experimental conditions and can sometimes lead to unreliable and/or overestimated results [26,27]. According to the recent study by Denoël et al. [28], these limitations can be overcome by applying the rectangular hyperbola method of modern data analysis software (e.g., GraphPad Prism) to fit the binding curve of B/(B+S) as a function of cell concentration that provides the IRF at infinite antigen excess by extrapolation. The IRF of the [^64^Cu]Cu-NODAGA-, [^64^Cu]Cu-15-5- and [^64^Cu]Cu-DOTA-immunoconjugates was, thus, determined on the HER2-positive human breast cancer cell line BT474 and compared with that obtained for trastuzumab radiolabelled with iodine-125 (see Table 1 and Figure S2 in Supplementary Materials). The IRF values of all the ^64^Cu-labelled conjugates were similar (87.9–93.8%) and close to that of native trastuzumab (93.8 ± 0.64%). These results clearly demonstrate that [^64^Cu]Cu-NODAGA-, [^64^Cu]Cu-15-5- and [^64^Cu]Cu-DOTA-trastuzumab bind with high affinity to HER2-positive BT474 cells in vitro.

## 3. Materials and Methods

### 3.1. General

All commercially available reagents and solvents were purchased at the following commercial suppliers: Sigma Aldrich, Alpha Aesar, ABX, Acros Organics, Fisher Scientific or Carlo Erba Reagents. Tetrahydrofuran was dried over a Pure Solv™ Micro Solvent Purification System (Sigma-Aldrich, Saint-Quentin-Fallavier, France) and whenever necessary, other solvents were dried using common techniques [29]. Temperatures indicated in the protocols correspond to the temperature of the oil bath. Analytical thin layer chromatography (TLC) was performed on precoated silica gel 60 F_254_ or neutral aluminium oxide 60 F_254_ plates (Merck, Fontenay Sous Bois, France or Macherey-Nagel, Hoerdt, France) and visualised with UV light (254 nm) and/or developed with phosphomolybdic acid (8 wt%) in ethanol. Flash column chromatography was performed on silica gel 60A normal phase, 35–70 µm (Merck or SDS) or neutral aluminium oxide 90 standardised, 63–200 µm (Merck). Uncorrected melting points (mp) were recorded on an electrothermal capillary Digital Melting Point Apparatus IA9100 (Bibby Scientific). NMR spectra (200.13 or 500.13 MHz for ^1^H and 50.32 or 125.76 MHz for ^13^C) were recorded on Bruker Avance 200 or 500 instruments with chemical shift values (δ) expressed in parts *per* million (ppm) relative to residual solvent as standard and coupling constants (*J*) given in Hz. Infrared spectra (IR) were recorded in the range 4000–440 cm^−1^ on a Nicolet IS10 (Fisher Scientific) with attenuated total reflectance (ATR) accessory. Low molecular weight organic compounds were analysed by High-Resolution Mass Spectrometry (HRMS) in positive or negative mode (Micromass Q-Tof micro Mass Spectrometer, Waters, Guyancourt, France). Preparative RP-HPLC purifications were carried out on a CombiFlash EZ prep system (Teledyne Isco, Lincoln, USA) equipped with a UV-visible detector. Separation was performed on a C18 column (Teledyne, Redisep Prep C18, 20 mm × 250 mm, 100 Å pore size, 5 µm) at room temperature using the following solvent systems: 0.01% TFA in water (solvent A) and 0.01% TFA in acetonitrile (solvent B); 0–3 min: isocratic elution 95% A; 3–43 min: gradient elution 95–0% A. The mobile phase flow rate was maintained at 15 mL.min^−1^ and eluents were monitored at 220 and 254 nm. The following protocol was used to remove trace metal contaminants from deionised water or aqueous buffers: Chelex 100 resin (Bio-Rad Laboratories, Marnes-la-Coquette, France) was added to the appropriate solution (20 g.L^−1^) and the resulting mixture was left for 24 h at room temperature and then filtered through a 0.22 µm PES membrane filter (Corning 1000 mL filtration system). Whenever necessary, removing trace metals from vessels was performed by soaking it in 4 M aqueous nitric acid solution for several hours then draining, washing successively with metal-free water, ethanol and diethyl ether and air-drying at room temperature before use. The humanised anti-HER2 monoclonal antibody, trastuzumab-qyyp, subsequently referred to as trastuzumab, was generously provided by the cancer center Jean Perrin (Clermont-Ferrand, France). The Amicon Ultracel 4 centrifugal filter (50 kDa molecular weight cut-off) was purchased from Merck Millipore. Bioconjugations, radiolabelling and storage of the immunoconjugates were performed in Protein LoBind tubes (Eppendorf, PCR clean, Montesson, France). Centrifugations were carried out at 4000 rpm for 20–30 min at 4 °C on a refrigerated Centrifuge 5804 R (Eppendorf, Montesson, France). Analytical and semi-preparative size exclusion–high-performance liquid chromatography (SEC–HPLC) was performed on a system consisting of a HP1100 (Hewlett Packard, Les Ulis, France) and a Flo-one A_500_ Radiomatic detector (Packard, Canberra, Australia). SEC HPLC separations and analyses were achieved on a Superose 12 column, 10/300 GL, 11 µm (GE Healthcare, Buc, France) using an isocratic mobile phase (metal-free PBS) at a flow rate of 0.5 mL·min^−1^ (λ = 220, 254 and 288 nm). The determination of the mAb and immunoconjugate concentrations was performed on a NanoDrop spectrophotometer at 280 nm (MultiskanGo, Thermoscientific, Illkirch, France). Matrix-assisted laser desorption/ionisation–time of flight–mass spectrometry (MALDI–TOF–MS) analyses were carried out on a MALDI-TOF/TOF Autoflex Speed (Bruker Daltonics, Wissembourg, France). The native and modified mAbs in PBS were first diluted to 2 mg/mL with a mixture of acetonitrile/water (30/70, *v*/*v*) containing 0.1% TFA (solvent), followed by a set of three dilutions (2-, 4-, 16-fold) in matrice solution (10 mg/mL sinapinic acid from Sigma, France diluted with solvent). The three dilution/mAb were spotted (1 µL) on the MALDI-MS target (MTP ANCHORCHIP# 384 BC, Bruker Daltonics, France) and the droplet was left to dry. The reference mAb IgG1 (AB Sciex, Framingham, USA) at 1mg/mL was used as the external calibrant. A volume of 0.4 µL of a calibrant/matrice mixture (50/50 *v*/*v*) was deposited onto the calibrant anchor spot in close proximity to samples (one spot of calibrant *per* four samples spots). A total of 1000 laser shots were averaged for each spectrum, in the mass range between 30 and 210 kDa. Acquisitions were realized in the positive linear ion mode with 19.00 kV ion source voltage, a Smartbean 1 setting, a laser intensity set to 15% and 1000 Hz as laser repetition rate. Copper-64 dichloride in 0.1 M hydrochloric acid (1600–2800 MBq/mL) was obtained from ARRONAX cyclotron (Saint-Herblain, France). Radionuclide purity was determined by gamma spectrometry using a DSPEC-JR-2.0 type 98-24B HPGE detector (Ametek, Berwyn, USA), and chemical purity was measured by ICP-OES using an iCAP 6500 DUO (Thermo Fisher Scientific, Illkirch, France). Radio-ITLC analysis was measured on a miniGITA Dual radio-TLC system (Elysia-Raytest GmbH, Straubenhardt, Germany) using silica gel-impregnated chromatography paper (Varian Inc., Agilent, Santa Clara, USA) eluted with a solution of 0.1 M citrate buffer (pH 4.5) or 1 mM EDTA in PBS. Regardless the elution conditions, the radiolabelled immunoconjugates remained at the origin (R*_f_* = 0) while unbound ^64^Cu^2+^ migrated near the solvent front (R*_f_* = 0.9–1).

### 3.2. Organic Synthesis

#### 3.2.1. 1,4,7,10,13-Pentaazacyclopentadecane, pentahydrochloride Salt (2)

A solution of 1,4,7,10,13-pentatosyl-1,4,7,10,13-pentaazacyclopentadecane (1) [19] (5.23 g, 5.30 mmol) in concentrated sulfuric acid (40 mL) was stirred at 110 °C for 48 h. After cooling to room temperature, the reaction mixture was poured dropwise in a mixture of methanol/diethyl ether (1/1, *v*/*v*, 80 mL) cooled to 0 °C. The resulting solution was stirred at 0 °C for 45 min. The grey precipitate formed was filtered and taken up in methanol (45 mL). A concentrated hydrochloric acid solution (37 wt%, 10 mL) was added and the resulting solution was stirred at room temperature for 20 h. The grey precipitate was filtered, washed successively with ethanol (2 × 5 mL) and diethyl ether (2 × 5 mL) and dried overnight at 35 °C in a vacuum desiccator to give compound 2 as a grey solid (2.10 g, 5.28 mmol) which was used in the next step without further purification. Yield: quant.; mp 258–262 °C; IR (ATR accessory) ν 3220–2850, 2850–2500, 2437, 2390, 1453, 1207, 1172, 1043 cm^−1^; ^1^H NMR (500.13 MHz, D_2_O + 1,4-dioxane) δ 3.57 (s, 20H); ^13^C NMR (125.76 MHz, D_2_O + 1,4-dioxane) δ 44.5 (10C).

#### 3.2.2. 1,4,7,10,13-Pentaazacyclopentadecane (3)

To a solution of sodium hydroxide (1.50 g, 37.5 mmol) in deionised water (10 mL), 1,4,7,10,13-pentaazacyclopentadecane, pentahydrochloride salt (2) (2.48 g, 6.24 mmol) and toluene (60 mL) were added successively. The reaction mixture was refluxed overnight in a Dean-Stark apparatus in order to remove water. Toluene was decanted and the residue was taken up with fresh toluene (20 mL). The resulting solution was heated at reflux for 1.5 h and then filtered hot. The organic layers were combined, dried over sodium sulfate, filtered and evaporated under reduced pressure to give compound 3 (1.11 g, 5.16 mmol) as a white solid. Yield: 83%; mp 85–87 °C (Lit.: 100–102 °C [30]); IR (ATR accessory) ν 3700–3100, 3100–2500, 1555, 1445, 1408, 1354, 1274, 1126, 1058 cm^−1^; ^1^H NMR (200.13 MHz, CDCl_3_) δ 1.93 (brs, 5H), 2.65 (s, 20H); ^13^C NMR (125.76 MHz, CDCl_3_) δ 48.7 (10C).

#### 3.2.3. Benzyl (3-(1,4,7,10,13-Pentaazacyclopentadecan-1-yl)propyl)carbamate (4)

To a solution of 1,4,7,10,13-pentaazacyclopentadecane (**3**) (1.08 g, 5.03 mmol) and triethylamine (707 μL, 5.03 mmol) in anhydrous chloroform (60 mL), a solution of benzyl (3-bromopropyl)carbamate (**S11**) (685 mg, 2.52 mmol) (see Supplementary Materials) in anhydrous chloroform (140 mL) was slowly added dropwise, over 3 h and under argon. The reaction mixture was heated at 50 °C for 34 h. After cooling to room temperature, the solvent was removed under reduced pressure and the residue was purified by column chromatography (Al_2_O_3_, dichloromethane/ethanol/ammonia, 95/5/0.2, *v*/*v*/*v*) to give compound **4** as a colourless oil (580 mg, 1.43 mmol). Yield: 57%; R*_f_* = 0.08 (Al_2_O_3_, dichloromethane/ethanol/ammonia, 85/15/0.2, *v*/*v*/*v*); IR (ATR accessory) ν 3500-3100, 2934, 2817, 1703, 1533, 1453, 1254, 1129, 1061, 1026 cm^−1^; ^1^H NMR (200.13 MHz, CDCl_3_) δ 1.67 (m, 2H), 2.42 (t, 2H, *J* = 6.2 Hz), 2.40-2.90 (m, 24H), 3.25 (m, 2H), 5.06 (s, 2H), 6.31 (m, 1H), 7.32 (m, 5H); ^13^C NMR (50.32 MHz, CDCl_3_) δ 26.8, 38.7, 46.9 (2C), 47.6 (2C), 47.9 (2C), 48.9 (2C), 51.1, 53.9 (2C), 66.2, 127.8, 127.9 (2C), 128.3 (2C), 136.8, 156.7; HRMS m/z calculated for C_21_H_39_N_6_O_2_^+^ [M + H]^+^: 407.3129, found: 407.3128.

#### 3.2.4. Tetra-tert-butyl 13-(3-(((benzyloxy)carbonyl)amino)propyl)-1,4,7,10,13-pentaazacyclopentadecane-1,4,7,10-tetracarboxylate (5)

To a solution of compound **4** (205 mg, 0.504 mmol) in anhydrous chloroform (15 mL), triethylamine (354 μL, 2.52 mmol) and di-*tert*-butyl dicarbonate (550 mg, 2.52 mmol) were successively added, under argon. The reaction mixture was stirred at room temperature for 18.5 h and then warmed to 50 °C for 1 h. After cooling to room temperature, the solvent was removed under reduced pressure and the residue was purified by column chromatography (Al_2_O_3_, dichloromethane/ethanol, 99/1, *v*/*v*) to give compound **5** as a colourless oil which solidified on standing (368 mg, 0.456 mmol). Yield: 90%; mp 62–64 °C; R*_f_* = 0.47 (Al_2_O_3_, dichloromethane/ethanol, 99/1, *v*/*v*); IR (ATR accessory) ν 2974, 2930, 1689, 1464, 1412, 1365, 1242, 1156 cm^−1^; ^1^H NMR (200.13 MHz, CDCl_3_) δ 1.42 (m, 36H), 1.58 (m, 2H), 2.40–2.65 (m, 6H), 3.15–3.40 (m, 18H), 5.04 (s, 2H), 5.81 (m, 1H), 7.29 (s, 5H); ^13^C NMR (50.32 MHz, CDCl_3_) δ 26.9, 28.3 (12C), 39.4, 46.0–47.5 (m, 8C), 52.5–53.5 (m, 3C), 66.1, 79.8 (4C), 127.8 (3C), 128.3 (2C), 136.8, 155.0 (4C), 156.3; HRMS *m/z* calculated for C_41_H_71_N_6_O_10_^+^ [M + H]^+^: 807.5226, found: 807.5236.

#### 3.2.5. Tetra-tert-butyl 13-(3-aminopropyl)-1,4,7,10,13-pentaazacyclopentadecane-1,4,7,10-tetracarboxylate (6)

To a degassed solution of carbamate 5 (620 mg, 0.77 mmol) in propan-2-ol (40 mL), Pd/C 10% (182 mg) was added. After stirring at room temperature for 48 h under a hydrogen atmosphere, the suspension was filtered over 0.45 μm PTFE membrane filter and the filtrate was evaporated under reduced pressure to give compound 6 as a white solid (510 mg, 0.76 mmol). Yield: 99%; mp 95–98 °C; IR (ATR accessory) ν 3000–2750, 1689, 1467, 1414, 1391, 1365, 1245, 1157 cm^−1^; ^1^H NMR (200.13 MHz, CDCl_3_) δ 1.41 (m, 36H), 1.60 (m, 2H), 2.40–2.65 (m, 6H), 2.77 (t, 2H, *J* = 6.1 Hz), 3.10–3.50 (m, 16H), 4.50 (brs, 2H); ^13^C NMR (50.32 MHz, CDCl_3_) δ 28.4 (13C), 39.6, 46.4–47.0 (m, 8C), 52.5–53.5 (m, 3C), 80.0 (4C), 155.2 (4C); HRMS *m/z* calculated for C_33_H_65_N_6_O_8_^+^ [M + H]^+^: 673.4858, found: 673.4846.

#### 3.2.6. 4-(11,12-Didehydrodibenzo[b,f]azocin-5(6H)-yl)-4-oxo-N-(3-(4,7,10,13-tetrakis(2-(tert-butoxy)-2-oxoethyl)-1,4,7,10,13-pentaazacyclopentadecan-1-yl)propyl)butanamide (7)

To a solution of tetra-*tert*-butyl 13-(3-aminopropyl)-1,4,7,10,13-pentaazacyclopentadecane-1,4,7,10-tetracarboxylate (**6**) (184 mg, 273 μmol) in anhydrous acetonitrile (5 mL), *N*,*N*-diisopropylethylamine (47 μL, 276 μmol) and ADIBO active ester **S10** (see Supplementary Materials) (110 mg, 273 μmol) were successively added, under argon. After stirring at room temperature for 30 h, the solvent was removed under reduced pressure and the residue was purified by column chromatography (Al_2_O_3_, dichloromethane/ethanol 99/1, *v*/*v*) to give compound **7** as a white solid (232 mg, 242 μmol). Yield: 88%; mp 88–90 °C; R*_f_* = 0.26 (Al_2_O_3_, dichloromethane/ethanol, 99/1, *v*/*v*); IR (ATR accessory) ν 2973, 1690, 1413, 1392, 1365, 1244, 1158 cm^−1^; ^1^H NMR (500.13 MHz, CDCl_3_) δ 1.37 (s, 18H), 1.40 (s, 18H), 1.44 (m, 2H), 1.87 (td, 1H, *J* = 6.4, 16.9 Hz), 2.10 (m, 1H), 2.36 (m, 3H), 2.45-2.65 (m, 4H), 2.73 (td, 1H, *J* = 6.8, 16.9 Hz), 3.07 (m, 2H), 3.10–3.45 (m, 16H), 3.58 (d, 1H, *J* = 13.9 Hz), 5.08 (d, 1H, *J* = 13.9 Hz), 6.44 (m, 1H), 7.16 (dd, 1H, *J* = 1.1, 7.5 Hz), 7.22 (td, 1H, *J* = 1.0, 7.6 Hz), 7.25–7.40 (m, 4H), 7.45 (m, 1H), 7.60 (d, 1H, *J* = 7.5 Hz); ^13^C NMR (50.32 MHz, CDCl_3_) δ 26.9, 28.4 (12C), 30.2, 31.2, 37.2, 46.7 (m, 8C), 53.4 (m, 3C), 55.4, 79.9 (4C), 107.9, 114.6, 122.3, 123.1, 125.4, 126.9, 127.6, 128.0 (2C), 128.6, 129.3, 132.2, 148.1, 151.4, 155.1 (brs, 4C), 172.0, 172.2; HRMS *m*/*z* calculated for C_52_H_78_N_7_O_10_^+^ [M + H]^+^: 960.5805, found: 960.5811.

#### 3.2.7. 4-(11,12-Didehydrodibenzo[b,f]azocin-5(6H)-yl)-4-oxo-N-(3-(1,4,7,10,13-pentaazacyclopentadecan-1-yl)propyl)butanamide (8 or 15-5-ADIBO)

To a solution of compound 7 (255 mg, 266 µmol) in anhydrous dichloromethane (1.5 mL), anisole (100 µL) and trifluoroacetic acid (0.75 mL) were successively added at 0 °C and under argon. After stirring at 0 °C for 2 h, the reaction mixture was warmed to room temperature and stirred for 1 h. The volatiles were removed under reduced pressure. The residue was purified by preparative RP-HPLC (Combiflash system) (*t*_R_ = 23 min) and the combined fractions were basified to pH 8 using a concentrated ammonia solution (28–30 wt. %) to give, after freeze–drying, compound 8 (15-5-ADIBO) as a white fluffy solid (106 mg, 189 µmol). Yield: 71%; IR (ATR accessory) ν 3292, 3100-2500, 1671, 1564, 1482, 1471, 1445, 1430, 1422, 1198, 1176, 1121 cm^−1^; ^1^H NMR (500.13 MHz, CD_3_OD) δ 1.55 (quint., 2H, *J* = 6.8 Hz), 1.97 (td, 1H, *J* = 6.3, 16.5 Hz), 2.14 (m, 1H), 2.32 (m, 1H), 2.39 (t, 2H, *J* = 6.7 Hz), 2.53 (m, 4H), 2.57 (m, 4H), 2.63 (m, 8H), 2.71 (m, 5H), 3.12 (m, 2H), 3.70 (d, 1H, *J* = 14.0 Hz), 5.12 (d, 1H, *J* = 14.0 Hz), 7.25 (dd, 1H, *J* = 1.2, 7.3 Hz), 7.32 (td, 1H, *J* = 1.3, 7.5 Hz), 7.36 (td, 1H, *J* = 1.4, 7.5 Hz), 7.42–7.50 (m, 3H), 7.60 (m, 1H), 7.65 (d, 1H, *J* = 7.5 Hz); ^13^C NMR (50.3 MHz, CD_3_OD) δ 27.9, 31.6, 32.0, 38.7, 48.1 (2C), 48.6 (2C), 48.8 (2C), 49.6 (2C), 53.0, 55.6 (2C), 56.7, 108.8, 115.6, 123.7, 124.4, 126.4, 128.1, 128.9, 129.2, 129.6, 130.0, 130.6, 133.5, 149.5, 152.7, 174.0, 174.3; HRMS *m*/*z* calculated for C_32_H_46_N_7_O_2_^+^ [M + H]^+^: 560.3708, found: 560.3696.

#### 3.2.8. 2,2′,2″-(10-(2-oxo-2-((2-(4-(11,12-didehydrodibenzo[b,f]azocin-5(6H)-yl)-4-oxobutanamido)ethyl)amino)ethyl)-1,4,7,10-tetraazacyclododecane-1,4,7-triyl)triacetic acid, triammonium salt (11 or DOTA-ADIBO)

To a solution of DOTA derivative 9 (see Supplementary Materials) (572 mg, 0.80 mmol) in anisole (540 µL), trifluoroacetic acid (8.5 mL) was added at −10 °C. The reaction mixture was warmed to room temperature and stirred for 23 h. The solution was evaporated under reduced pressure and the remaining trifluoroacetic acid was removed by co-evaporation with anhydrous dichloromethane (2 × 7 mL). The residue was triturated with anhydrous diethyl ether (2 × 8 mL) and dried under reduced pressure to give the corresponding fully unprotected derivative 10 as a hygroscopic ochre solid (HRMS *m/z* calculated for C_18_H_35_N_6_O_7_ [M + H]^+^: 447.2562, found: 447.2562). To a solution of the latter in anhydrous dimethyl sulfoxide (1.5 mL) were successively added under argon *N*,*N*-diisopropylethylamine (680 µL, 4.00 mmol) and after 5 min ADIBO active ester S10 (see Supplementary Materials) (300 mg, 0.93 mmol). The reaction mixture was stirred at room temperature for 3 h. The resulting solution was neutralised to pH 6 using trifluoroacetic acid and purified by preparative RP-HPLC (Combiflash system) (*t*_R_ = 21 min). The combined fractions were basified to pH 8 using a concentrated ammonia solution (28-30 wt. %) and freeze–dried to give compound 11 (DOTA-ADIBO) as a white fluffy solid (280 mg, 357 μmol). Yield: 48%; IR (ATR accessory) ν 3500–3100, 3100–2700, 1627, 1386, 1320, 1219, 1087 cm^−1^; ^1^H NMR (500.13 MHz, CD_3_OD) δ 1.98 (td, 1H, *J* = 6.5, 16.5 Hz), 2.23 (td, 1H, *J* = 6.5, 15.5 Hz), 2.43 (td, 1H, *J* = 7.5, 15.5 Hz), 2.73 (td, 1H, *J* = 7.5, 16.5 Hz), 2.95–3.10 (m, 8H), 3.10–3.30 (m, 4H), 3.35–3.60 (m, 14H), 3.70 (d, 1H, *J* = 14 Hz), 3.60–3.80 (m, 4H), 5.13 (d, 1H, *J* = 14 Hz), 7.25 (d, 1H, *J* = 7.2 Hz), 7.33 (t, 1H, *J* = 7.4 Hz), 7.37 (t, 1H, *J* = 7.3 Hz), 7.46 (m, 3H), 7.64 (m, 2H); ^13^C NMR (125.76 MHz, CD_3_OD) δ 31.3, 32.0, 39.7, 39.8, 50.1 (4C), 52.0 (2C), 52.8 (2C), 55.9 (2C), 56.7, 57.1, 57.7, 108.8, 115.6, 123.7, 124.4, 126.5, 128.6, 128.9, 129.2, 129.6, 130.0, 130.7, 133.4, 149.6, 152.8, 172.5, 174.1, 174.9, 176.0 (3C); HRMS *m*/*z* calculated for C_37_H_48_N_7_O_9_^+^ [M + H]^+^: 734.3508, found: 734.3518.

#### 3.2.9. 2,2′-(7-(1-carboxy-4-oxo-4-((2-(4-(11,12-didehydrodibenzo[b,f]azocin-5(6H)-yl)-4-oxobutanamido)ethyl)amino)butyl)-1,4,7-triazonane-1,4-diyl)diacetic acid, triammonium salt (14 or NODAGA-ADIBO)

To a solution of NODAGA derivative 12 (404 mg, 0.59 mmol) in anisole (400 µL), trifluoroacetic acid (8 mL) was added at −10 °C. The reaction mixture was warmed to room temperature and stirred for 20 h. The solution was evaporated under reduced pressure and the remaining trifluoroacetic acid was removed by co-evaporation with anhydrous dichloromethane (2 × 5 mL). The residue was triturated with anhydrous diethyl ether (3 × 5 mL) and dried under reduced pressure to give the corresponding fully unprotected derivative 13 as an off-white solid (HRMS *m/z* calculated for C_17_H_33_N_5_O_7_ [M + H]^+^: 418.2296, found: 418.2301). To a solution of the latter in anhydrous dimethyl sulfoxide (1.5 mL), under argon, *N*,*N*-diisopropylethylamine (500 µL, 2.94 mmol) was successively added and after 5 min ADIBO active ester S10 (see Supplementary Materials) (237 mg, 0.59 mmol). The reaction mixture was stirred at room temperature for 2.5 h. The resulting solution was neutralised to pH 6 using trifluoroacetic acid and purified by preparative RP-HPLC (Combiflash system) (*t*_R_ = 23.5 min). The combined fractions were basified to pH 8 using a concentrated ammonia solution (28–30 wt. %) and freeze-dried to give compound 14 (NODAGA-ADIBO) as a white fluffy solid (299 mg, 396 µmol). Yield: 67%; IR (ATR accessory) ν 3500–3100, 3100–2700, 1633, 1563, 1448, 1432, 1393, 1206, 1177, 1128 cm^−1^; ^1^H NMR (500.13 MHz, CD_3_OD) δ 1.85–2.00 (m, 2H), 2.05–2.12 (m, 1H), 2.12–2.21 (m, 1H), 2.25–2.39 (m, 2H), 2.40–2.50 (m, 1H), 2.62–2.75 (m, 1H), 2.75–2.28 (m, 16H), 3.36 (q, 1H, *J* = 6.6 Hz), 3.50-3.75 (m, 4H), 3.68 (d, 1H, *J* = 14 Hz), 5.12 (d, 1H, *J* = 14 Hz), 7.24 (dd, 1H, *J* = 1.0, 7.4 Hz), 7.31 (t, 1H, *J* = 7.4 Hz), 7.33–7.38 (m, 1H), 7.45 (m, 3H), 7.60 (m, 1H), 7.64 (d, 1H, *J* = 7.4 Hz); ^13^C NMR (125.76 MHz, CD_3_OD) δ 26.7, 31.4, 31.9, 33.9, 39.7, 40.0, 52.9 (2C), 56.7 (2C), 58.1 (4C), 66.6, 108.8, 115.6, 123.7, 124.4, 126.5, 128.1, 128.9, 129.2, 129.6, 130.0, 130.6, 133.4, 149.5, 152.7, 174.0, 174.9, 175.0, 175.6, 175.7, 176.6; HRMS m/z calculated for C_36_H_45_N_6_O_9_^+^ [M + H]^+^: 705.3243, found: 705.3236.

### 3.3. Immunoconjugation

#### 3.3.1. Azide Functionalisation

N_3_-PEG_4_-NHS S32 (see Supplementary Materials) was conjugated to trastuzumab according to a protocol adapted from Dudkin et al. [31]. In a typical experiment, commercial trastuzumab solution (1 mL, 21 mg/mL) was diluted with PBS (3 mL) and concentrated by centrifugation to obtain a final volume of about 300–500 µL on an Amicon Ultracel 4 filter (50 kDa cut-off). The recovered stock solution (concentration of about 35–63 µg/µL determined by Nanodrop analyses, n = 13) was stored at 4 °C. For the bioconjugation step, trastuzumab (1 mg) from the stock solution was diluted successively with PBS (740 µL) and 1 M sodium carbonate buffer pH 9.2 (155 µL). Then, a solution of N_3_-PEG_4_-NHS S32 in anhydrous DMSO (26.7 µg, 2 µL, 10-fold molar excess compared with trastuzumab) was added. After stirring at room temperature for 15 min, the reaction mixture was transferred to an Amicon Ultracel 4 filter (50 kDa cut-off), and the vial used for bioconjugation was rinsed with PBS (2 × 1 mL). After centrifugation, the residual solution (~100 µL) was purified by SEC-HPLC to remove any trace amounts of unreacted azide ligand S32 or hydrolysed derivative. The collected fraction was concentrated by centrifugation in an Amicon Ultracel 4 filter (50 kDa cut-off) and stored at −20 °C until required. The concentration of the purified N_3_-trastuzumab conjugate was determined by Nanodrop analyses (12.46 ± 4.73 µg/µL, n = 11).

#### 3.3.2. SPAAC Functionalisation

A solution of the appropriate chelator-ADIBO in PBS (150 µL, 50 molar excess based on the immunoconjugate) was added to a solution of N_3_-trastuzumab in PBS (1 mg, 850 µL). The mixture was stirred at 4 °C overnight. The reaction mixture was warmed to room temperature, transferred in an Amicon Ultracel 4 filter (50 kDa cut-off) and the vial used for the coupling step was rinsed with PBS (2 × 1 mL). After centrifugation, the residual solution (~100 µL) was purified by SEC-HPLC to remove any excess of BFC. The collected fraction was concentrated by centrifugation in an Amicon Ultracel 4 filter (50 kDa cut-off) and stored at −20 °C until required. The concentrations of the chelator-trastuzumab solutions were determined by Nanodrop analyses (15.71 ± 4.89, 15.69 ± 6.40 and 15.13 ± 4.99 µg/µL for DOTA-, NODAGA- and 15.5-trastuzumab conjugates, respectively, n = 3).

#### 3.3.3. Determination of the Average Number of Azide and BFC Groups *per* mAb

The mean number of azide and BFC moieties grafted *per* mAb was calculated from MALDI-TOF-MS analysis as the difference of molecular weight between native and modified mAb (using the maximum of the smoothed singly charged species peak). A two-point calibration was performed using the doubly and singly charged species peaks (at around 75 and 150 kDa, respectively) of the IgG1 calibration standard, centrally located near the middle of four sample spots to account for mass drift across the linear sample plate.

### 3.4. Radiochemistry

#### 3.4.1. Effects of Reaction Time, Temperature and Concentration of Immunoconjugates on RCC

Sodium acetate buffer (0.2 M, pH 5.5, 20 µL) and a solution of chelator-trastuzumab conjugate in PBS (0.25–25 µg, 10 µL) were added successively to a solution of [^64^Cu]CuCl_2_ in 0.1 M hydrochloric acid (1 µL, 3.4 MBq). The reaction was left at room temperature or heated to 37 °C. Samples were taken at 15 and 60 min and analysed by radio-ITLC using a 0.1 M citrate buffer (pH 4.5) as eluent (n = 3).

#### 3.4.2. Synthesis of [^64^Cu]Cu-15-5-Trastuzumab, [^64^Cu]Cu-DOTA-Trastuzumab and [^64^Cu]Cu-NODAGA-Trastuzumab for Stability and Binding Studies

Sodium acetate buffer (0.2 M, pH 5.5, 50 µL) and a solution of chelator-trastuzumab conjugate in PBS (30 µg, 1.5–3 µL) were added successively to a solution of [^64^Cu]CuCl_2_ in 0.1 M hydrochloric acid (1.5–2 µL, 4.0 MBq). The reaction was left at room temperature for 15 min (15-5- or NODAGA-trastuzumab) or heated to 37 °C for 60 min (DOTA-trastuzumab). The reaction mixture was quenched by adding a 1 mM aqueous solution of EDTA (1.5 µL). The resulting solution was left at room temperature for 5 min and RCC was determined by radio-ITLC using 1 mM EDTA in PBS as eluent and radio-SEC-HPLC. For DOTA-trastuzumab, free traces of ^64^Cu^2+^ were removed by SEC using a PD-10 column equilibrated and eluted with PBS. 

#### 3.4.3. In Vitro Stability of ^64^Cu-Labelled Conjugates

The in vitro stability of ^64^Cu-labelled chelator-trastuzumab conjugates in 0.1 M phosphate buffer solution and mouse serum was evaluated by radio-ITLC eluted with 1 mM EDTA in PBS. The radiolabelled conjugates (70 µL, 5.1–5.5 MBq), obtained as described in Section 3.4.2., were diluted with 0.1 M phosphate buffer solution (pH 7.4, 150 µL) or with a mixture of 0.1 M phosphate buffer solution (pH 7.4, 150 µL) and mouse serum (220 µL), and the resulting solutions were incubated at 37 °C. Samples were taken after incubation of 1 h and 24 h and the percentage of intact ^64^Cu-labelled immunoconjugates was determined by radio-ITLC eluted with 1 mM EDTA in PBS.

#### 3.4.4. EDTA Challenge Assays

The EDTA competition study was adapted from the protocol described by Frindel et al. [32]. Briefly, each ^64^Cu-labelled immunoconjugate (~5 MBq), obtained according to the protocol described in Section 3.4.2, was divided into four aliquots, which were then diluted with a 0.1 M solution of EDTA (pH 6.5) corresponding 5000, 10,000, 25,000 and 50,000 molar excesses of EDTA vs. mAb, respectively. The final volumes were adjusted to 100 µL with a 0.1 M phosphate buffer solution (pH 7.4) leading to EDTA concentrations ranging from 3.3 to 33 mM. The resulting solutions were then incubated at 37 °C. Samples were taken after incubation for 1 h and 24 h and analysed by radio-ITLC eluted with 1 mM EDTA in PBS.

### 3.5. Cell line and Culture

The BT-474 human breast carcinoma cell line with HER2 overexpression was purchased from Cell Lines Service (CLS Eppelheim, Germany). Cells were cultured in Dulbecco’s Modified Eagle’s Medium (DMEM) F12 (1/1, *v*/*v*) glutamax (Invitrogen, 31331-028), supplemented with 5% FCS, 1% penicillin/streptomycin (Gibco 15140-122) and 0.5% insulin transferrin selenium 100X (Gibco ITS g 41400045). Cells were maintained at 37 °C in an atmosphere of 5% CO_2_ in air. Incubation studies with radiolabelled mAbs were performed under the same conditions.

### 3.6. Determination of the Immunoreactive Fraction

The immunoreactivity of each ^64^Cu-labelled immunoconjugate was determined by a conventional saturation assay according to the method recommended by Denoël et al. [28]. Briefly, increasing concentrations of BT474 cells (1.10^6^, 2.10^6^, 5.10^6^ and 10.10^6^ cells *per* tube) were incubated in 0.5 mL of binding media (25 mM HEPES pH 7, containing 0.2% of BSA, completed with Dulbecco’s Modified Eagle’s Medium (DMEM) F12 (1/1, *v*/*v*) glutamax) with 0.60 pmol of the appropriate immunoconjugate (6.8–27.4 GBq/µmol) in a final volume of 1 mL. After 30 min of gentle shaking, the samples were centrifuged at 460× *g* for 8 min at 4 °C. The supernatants were removed, the cell pellets were washed with PBS containing 0.2% BSA and centrifuged again at 460× *g* for 8 min. The supernatants and pellets collected were recovered separately for radioactivity counting using a γ-counter (Wallac 1480 Wizard^®^ 3‘‘, Perkin Elmer, Villebon sur Yvette, Every, France). The IRF was determined by performing a rectangular hyperbolic fit (one site-specific binding, GraphPad Prism 9.4.1) of the binding curve obtained by plotting B/(B+S) as a function of cell concentration, where B and S are the pellet and supernatant activities counted, respectively. IRF was obtained by extrapolating the quadratic hyperbola value at infinite antigen concentration. All experiments were performed in triplicate.

### 3.7. Statistical Analysis

Statistical analyses were performed on GraphPad Prism 9.4.1 using a one-way multiple comparison on ANOVA test.

## 4. Conclusions

In conclusion, a convenient and reproducible method has been developed for grafting a similar number of 15-5-, NODAGA- and DOTA macrocycles by bioorthogonal cycloaddition to a model mAb, trastuzumab. The ^64^Cu-labelling of the three immunoconjugates was optimised and excellent RCP were obtained (>98%). Compared with the DOTA derivative, the NODAGA- and 15-5-mAb conjugates were radiolabelled with copper-64 with excellent RCC at a lower temperature and with a shorter reaction time. Although all the radioimmunoconjugates showed excellent stability in phosphate buffer or mouse serum, [^64^Cu]Cu-15-5- and [^64^Cu]Cu-NODAGA-trastuzumab presented better resistance to transchelation when challenged by EDTA compared with [^64^Cu]Cu-DOTA-trastuzumab. Finally, no significant differences were found in terms of in vitro antigen recognition, as all the ^64^Cu-labelled immunoconjugates retained high HER2-binding affinity. Taken together, these preliminary results suggest that the 15–5 and NODAGA-macrocycles should be preferred to DOTA for the preparation of ^64^Cu-labelled proteins. To confirm this trend, future studies will focus on characterising the pharmacokinetics and metabolism of [^64^Cu]Cu-15-5-, [^64^Cu]Cu-DOTA and [^64^Cu]Cu-NODAGA-trastuzumab in HER2-positive xenograft model. Finally, the strategy described, which ensures access to a similar average number of chelators *per* macromolecule, irrespective of the chelator studied, while preserving the antigen-recognition capacity, could be very helpful for further similar comparative investigations.

## Figures and Tables

**Table 1:** Average number of chelators per mAb and in vitro immunoreactive fraction (IRF) of ^64^Cu-labelled chelator-trastuzumab conjugates and [^125^I]I-trastuzumab in the HER2-expressing BT474 cell line.

Radioimmunoconjugate	Average Number of Chelators *per* mAb ^a^	IRF (%) ^a^
[^125^I]I-trastuzumab	n.a. ^b^	93.8 ± 0.6 ^c^
[^64^Cu]Cu-DOTA-trastuzumab	3.74 ± 0.18	91.2 ± 6.8
[^64^Cu]Cu-15-5-trastuzumab	3.64 ± 0.16	87.9 ± 1.2
[^64^Cu]Cu-NODAGA-trastuzumab	3.89 ± 0.22	93.8 ± 4.4

^a^ Data are presented as mean values with standard deviation from 2–4 independent experiments in triplicate. ^b^ n.a.: not applicable. ^c^ One experiment performed in triplicate.

## Data Availability

The data presented in this study are available on request from the corresponding author.

## Supplementary Materials

The following supporting information can be downloaded at: https://www.mdpi.com/article/10.3390/molecules28010075/s1. Experimental procedures of compounds **9**, **12, S10** and **S32**, radiolabelling and determination of the IRF of [^125^I]I-trastuzumab, radio-ITLC chromatograms and binding assays plots of all radiolabelled conjugates (Figures S1 and S2, respectively), ^1^H NMR and ^13^C NMR spectra for all synthesised compounds. References [33,34,35,36,37,38,39,40,41,42,43,44,45,46,47,48,49,50,51] are cited in the supplementary materials.

## Sample Availability

Samples of the compounds are not available from the authors.
