# Supplementary material for: Synthesis and In Vitro Comparison of DOTA, NODAGA and 15-5 Macrocycles as Chelators for the 64Cu-Labelling of Immunoconjugates
Source: Molecules. 2022 Dec 22;28(1):75. doi: 10.3390/molecules28010075 (PMC9822305; doi:10.3390/molecules28010075)
Supplement: Supplementary file 1 [file molecules-28-00075-s001.zip › molecules-2079072-supplementary.pdf]

Supporting Information

Synthesis and in vitro comparison of DOTA, NODAGA and 15-5 macrocycles as chelators for the ^{64}Cu-labelling of immunoconjugates

Aurélie Maisonial-Besset[a], Tiffany Witkowski[a], Mercedes Quintana[a], Sophie Besse[a], Vincent Gaumet[a], Axel Cordonnier[a], Cyrille Alliot[b], Aurélien Vidal[b], Caroline Denevault-Sabourin[c], Sébastien Tarrit[a], Sophie Levesque[d], Elisabeth Miot-Noirault[a], Jean-Michel Chezal[a,*]

[a]Université Clermont Auvergne, Inserm, Imagerie Moléculaire et Stratégies Théranostiques, UMR 1240, F-63000 Clermont-Ferrand, France.

[b]GIP Arronax, F-44800 Saint-Herblain, France.

[c] GICC EA7501, Team IMT, Université de Tours, UFR de Médecine, Bâtiment Vialle, 10 Boulevard Tonnellé, BP 3223, CEDEX 01, 37032 Tours, France.

[d]Department of Nuclear Medicine, Jean Perrin Comprehensive Cancer Centre, F-63011 Clermont-Ferrand, France.

*Corresponding author.
E-mail address: j-michel.chezal@uca.fr

Table of contents

1. Synthetic procedures	S3
1.1. Materials and methods	S3
1.2. Synthesis of ADIBO-NHS precursor **S10** and Scheme S1	S3-S4
1.3. Synthesis of benzyl (3-bromopropyl)carbamate (**S11**)	S9
1.4. Synthesis of DOTA precursor **9** and Scheme S2	S10
1.5. Synthesis of NODAGA precursor **12** and Scheme S3	S13
1.6. Synthesis of N_3-PEG_4-NHS derivative **S32** and Scheme S4	S20
2. ^{125}I-labelling of trastuzumab and immunoreactive fraction determination	S23
2.1. General	S23
2.2. Radiolabelling of trastuzumab with iodine-125	S23
2.3. Immunoreactive fraction determination	S23
3. Supplementary figures S1 and S2	S24
4. ^{1}H and ^{13}C NMR spectra of all synthesised compounds	S25
5. References	S62

1. Synthetic procedures

1.1. Materials and methods

All commercially available reagents and solvents were purchased at the following commercial suppliers: Sigma Aldrich, Alpha Aesar, ABX, Acros Organics, Fisher Scientific or Carlo Erba Reagents. Tetrahydrofuran was dried over a Pure Solv™ Micro Solvent Purification System (Sigma-Aldrich) and whenever necessary, other solvents were dried using common techniques [1]. Temperatures indicated in the protocols correspond to the temperature of the oil bath. Analytical thin layer chromatography (TLC) was performed on precoated silica gel 60 F_{254} or neutral aluminium oxide 60 F_{254} plates (Merck or Macherey-Nagel) and visualized under UV light (254 nm) and/or developed with phosphomolybdic acid (8 wt%) in ethanol. Flash column chromatography was performed on silica gel 60A normal phase, 35-70 μm (Merck or SDS) or neutral aluminium oxide 90 standardized, 63-200 μm (Merck). Uncorrected melting points (mp) were recorded on an electrothermal capillary Digital Melting Point Apparatus IA9100 (Bibby Scientific). NMR spectra (200.13 or 500.13 MHz for ^{1}H and 50.32 or 125.76 MHz for ^{13}C) were recorded on a Bruker Avance 200 or 500 instrument with chemical shift values (δ) expressed in parts per million (ppm) relative to residual solvent as standard and coupling constants (*J*) given in Hz. Infrared spectra (IR) were recorded in the range 4000-440 cm^{-1} on a Nicolet IS10 (Fisher Scientific) with attenuated total reflectance (ATR) accessory. Low molecular weight organic compounds were analysed by High-Resolution Mass Spectrometry (HRMS) in positive or negative mode (Waters® Micromass® Q-Tof micro™ Mass Spectrometer) and/or by electrospray ionization mass spectra (ESI-MS) recorded on Esquire (Bruker) spectrometer. For ESI-MS, the analysis of samples was performed at a final concentration of a few tens of pmol/µL. Each ESI-MS spectrum was recorded by averaging of 10 spectra.

1.2. Synthesis of ADIBO-NHS precursor S10

The title compound **S10** was obtained according to slightly modified procedures described by Chadwick, R.C. *et al.* [2] and McNelles, S.A. *et al.* [3].

Scheme S1[a]: Synthesis of ADIBO-NHS precursor **S10**

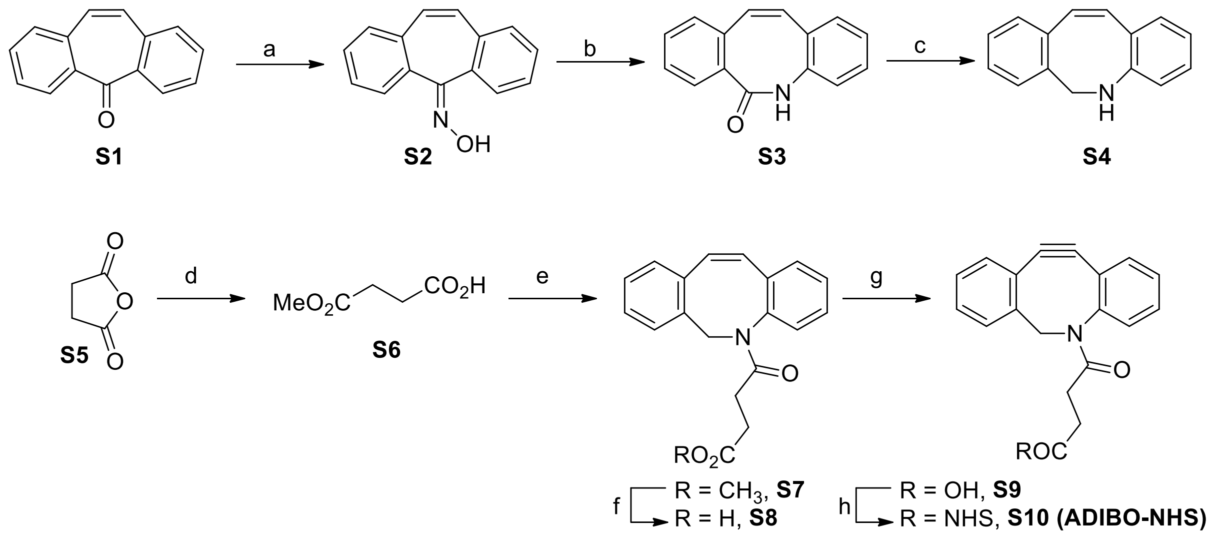

[a]**Reaction conditions:** a) hydroxylamine.HCl, EtOH, pyridine, reflux, 10 h; b) Eaton's reagent, 100 °C, 1 h; c) 4M LAH in Et_2O, Et_2O, reflux, 20 h then 4M LAH in Et_2O, reflux, 22 h; d) NaOMe, MeOH, reflux, 1.5 h; e) (i) oxalyl chloride, DMF, CH_2Cl_2, RT, 3 h; (ii) **S4**, Et_3N, CH_2Cl_2, 0 °C then RT, 2 h; f) (i) LiOH, H_2O, MeOH, reflux, 18 h; (ii) 1M $NaHSO_4$, RT; g) (i) Br_2, CH_2Cl_2, 0 °C, 2 h then RT; (ii) 1M KO*t*Bu in THF, THF, -40 °C,1.5 h then RT; (iii) 1M $NaHSO_4$, RT; h) NHS, EDC.HCl, CH_2Cl_2, RT, 3.5 h.

5*H*-Dibenzo[*a*,*d*]cyclohepten-5-one oxime (**S2**)

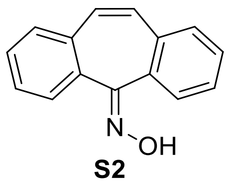

S2

To a solution of hydroxylamine hydrochloride (84.2 g, 1.21 mol, 5 eq.) in absolute ethanol (500 mL), were added, at room temperature and under argon, anhydrous pyridine (130 mL) and commercial 5-dibenzosuberenone (**S1**) (50.0 g, 0.24 mol). The reaction mixture was stirred at reflux for 10 h. At this point, TLC monitoring (Al_2O_3, dichloromethane) showed the complete consumption of starting material. After cooling to room temperature, the mixture was evaporated under reduced pressure. The residue was taken up with deionized water (800 mL) and triturated until precipitation. The precipitate was filtered, washed with deionized water (3 x 200 mL) and dried overnight at 35 °C in a vacuum desiccator to afford compound **S2** as an off-white solid (52.85 g, 0.24 mol), which was used in the next step without further purification. Yield: 99%; mp 189-191 °C (Lit.: 187 °C [4]); IR (ATR accessory) ν 3056, 1433, 1333, 1174 cm^{-1}; ^1H NMR (200.13 MHz, $CDCl_3$) δ 6.92 (s, 2H), 7.30-7.50 (m, 6H), 7.61 (m, 1H), 7.68 (m, 1H), 9.42 (brs, 1H); ^{13}C NMR (50.32 MHz, $CDCl_3$) δ 127.7, 127.8, 128.9, 129.0 (2C), 129.1, 129.2, 129.5, 130.5, 130.6, 130.8, 133.8, 134.6, 135.4, 156.4; ESI-MS *m/z* calculated for $C_{15}H_{12}NO^+$ $[M+H]^+$: 222.09, found: 222.00.

Dibenzo[*b*,*f*]azocine-6(5*H*)-one (**S3**)

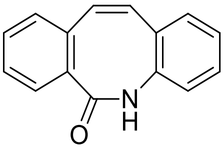

S3

Oxime **S2** (33.0 g, 149.1 mmol) was added portionwise, under argon, to a solution of Eaton's reagent (i.e. 7.5 wt% P_2O_5 in methanesulfonic acid) (200 mL). The reaction was heated at 100 °C for 1 h. After cooling to room temperature, the mixture was poured slowly into cold deionized water (1 L). The formed precipitate was filtered, washed successively with deionized water (3 x 200 mL) and ethyl acetate (2 x 200 mL) and dried overnight at 35 °C in a vacuum desiccator to afford compound **S3** as an off-white solid (29.9 g, 148.7 mol), which was used in the next step without further purification. Yield: 91%; mp 263-265 °C (Lit.: 263-264 °C [5]); IR (ATR accessory) ν 3167, 3026, 1640, 1491, 1395, 1380 cm^{-1}; ^1H NMR (200.13 MHz, DMSO-d_6) δ 6.89 and 7.01 (2H, *J* = 11.6 Hz, AB system), 7.10-7.40 (m, 8H), 9.88 (brs, 1H);^{13}C NMR (50.32 MHz, DMSO-d_6) δ 126.2, 126.4, 127.4, 127.8 (2C), 128.1, 129.0 (2C), 130.2, 132.7, 133.5, 134.5, 136.2, 136.3, 171.8; ESI-MS *m/z* calculated for $C_{15}H_{12}NO^+$ $[M+H]^+$: 222.09, found: 222.00.

5,6-Dihydrodibenzo[*b*,*f*]azocine (**S4**)

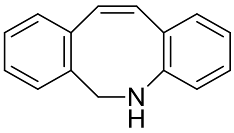

S4

To a solution of amide **S3** (10.00 g, 45.2 mmol) in anhydrous diethyl ether (125 mL) was added dropwise, under argon, a solution of 4 M lithium aluminium hydride in anhydrous diethyl ether (28.25 mL, 113 mmol, 2.5 eq). The reaction mixture was refluxed for 20 h. After cooling to room temperature, a solution of 4 M lithium aluminium hydride in anhydrous diethyl ether (10 mL, 40 mmol) was added and the solution was refluxed for 22 h. At this point, TLC monitoring (SiO_2, cyclohexane/ethyl acetate, 7/3, v/v) showed complete disappearance of the starting material. After cooling to room temperature, the mixture was slowly treated dropwise with successively deionized water (4.5 mL), 1 N aqueous sodium hydroxide solution (4.5 mL) and deionized water (4.5 mL). The reaction mixture was stirred until gas evolution ceased. The inorganic precipitate was then filtered and washed successively with diethyl ether (2 x 120 mL) and dichloromethane (120 mL). The filtrate was dried over magnesium sulfate, filtered and evaporated under reduced pressure to give compound **S4** as a yellow solid (8.36 g, 40.3 mmol), which was used in the next step without further purification. Yield: 89%;

mp 107-109 °C (Lit.: 108-109 °C [5]); IR (ATR accessory) ν 3400, 3049, 2918, 1597, 1493, 1450, 1333, 1270 cm^{-1}; ^{1}H NMR (200.13 MHz,$CDCl_3$) δ 4.29 (brs, 1H), 4.59 (s, 2H), 6.36 (d, 1H, *J* = 13 Hz), 6.47 (d, 1H, *J* = 7.9 Hz), 6.55 (d, 1H, *J* = 13 Hz), 6.60 (t, 1H, *J* = 7.5 Hz), 6.89 (t, 1H, *J* = 7.5 Hz), 6.97 (d, 1H, *J* = 7.5 Hz), 7.23 (m, 4H); ^{13}C NMR (50.32 MHz, $CDCl_3$) δ 49.5, 117.7, 117.9, 121.7, 127.4 (2C), 127.6, 128.0, 128.9, 130.1, 132.7, 134.7, 138.1, 139.2, 147.1; ESI-MS *m/z* calculated for $C_{15}H_{14}N^+$ $[M+H]^+$: 208.11, found: 208.02.

4-Methoxy-4-oxobutanoic acid (**S6**)

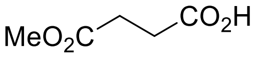

S6

This compound was obtained according to the procedure described by von Wantoch Rekowski, M. *et al*. [6]. To a solution of succinic anhydride (**S5**) (10.0 g, 100 mmol) in anhydrous methanol (150 mL) was added, under argon, sodium methylate (54 mg, 1 mmol). The reaction mixture was heated at reflux for 1.5 h. After cooling to room temperature, the solution was evaporated under reduced pressure to give compound **S6** as a white solid (13.11 g, 99.2 mmol), which was used in the next step without further purification. Yield: 99%; mp 56-58 °C (Lit.: 57 °C [6]); IR (ATR accessory) ν 3300-2700, 2700-2300, 1727, 1687, 1440, 1417, 1366, 1344, 1254, 1232, 1198, 1173 cm^{-1}; ^{1}H NMR (200.13 MHz, $CDCl_3$) δ 2.65 (m, 4H), 3.68 (s, 3H), 10.90 (brs, 1H); ^{13}C NMR (50.32 MHz, $CDCl_3$) δ 28.7, 29.0, 52.1, 172.8, 178.5; ESI-MS *m/z* calculated for $C_5H_7O_4^-$ $[M-H]^-$: 131.03, found: 131.02.

Methyl 4-(dibenzo[*b*,*f*]azocin-5(6*H*)-yl)-4-oxobutanoate (**S7**)

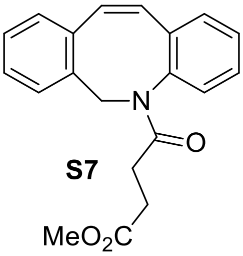

S7

To a solution of 4-methoxy-4-oxobutanoic acid (**S6**) (2.39 g, 18.1 mmol) in anhydrous dichloromethane (10 mL) were successively added, under argon, *N*,*N*-dimethylformamide (30 µL) and oxalyl chloride (1.94 mL, 22.6 mmol). The reaction mixture was stirred at room temperature for 3 h until gas evolution ceased. The solvent was removed under reduced pressure, the remaining material was dissolved in anhydrous dichloromethane (10 mL) and the solvent was evaporated once again. The residue was dissolved in anhydrous dichloromethane (20 mL) and added dropwise, at 0 °C and under argon, to a solution of 5,6-dihydrodibenzo[*b*,*f*]azocine (**S4**) (2.50 g,

12.1 mmol) and triethylamine (3.4 mL, 24.4 mmol) in anhydrous dichloromethane (80 mL). After warming to room temperature, the reaction was stirred for 2 h. The mixture was then washed successively with a 2 N aqueous sodium hydroxide solution (3 x 40 mL), a 2 N hydrochloric acid solution (3 x 40 mL) and brine (90 mL). The organic layer was dried over magnesium sulfate, filtered and evaporated under reduced pressure. The residue was purified by column chromatography (SiO_2, ethyl acetate/cyclohexane, 3/7, v/v) to give compound **S7** as an off-white solid (2.96 g, 9.21 mmol). Yield: 76%; mp 112-113 °C (Lit.: 108 °C [4]); R_f = 0.40 (SiO_2, ethyl acetate/cyclohexane, 3/7, v/v); IR (ATR accessory) ν 1733, 1645, 1490, 1393, 1362, 1271, 1211, 1162 cm^{-1}; ^{1}H NMR (200.13 MHz, $CDCl_3$) δ 1.90-2.10 (m, 1H), 2.35-2.70 (m, 3H), 3.62 (s, 3H), 4.25 (d, 1H, J = 15 Hz), 5.51 (d, 1H, J = 15 Hz), 6.61 (d, 1H, J = 13 Hz), 6.79 (d, 1H, J = 13 Hz), 7.14 (m, 3H), 7.26 (m, 5H); ^{13}C NMR (50.32 MHz, $CDCl_3$) δ 29.2, 29.7, 51.8, 54.6, 127.1, 127.4 (2C), 128.2, 128.4, 128.7, 130.3, 131.0, 132.0, 132.8, 134.7, 136.0, 136.6, 140.6, 171.0, 173.6; ESI-MS *m/z* calculated for $C_{20}H_{19}NNaO_3^+$ $[M+Na]^+$: 344.13, found: 344.13.

4-(Dibenzo[*b*,*f*]azocin-5(6*H*)-yl)-4-oxobutanoic acid (**S8**)

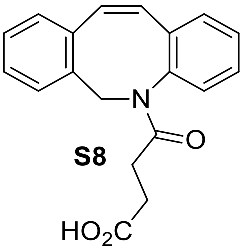

S8

To a solution of ester **S7** (2.50 g, 7.78 mmol) in methanol (50 mL) was added a solution of lithium hydroxide (1.12 g, 46.8 mmol) in deionized water (25 mL). The reaction was heated at reflux for 18 h. After cooling to room temperature, the mixture was diluted with a 1 M aqueous sodium hydrogen sulfate solution (50 mL) and then extracted with dichloromethane (3 x 50 mL). The combined organic layers were dried over magnesium sulfate, filtered and evaporated under reduced pressure. The resulting sticky oil was triturated with anhydrous diethyl ether (20 mL) to give compound **S8** as a white solid (2.07 g, 6.74 mmol), which was used in the next step without further purification. Yield: 87%; mp 133-134 °C; IR (ATR accessory) ν 3100-2800, 1695, 1646, 1392, 1256, 1231 cm^{-1}; ^{1}H NMR (200.13 MHz, $CDCl_3$) δ 1.90-2.10 (m, 1H), 4.28 (d, 1H, J = 15 Hz), 5.52 (d, 1H, J = 15 Hz), 6.61 (d, 1H, J = 12.8 Hz), 6.80 (d, 1H, J = 12.8 Hz), 7.10-7.30 (m, 8H), 9.55 (brs, 1H); ^{13}C NMR (50.32 MHz, $CDCl_3$) δ 29.6 (2C), 54.7, 127.2, 127.4 (2C), 128.2, 128.4, 128.8, 130.2, 130.9, 131.9, 132.9, 134.3, 135.9, 136.6, 140.3, 171.6, 177.6; ESI-MS *m/z* calculated for $C_{19}H_{17}NO_3Na^+$ $[M+Na]^+$: 330.11, found: 330.13.

5-(11,12-Didehydrodibenzo[*b*,*f*]azocin-5(6*H*)-yl)-4-oxobutanoic acid (**S9**)

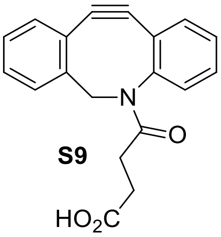

To a solution of compound **S8** (1.80 g, 5.86 mmol) in anhydrous dichloromethane (80 mL), was added dropwise, at 0 °C and under argon, bromine (900 µL, 17.6 mmol). The resulting solution was stirred at 0 °C for 2 h. The progress of the reaction was monitored by ^{1}H NMR. After completion, the reaction mixture was allowed to warm to room temperature. Dichloromethane (100 mL) was then added and the mixture was successively washed with a saturated aqueous sodium hydrogen sulfite solution (3 x 100 mL), deionized water (100 mL) and brine (100 mL). The organic layer was dried over magnesium sulfate, filtered and evaporated under reduced pressure. The resulting grey solid (dibrominated derivative) was diluted, under argon, with anhydrous tetrahydrofuran (100 mL) and cooled to -40 °C. A solution of 1 M potassium *tert*-butylate in anhydrous tetrahydrofuran (21 mL, 21 mmol) was then slowly added and the reaction mixture was stirred at -40 °C for 1.5 h. The progress of the reaction was monitored by ^{1}H NMR. After completion, the reaction was allowed to warm to room temperature, quenched with a 1 M aqueous sodium hydrogen sulfate solution until pH = 1 (ca. 25 mL) and extracted with dichloromethane (3 x 100 mL). The combined organic layers were dried over magnesium sulfate, filtered and evaporated under reduced pressure. The crude was taken up with a small volume of anhydrous diethyl ether and the resulting precipitate was filtered to give compound **S9** as an off-white solid (1.58 g, 5.17 mmol), which was used in the next step without further purification. Yield: 88%; mp 172-174 °C (Lit.: 170 °C [2]); IR (ATR accessory) ν 3100-2800, 1704, 1650, 1423, 1395, 1321, 1203 cm^{-1}; ^{1}H NMR (200.13 MHz, DMSO-d$_6$) δ 1.76 (m, 1H), 2.27 (m, 2H), 2.57 (m, 1H), 3.61 (d, 1H, J = 13.9 Hz), 5.03 (d, 1H, J = 13.9 Hz), 7.20-7.70 (m, 8H), 12.03 (brs 1H); ^{13}C NMR (50.32 MHz, DMSO-d$_6$) δ 29.0, 29.3, 55.0, 108.1, 114.3, 121.6, 122.6, 125.2, 126.9, 127.7, 128.1, 128.2, 129.0, 129.7, 132.4, 148.5, 151.5, 170.8, 170.6; ESI-MS *m/z* calculated for $C_{19}H_{15}NNaO_3^+$ [M+Na]$^+$: 328.09, found: 328.16.

Succinimidyl 4-(11,12-didehydrodibenzo[*b*,*f*]azocin-5(6*H*)-yl)-4-oxobutanoate (**S10, ADIBO-NHS**)[3]

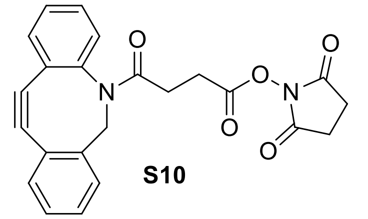

S10

To a solution of acid **S9** (1.00 g, 3.28 mmol) in anhydrous dichloromethane (50 mL) were added, under argon, *N*-hydroxysuccinimide (565 mg, 4.91 mmol) and *N*-ethyl-*N'*-(3-dimethylaminopropyl)carbodiimide, hydrochloride salt (942 mg, 4.91 mmol). The reaction mixture was stirred at room temperature for 3.5 h. The solution was then successively washed with a 5% aqueous citric acid solution (2 x 50 mL), a 5% aqueous sodium hydrogen carbonate solution (2 x 50 mL) and brine (50 mL). The organic layer was dried over magnesium sulfate, filtered and evaporated under reduced pressure. The residue was purified by column chromatography (SiO_2, ethyl acetate/cyclohexane, 1/1, v/v) to give compound **S10** as an off-white solid (1.22 g, 3.03 mmol). Yield: 93%; mp 162-164 °C; R_f = 0.32 (SiO_2, ethyl acetate/cyclohexane, 1/1, v/v); IR (ATR accessory) ν 1734, 1656, 1204, 1069 cm^{-1}; ^{1}H NMR (200.13 MHz, $CDCl_3$) δ 2.08 (m, 1H), 2.55-3.10 (m, 7H), 3.68 (d, 1H, *J* = 13.8 Hz), 5.18 (d, 1H, *J* = 13.8 Hz), 7.20-7.50 (m, 7H), 7.69 (d, 1H, *J* = 7.6 Hz); ^{13}C NMR (50.32 MHz, $CDCl_3$) δ 25.6 (2C), 26.5, 29.3, 55.7, 107.7, 115.1, 122.8, 123.1, 125.6, 127.3, 127.9, 128.5 (2C), 128.7, 129.2, 132.4, 147.9, 151.1, 168.4, 169.0 (2C), 170.4; ESI-MS *m/z* calculated for $C_{23}H_{18}N_2O_5Na^+$ $[M+Na]^+$: 425.11, found: 425.09.

1.3. Synthesis of benzyl (3-bromopropyl)carbamate (S11)

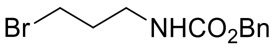

S11

This compound was obtained according to slight modifications of the procedure described by Wei, W.H. *et al*. [7]. To a solution of 3-bromopropylamine hydrobromide salt (3.00 g, 13.7 mmol) in a mixture of water and 1,4-dioxane (14 mL, 1/1, v/v) was slowly added dropwise over 30 min a solution of benzyl chloroformate (2,24 mL, 15.8 mmol) in 1,4-dioxane (7 mL) while maintaining the pH at 6-7 by addition of a 3.5 M aqueous potassium carbonate solution. After addition, the pH was adjusted to 7-8 and the reaction mixture was stirred at room temperature for 1 h. Excess of benzyl chloroformate was hydrolysed by addition of a 2 N aqueous sodium hydroxide solution (1.05 mL). After stirring at room temperature for 2 h, the mixture was extracted with diethyl ether (3 x 45 mL) and the combined organic layers were dried over magnesium sulfate, filtered and evaporated under reduced pressure. The residue was purified by

column chromatography (SiO_2, ethyl acetate/cyclohexane, 1/10, v/v) to give compound **S11** as a colourless oil (3.44 g, 12.6 mmol). Yield: 92%; R_f = 0.11 (SiO_2, ethyl acetate/cyclohexane, 1/10, v/v); IR (ATR accessory) ν 3500-3250, 2949, 2900-2500, 2435, 1693, 1522, 1454, 1241, 1128, 1076, 1041 cm^{-1}; ^1H NMR (200.13 MHz, $CDCl_3$) δ 2.06 (quint, 2H, J = 6.4 Hz), 3.34 (q, 2H, J = 6.4 Hz), 3.42 (t, 2H, J = 6.4 Hz), 5.04 (m, 1H), 5.09 (s, 2H), 7.35 (s, 5H); ^{13}C NMR (50.32 MHz, $CDCl_3$) δ 30.8, 32.5, 39.5, 66.9, 128.3 (3C), 128.6 (2C), 136.5, 156.6.

1.4. Synthesis of DOTA precursor 9

Scheme S2^a: Synthesis of DOTA precursor **9**

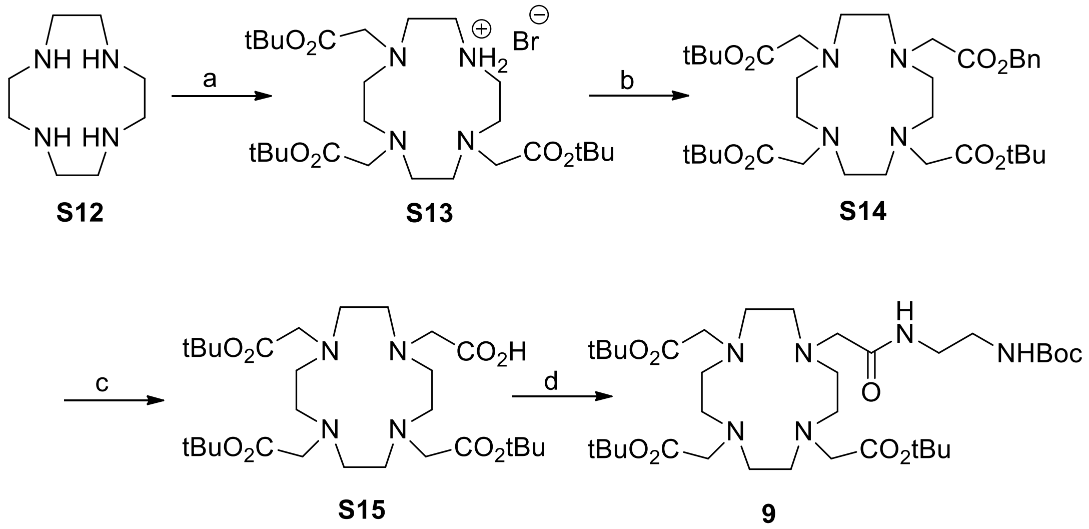

^{a}**Reaction conditions:** a) NaOAc, DMA, *tert*-butyl 2-bromoacetate, RT, 60 h; b) benzyl 2-bromoacetate, K_2CO_3, CH_3CN, 70 °C, 18.5 h; c) H_2, Pd/C 10%, MeOH, RT, 22 h; d) HOBt.H_2O, EDC.HCl, *tert*-butyl *N*-(2-aminoethyl)carbamate, CH_2Cl_2, RT, 45 h.

Tri-*tert*-butyl 2,2',2''-(1,4,7,10-tetraazacyclododecane-1,4,7-triyl)triacetate hydrobromide salt (**S13**)

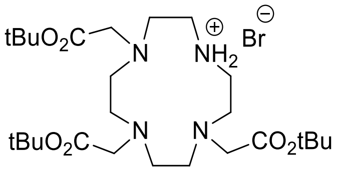

S13

This compound was obtained according to a modified procedure of the protocol described by Moore, D.A. *et al*. [8]. To a solution of cyclen (**S12**) (5.00 g, 29.0 mmol) in *N*,*N*-dimethylacetamide (40 mL) was added portionwise, under argon, sodium acetate (13.03 g, 95.8 mmol) (exothermic reaction). The suspension was stirred for 30 min until return back to room temperature. A solution of *tert*-butyl 2-bromoacetate (18.7

g, 95.9 mmol) in *N*,*N*-dimethylacetamide (20 mL) was then slowly added dropwise while the internal temperature was maintained below 35 °C. After stirring at room temperature for 60 h, anhydrous diethyl ether (20 mL) was added, and the reaction mixture was cooled to -10 to -15 °C. After stirring at this temperature for 2 h, the resulting precipitate was collected by filtration and washed successively with cold (-10 to -15 °C) *N*,*N*-dimethylacetamide (10 mL) and cold (-10 to -15 °C) diethyl ether (2 x 25 mL). The solid was dissolved in chloroform (100 mL) and washed successively with deionized water (2 x 15 mL) and a saturated aqueous sodium bromide solution (15 mL). The organic layer was dried over magnesium sulfate, filtered and evaporated under reduced pressure to reach approximately 40 g. The solution was diluted with cyclohexane (80 mL) and stirred at room temperature for 3 h and then at -10 to -15 °C for 2 h. The resulting precipitate was filtered, washed with a mixture of cold (-10 to -15 °C) cyclohexane/chloroform solution (4/1, v/v, 25 mL) and dried overnight at 35 °C in a vacuum desiccator to give compound **S13** as a white solid (9.33 g, 15.7 mmol), which was used in the next step without further purification. Yield: 54%; mp 193-195 °C (Lit.: 190-191 °C [9]); IR (ATR accessory) ν 2937, 2868, 2737, 1717, 1365, 1271, 1254, 1162, 1146, 1116, 1100 cm^{-1}; ^{1}H NMR (500.13 MHz, $CDCl_3$) δ 1.29 (s, 27H), 2.71 (m, 4H), 2.76 (m, 8H), 2.94 (brs, 4H), 3.13 (s, 2H), 3.23 (s, 4H), 9.77 (m, 2H); ^{13}C NMR (125.76 MHz, $CDCl_3$) δ 27.9 (6C), 28.0 (3C), 47.2 (2C), 48.9 (2C), 51.0 (4C), 57.8 (3C), 81.3 (2C), 81.5, 169.4, 170.3 (2C).

Tri-*tert*-butyl 2,2',2''-(10-(2-benzyloxy-2-oxoethyl)-1,4,7,10-tetraazacyclododecane-1,4,7-triyl)triacetate (**S14**)

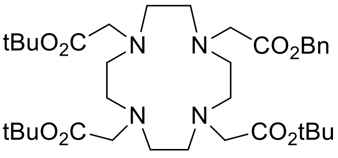

S14

This compound was obtained according to slight modifications of the procedure described by Strauch, R.C. *et al*. [10]. To a solution of compound **S13** (2.50 g, 3.43 mmol) in anhydrous acetonitrile (64 mL) were successively added under argon potassium carbonate (1.74 g, 12.6 mmol) and benzyl 2-bromoacetate (798 µL, 5.04 mmol). The reaction mixture was heated at 70 °C for 18.5 h. After cooling to room temperature, the solid was removed by filtration and the filtrate was evaporated under reduced pressure. The residue was purified by column chromatography (SiO_2, dichloromethane→dichloromethane/methanol, 95/5, v/v) to give compound **S14** as an off-white solid (2.74 g, 4.13 mmol). Yield: 98%; mp 93-94 °C; R_f = 0.13 (SiO_2, dichloromethane/methanol, 95/5, v/v); IR (ATR accessory) ν 2976, 2822, 1722, 1454, 1367, 1308, 1259, 1224, 1154, 1104 cm^{-1}; ^{1}H NMR (500.13 MHz, CD_3CN) δ 1.44 (s,

18H), 1.46 (s, 9H), 1.90-3.70 (set of broad and multiple peaks, 24H), 5.15 (m, 2H), 7.36 (m, 5H); ^{13}C NMR (125.76 MHz, CD_3CN) δ 28.2 (9C), 48.9 (m, 4C), 53.4 (m, 4C), 55.3, 55.7, 56.3, 56.4 (2C), 67.6, 82.6, 82.7 (2C), 129.3 (2C), 129.5 (2C), 136.7, 174.0, 174.1 (2C), 174.6; HRMS *m/z* calculated for $C_{35}H_{59}N_4O_8^+$ $[M+H]^+$: 663.4327, found: 663.4319.

2-(4,7,10-Tris(2-(*tert*-butoxy)-2-oxoethyl)-1,4,7,10-tetraazacyclododecan-1-yl)acetic acid (**S15**)

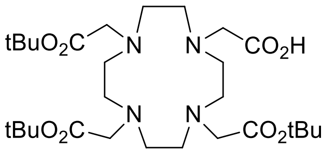

S15

To a degassed solution of benzyl derivative **S14** (1.14 g, 1.72 mmol) in methanol (25 mL) was added Pd/C 10% (114 mg). After stirring at room temperature for 22 h under hydrogen atmosphere, the suspension was filtered over 0.45 µm PTFE membrane filter and the filtrate was evaporated under reduced pressure to provide compound **S15** as an off-white solid (0.97 g, 1.69 mmol), which was used in the next step without further purification. Yield: 98%; mp 124-126 °C; (Lit.: 129-131 °C [11]); IR (ATR accessory) ν 3600-3100, 2976, 2905, 2820, 1726, 16.35, 1393, 1368, 1306, 1225, 1156, 1118, 1106 cm^{-1}; ^{1}H NMR (200.13 MHz, $CDCl_3$) δ 1.29 (s, 27H), 1.90-3.70 (broad and multiple peaks, 24H), 8.22 (brs, 1H); ^{13}C NMR (125.76 MHz, CD_3CN) δ 28.3 (9C), 48.1-50.0 (4C), 52.3-54.2 (4C), 56.2, 56.3, 56.7 (2C), 82.7 (3C), 173.9 (3C), 175.4; HRMS *m/z* calculated for $C_{28}H_{53}N_4O_8^+$ $[M+H]^+$: 573.3858, found: 573.3857.

Tri-*tert*-butyl 2,2',2''-(10-(2-((2-((*tert*-butoxycarbonyl)amino)ethyl)amino)-2-oxoethyl)-1,4,7,10-tetraazacyclododecane-1,4,7-triyl)triacetate (**9**)

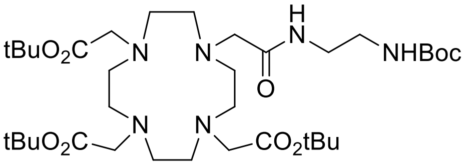

9

To a solution of acid **S15** (896 mg, 1.56 mmol) in anhydrous dichloromethane (40 mL) were successively added under argon 1-hydroxybenzotriazole monohydrate (233 mg, 1.72 mmol), *N*-(3-dimethylaminopropyl)-*N'*-ethylcarbodiimide hydrochloride salt (390 mg, 2.03 mmol) and after 5 min, a solution of *tert*-butyl *N*-(2-aminoethyl)carbamate [12] (325 mg, 2.03 mmol) in anhydrous dichloromethane (5 mL). After stirring at room temperature for 45 h, the reaction mixture was washed successively with a 2 N aqueous sodium hydroxide solution (2 x 50 mL) and brine (2 x 50 mL), dried over

sodium sulfate, filtered and evaporated under reduced pressure. The residue was purified by column chromatography (SiO_2, dichloromethane/ethanol/ammonium hydroxide, 90/10/0.2, v/v/v) to give compound **9** as a white solid (931 mg, 1.30 mmol). Yield: 83%; mp 102-104 °C; R_f = 0.13 (SiO_2, dichloromethane/ethanol/ammonium hydroxide, 90/10/0.2, v/v/v); IR (ATR accessory) ν 3500-3100, 2976, 2825, 1726, 1667, 1366, 1308, 1226, 1159, 1121, 1106 cm^{-1}; ^1H NMR (500.13 MHz, $CDCl_3$) δ 1.38 (s, 9H), 1.42 (s, 27H), 1.90-3.10 (m, 20H), 3.15-3.50 (m, 8H), 6.29 (brs, 1H), 9.03 (brs, 1H); ^{13}C NMR (125.76 MHz, CD_3CN) δ 28.2 (9C), 28.7 (3C), 40.4, 40.5, 47.0-54.6 (8C), 56.4 (3C), 57.0, 78.6, 82.0 (2C), 82.2, 156.7, 172.8, 173.2 (2C), 173.6; HRMS *m/z* calculated for $C_{35}H_{67}N_6O_9^+$ $[M+H]^+$: 715.4964, found: 715.4956.

1.5. Synthesis of NODAGA precursor 12

Scheme S3[a]: Synthesis of NODAGA precursor **12**

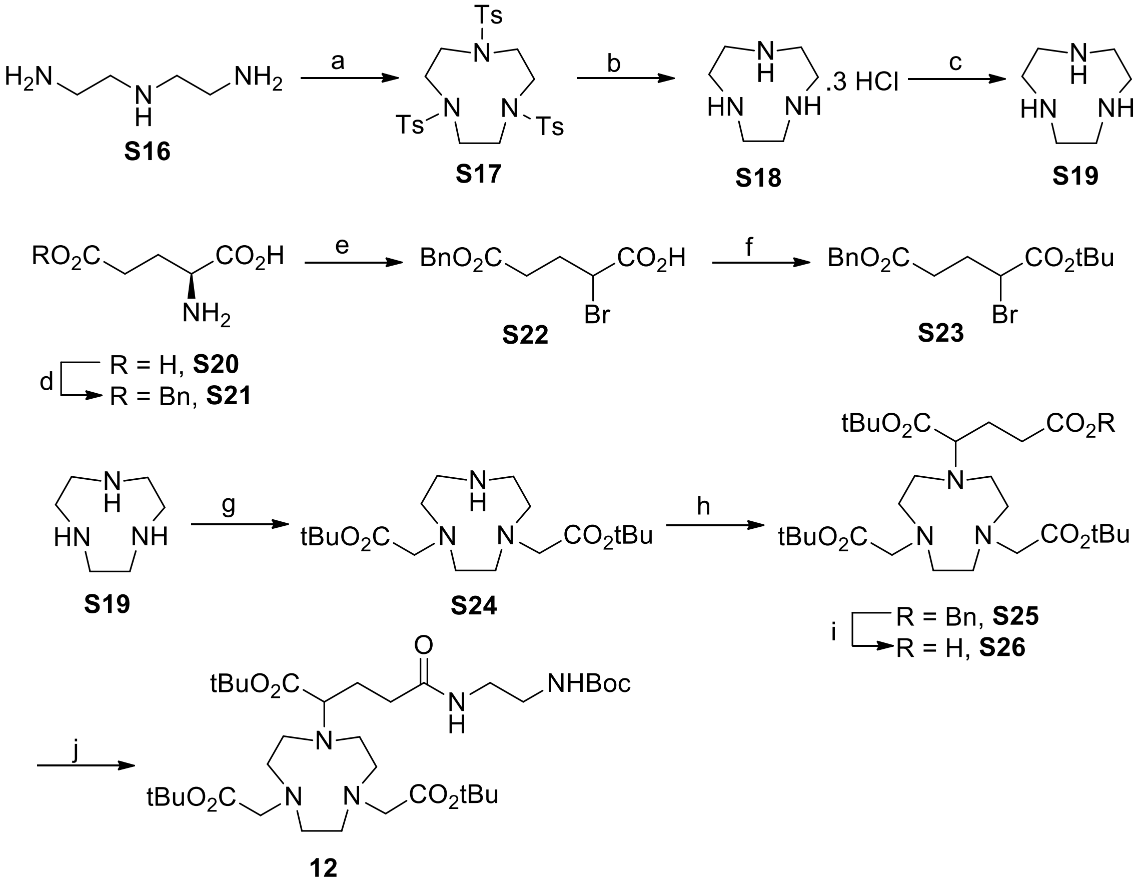

[a]Reaction conditions: a) (i) 1.05 M aq. K_2CO_3, TsCl, 90 °C, 1.5 h; (ii) NaOH, 1 M aq. TBAB, 1,2-dibromoethane, toluene, reflux, 10.5 h; b) (i) conc. H_2SO_4, 140 °C, overnight; (ii) H_2O, reflux, 1 h then conc. HCl, 0 °C, 1.5 h; c) aq. NaOH, toluene, Dean-Stark, overnight; d) BnOH, conc. H_2SO_4, H_2O, 70 °C, 45 min; e) 1 M aq. HBr, $NaBr.2H_2O$, $NaNO_2$, 0 °C, 4 h; f) *tert*-butyl acetate, 70% aq. $HClO_4$, RT, 40 h; g) *tert*-butyl 2-bromoacetate, $CHCl_3$, RT, 60 h; h) **S23**, K_2CO_3, CH_3CN, 40 °C, 24 h; i) *i*PrOH, H_2, Pd/C 10%, RT, 20 h; j) $HOBt.H_2O$, EDC.HCl, *tert*-butyl *N*-(2-aminoethyl)carbamate, CH_2Cl_2, RT, 15 h.

1,4,7-Tritosyl-1,4,7-triazacyclononane (**S17**)

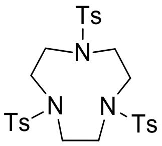

S17

This compound was obtained according to the procedure described by Montagner, D. et al. [13]. Diethylenetriamine (**S16**) (10.5 mL, 96.7 mmol) and tosyl chloride (55.36 g, 0.29 mol) were added successively to a 1.05 M aqueous potassium carbonate solution (300 mL). The reaction mixture was stirred at 90 °C for 1.5 h. After cooling to room temperature, toluene (200 mL), sodium hydroxide (34.92 g, 0.873 mol), a 1 M aqueous tetrabutylammonium bromide solution (10 mL) and 1,2-dibromoethane (15.0 mL, 0.174 mol) were added successively. The resulting solution was stirred at reflux for 10.5 h. After cooling to room temperature, 1,2-dibromoethane (15.0 mL, 0.174 mol) was added and the solution was stirred at reflux for 7 h. The last procedure was repeated once again with a reflux time of 14 h. After cooling to room temperature, the solid was filtered, washed with deionized water (2 x 150 mL) and dried overnight at 35 °C in a vacuum desiccator to give compound **S17** as a white solid (44.32 g, 74.9 mmol), which was used in the next step without further purification. Yield: 77%; mp 216-217 °C (Lit.: 218-220 °C [14]); IR (ATR accessory) ν 1342, 1325, 1154, 1088 cm^{-1}; ^{1}H NMR (200.13 Hz, $CDCl_3$) δ 2.43 (s, 9H), 3.41 (s, 12H), 7.32 (d, 6H, *J* = 8.1 Hz), 7.69 (d, 6H, *J* = 8.1 Hz); ^{13}C NMR (50.32 Hz, $CDCl_3$) δ 21.7 (3C), 52.0 (6C), 127.6 (6C), 130.0 (6C), 134.7 (3C), 144.0 (3C); ESI-MS *m/z* calculated for $C_{27}H_{33}N_3NaO_6S_3^+$ $[M+Na]^+$: 614.14, found: 614.21.

1,4,7-Triazacyclononane, trihydrochloride salt (**S18**)

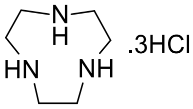

S18

A solution of compound **S17** (5.00 g, 6.54 mmol) in concentrated sulfuric acid (5 mL) was stirred at 140 °C overnight. After cooling to room temperature, the reaction mixture was poured dropwise in methanol (30 mL). The resulting solution was stored overnight at 4 °C. The brown precipitate formed was filtered and washed with diethyl ether (20 mL). The solid was dissolved in deionized water (10 mL) and the solution was refluxed for 1 h. After cooling to room temperature, concentrated aqueous hydrochloric acid solution (8 mL) was added and the resulting solution was stored at 0 °C for 1.5 h. The grey precipitate was filtered, washed with diethyl ether (2 x 10 mL) and dried overnight at 35 °C in a vacuum desiccator to give compound **S18** as a grey solid (1.29 g, 5.41

mmol), which was used in the next step without further purification. Yield: 64%; mp 282-284 °C (Lit.: 280-281 °C [15]); IR (ATR accessory) ν 3100-2900, 2900-2500, 2433, 1588, 1431, 1413, 1373, 985 cm^{-1}; ^1H NMR (200.13 MHz, D_2O+1,4-dioxane) δ 3.42 (s, 12H); ^{13}C NMR (50.32 MHz, D_2O+1,4-dioxane) δ 42.7 (6C).

1,4,7-Triazacyclononane (**S19**)

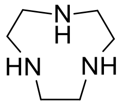

S19

This compound was obtained according to the procedure described by Montagner, D. *et al*. [16]. To a solution of sodium hydroxide (2.33 g, 58.3 mmol) in deionized water (8 mL) was added successively 1,4,7-triazacyclononane hydrochloride salt (**S18**) (4.63 g, 19.4 mmol) and toluene (30 mL). The reaction mixture was refluxed overnight in a Dean-Stark apparatus in order to remove water. Toluene was decanted and the residue was taken up with fresh toluene (10 mL). Sodium hydroxide (1.11 g, 27.8 mmol) was added and the resulting solution was heated at reflux for 1.5 h and then filtered hot. The organic layers were combined, dried over magnesium sulfate, filtered and evaporated under reduced pressure to give compound **S19** as a yellow oil (2.50 g, 19.3 mmol), which solidified on standing (white solid, very hygroscopic). This compound was used in the next step without further purification. Yield: quant.; mp 88-90 °C (Lit.: 62 °C [15]); IR (ATR accessory) ν 3300, 3050-2400, 1458, 1380, 1347, 1277, 1160, 1068 cm^{-1}; ^1H NMR (200.13 MHz, $CDCl_3$) δ 2.17 (s, 3H), 2.68 (s, 12H); ^{13}C NMR (50.32 MHz, $CDCl_3$) δ 45.7 (6C).

5-Benzyl L-glutamate (**S21**)

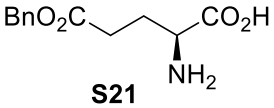

S21

This compound was obtained according to slight modifications of the procedure described by Yoshida, M. *et al*. [17]. To a solution of L-glutamic acid (**S20**) (14.7 g, 0.10 mol) in deionized water (6.5 mL) was slowly added dropwise concentrated sulfuric acid (9.8 g) followed by benzyl alcohol (12.0 g, 0.11 mmol). The reaction mixture was stirred at 70 °C for 45 min. After evaporation of the solvent under reduced pressure at 70 °C for 4 h, the hot resulting sticky residue was poured into a 500 mL beaker containing a solution of sodium hydrogen carbonate (16.8 g, 0.20 mol) in deionized water (50 mL) precooled at 0 °C. The remaining syrup in the flask was washed off with deionized water (3 x 30 mL) and the combined reaction mixture was stirred at room temperature overnight. After filtration and recrystallization of the solid from deionized

water, the white precipitate was washed with ethyl acetate (2 x 30 mL) and dried overnight at 30 °C in a vacuum desiccator to give compound **S21** as a white solid (10.66 g, 44.9 mmol). Yield: 45%; mp 180-181 °C (lit.: 188-189 °C [18]); IR (ATR accessory) ν 3400-2400, 1722, 1577, 1515, 1451, 1410, 1387, 1308, 1169 cm^{-1}; ^{1}H NMR (200.13 MHz, conc. HCl/D_2O) δ 2.25 (m, 2H), 2.69 (t, 2H, *J* = 7.2 Hz), 4.14 (t, 1H, *J* = 6.6 Hz), 5.17 (s, 2H), 7.44 (m, 5H); ^{13}C NMR (50.32 MHz, conc. HCl/D_2O with 1,4-dioxane as internal reference) δ 25.4, 30.2, 52.5, 67.8, 128.9 (3C), 129.3 (2C), 136.0, 171.8, 174.6; ESI-MS *m/z* calculated for $C_{12}H_{16}NO_4^+$ $[M+H]^+$: 238.11, found: 238.08.

2-Bromopentandioic acid 5-benzyl ester (**S22**)

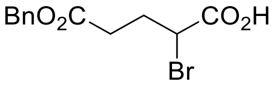

S22

This compound was obtained according to slight modifications of the procedure described by Lamarque, L. *et al*. [19]. To a solution of 5-benzyl L-glutamate (**S21**) (5.00 g, 21.1 mmol) and sodium bromide dihydrate (8.00 g, 57.6 mmol) in 1 M hydrobromic acid solution (125 mL) was added dropwise, at 0 °C, a solution of sodium nitrite (2.25 g, 39.9 mmol) in deionized water (25 mL) keeping the reaction temperature below 5 °C. After stirring at 0 °C for 4 h, concentrated sulfuric acid (2.5 mL) was slowly added at 0 °C followed by diethyl ether (100 mL). After decantation, the aqueous layer was extracted with diethyl ether (2 x 150 mL). The combined organic layers were dried over magnesium sulfate, filtered and evaporated under reduced pressure. The residue was purified by column chromatography (SiO_2, ethyl acetate/cyclohexane/trifluoroacetic acid, 20/80/0.1→50/50/0.1, v/v/v) to give compound **S22** as a light yellow oil (2.58 g, 8.57 mmol). Yield: 41%; R_f = 0.69 (SiO_2, ethyl acetate/cyclohexane/trifluoroacetic acid, 50/50/0.1, v/v/v); IR (ATR accessory) ν 3100-2900, 1750-1700, 1261, 1200-1100 cm^{-1}; ^{1}H NMR (200.13 MHz, $CDCl_3$) δ 2.2-2.55 (m, 2H), 2.51 (m, 2H), 4.30 (dd, 1H, *J* = 5.9, 8.3 Hz), 5.04 (s, 2H), 7.27 (m, 5H), 7.51 (brs, 1H); ^{13}C NMR (50.32 MHz, $CDCl_3$) δ 29.5, 31.5, 44.3, 66.9, 128.4 (2C), 128.5, 128.7 (2C), 135.6, 172.2, 174.5; HRMS *m/z* calculated for $C_{12}H_{14}BrO_4^+$ $[M+H]^+$: 301.0070, found: 301.0065.

5-Benzyl 1-*tert*-butyl 2-bromopentandioate (**S23**)

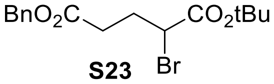

S23

This compound was obtained according to slight modifications of the procedure described by Lamarque, L. *et al*. [19]. To a solution of 2-bromopentandioic acid 5-benzyl ester (**S23**) (1.43 g, 4.75 mmol) in *tert*-butyl acetate (18 mL) was added dropwise an aqueous perchloric acid solution (70 wt%, 21 µL, 0.22 mmol). After stirring

at room temperature for 40 h, deionized water was added (25 mL) and the aqueous layer was extracted with *tert*-butyl acetate (2 x 5 mL). The combined organic layers were washed successively with deionized water (15 mL), 5% aqueous sodium hydrogen carbonate solution (15 mL) and deionized water (15 mL). The organic layer was dried over magnesium sulfate, filtered and evaporated under reduced pressure. The residue was purified by column chromatography (SiO_2, ethyl acetate/cyclohexane, 1/9, v/v) to give compound **S23** as a colourless oil (1.21 g, 3.39 mmol). Yield: 71%; R_f = 0.49 (SiO_2, ethyl acetate/cyclohexane, 1/9, v/v); IR (ATR accessory) ν 2979, 1731, 1368, 1257, 1139 cm^{-1}; ^1H NMR (200.13 MHz, $CDCl_3$) δ 1.47 (s, 9H), 2.20-2.50 (m, 2H), 2.56 (m, 2H), 4.25 (dd, 1H, *J* = 6.0, 8.2 Hz), 5.13 (s, 2H), 7.34 (m, 5H); ^{13}C NMR (50.32 MHz, $CDCl_3$) δ 27.8, 29.8 (3C), 31.7, 46.7, 66.6, 82.7, 128.4 (2C), 128.5, 128.7 (2C), 135.8, 168.4, 172.1; HRMS *m*/*z* calculated for $C_{16}H_{21}BrNaO_4^+$ $[M+Na]^+$: 379.0515, found: 379.0518.

Di-*tert*-butyl 2,2'-(1,4,7-triazacyclononane-1,4-diyl)diacetate (**S24**)

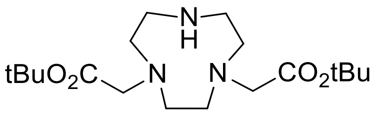

S24

This compound was obtained according to a modified procedure of the protocol described by Shetty, D. *et al*. [20]. To a solution of 1,4,7-triazacyclononane (**S19**) (1.60 g, 12.4 mmol) in anhydrous chloroform (35 mL) was added dropwise, over 4 h, a solution of *tert*-butyl 2-bromoacetate (4.0 mL, 27.1 mmol) in anhydrous chloroform (60 mL). The reaction mixture was stirred at room temperature until TLC monitoring (SiO_2, dichloromethane/ethanol/ammonia, 9/1/0.1, v/v/v) indicated no further advancement of the reaction (60 h). After filtration, the solid was washed with dichloromethane (2 x 10 mL) and the combined filtrates were evaporated under reduced pressure. The crude was taken up with deionized water (10 mL) and pH was adjusted to 3 by addition a 1 N hydrochloric acid solution. The resulting solution was washed with diethyl ether (4 x 25 mL) before adjusting carefully the pH to 8 with a 1 N aqueous sodium hydroxide solution. After extraction with dichloromethane (3 x 25 mL), the combined organic layers were dried over magnesium sulfate, filtered and evaporated under reduced pressure. The residue was taken up with hexane (5 mL) and stirred at 0 °C for 30 min. The solid was filtered to give compound **S24** as an off-white solid (1.94 g, 5.43 mmol), which was used in the next step without further purification. Yield: 44%; mp 154-156 °C; R_f = 0.20 (SiO_2, dichloromethane/ethanol/ammonia, 9/1/0.1, v/v/v); IR (ATR accessory) ν 3050-2900, 2800-2700, 1726, 1369, 1214, 1154 cm^{-1}; ^1H NMR (200.13 MHz, $CDCl_3$) δ 1.44 (s, 18H), 2.75 (s, 4H), 3.04 (t, 4H, *J* = 5.7 Hz), 3.21 (m, 4H), 3.35 (s, 4H); ^{13}C NMR (50.32 MHz, $CDCl_3$) δ 28.2 (6C), 44.7 (2C), 48.8 (2C), 51.8 (2C), 56.7

(2C), 82.1 (2C), 170.9 (2C); ESI-MS *m/z* calculated for $C_{18}H_{36}N_3O_4^+$ $[M+H]^+$: 358.27, found: 358.26.

1-Benzyl 5-*tert*-butyl 4-(4,7-bis(2-(*tert*-butoxy)-2-oxoethyl)-1,4,7-triazacyclononan-1-yl)pentanedioate (**S25**)

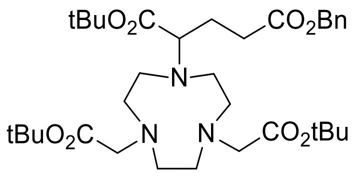

S25

To a solution of compound **S24** (600 mg, 1.68 mmol) in anhydrous acetonitrile (6 mL) were successively added, under argon, potassium carbonate (464 mg, 3.36 mmol) and a solution of 5-benzyl 1-*tert*-butyl 2-bromopentandioate (**S23**) (720 mg, 2.02 mmol) in anhydrous acetonitrile (1 mL). The reaction mixture was stirred at 40 °C for 24 h. After cooling to room temperature the solution was filtered and the solid was washed with acetonitrile (2 x 3 mL). The combined filtrates were evaporated under reduced pressure. The crude was purified by column chromatography (Al_2O_3, dichloromethane /ethanol, 99/1, v/v) to give compound **S25** as a yellow oil (731 mg, 1.15 mmol). Yield: 69%; R_f = 0.30 (Al_2O_3, dichloromethane/ethanol, 99/1, v/v); IR (ATR accessory) ν 2976, 2929, 1719, 1366, 1253, 1215, 1143 cm^{-1}; ^1H NMR (200.13 MHz, $CDCl_3$) δ 1.46 (s, 27H), 1.75-2.15 (m, 2H), 2.40-3.10 (m, 14H), 3.19 (dd, 1H, *J* = 6.4, 8.9 Hz), 3.29 (s, 4H), 5.14 (s, 2H), 7.36 (m, 5H); ^{13}C NMR (50.32 MHz, $CDCl_3$) δ 25.3, 28.3 (9C), 31.1, 53.3 (2C), 55.6 (2C), 56.2 (2C), 59.4 (2C), 66.3, 66.9, 80.7 (2C), 80.9, 128.3 (3C), 128.6 (2C), 136.1, 171.7 (2C), 172.6, 173.4; ESI-MS *m/z* calculated for $C_{34}H_{56}N_3O_8^+$ $[M+H]^+$: 634.41, found: 634.50.

4-(4,7-Bis(2-(*tert*-butoxy)-2-oxoethyl)-1,4,7-triazacyclononan-1-yl)-5-(*tert*-butoxy)-5-oxopentanoic acid (**S26**)

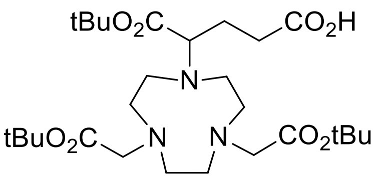

S26

To a degassed solution of benzyl derivative **S25** (493 mg, 0.78 mmol) in propan-2-ol (23 mL) was added Pd/C 10% (50 mg). After stirring at room temperature for 20 h under hydrogen atmosphere, the suspension was filtered over 0.45 µm PTFE membrane filter and the filtrate was evaporated under reduced pressure to give compound **S26** as a yellow oil (397 mg, 0.73 mmol). Yield: 94%; IR (ATR accessory)

ν 3000-2700, 1722, 1367, 1248, 1147 cm^{-1}; ^{1}H NMR (200.13 MHz, $CDCl_3$) δ 1.42 (s, 27H), 1.93 (m, 2H), 2.40 (m, 2H), 2.75-3.15 (m, 12H), 3.20-3.50 (m, 5H), 10.32 (brs, 1H); ^{13}C NMR (50.32 MHz, $CDCl_3$) δ 25.4, 28.0 (9C), 32.8, 51.3 (2C), 53.2 (2C), 54.1 (2C), 58.1 (2C), 66.7, 81.3 (3C), 170.1 (2C), 171.7, 176.9; ESI-MS *m/z* calculated for $C_{27}H_{50}N_3O_8^+$ [M+H]$^+$: 544.36, found: 544.41.

Di-*tert*-butyl 2,2'-(7-(2,2,15,15-tetramethyl-4,9,13-trioxo-3,14-dioxa-5,8-diazahexadecan-12-yl)-1,4,7-triazacyclononane-1,4-diyl)diacetate (**12**)

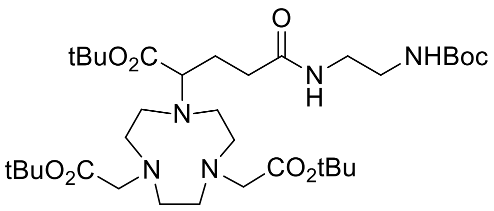

12

To a solution of acid **S26** (397 mg, 0.73 mmol) in anhydrous dichloromethane (18 mL) were successively added, under argon, 1-hydroxybenzotriazole monohydrate (128 mg, 0.95 mmol), *N*-ethyl-*N'*-(3-dimethylaminopropyl)carbodiimide hydrochloride salt (182 mg, 0.95 mmol) and a solution of *tert*-butyl *N*-(2-aminoethyl)carbamate [12] (152 mg, 0.95 mmol) in anhydrous dichloromethane (10 mL). After stirring at room temperature for 15 h, the reaction mixture was washed successively with a 1 N aqueous sodium hydroxide solution (2 x 25 mL) and brine (2 x 25 mL), dried over magnesium sulfate, filtered and evaporated under reduced pressure. The residue was purified by column chromatography (SiO_2, dichloromethane/ethanol/ammonia, 90/10/0.2, v/v/v) to give compound **12** as a colourless oil (336 mg, 0.49 mmol). Yield: 67%; R_f = 0.15 (SiO_2, dichloromethane/ethanol/ammonia, 90/10/0.2, v/v/v); IR (ATR accessory) ν 3450-3150, 2976, 2932, 1715, 1519, 1455, 1392, 1366, 1249, 1147 cm^{-1}; ^{1}H NMR (500.13 MHz, $CDCl_3$) δ 1.30-1.45 (m, 36H), 1.77 (m, 1H), 2.00 (m, 1H), 2.29 (m, 1H), 2.42 (m, 1H), 2.55-2.95 (m, 12H), 3.05-3.35 (m, 9H), 5.34 (m, 1H), 6.86 (m, 1H); ^{13}C NMR (50.32 MHz, $CDCl_3$) δ 26.3, 28.3 (9C), 28.5 (3C), 33.3, 40.0, 41.0, 53.7 (2C), 55.9 (4C), 59.7 (2C), 66.9, 79.3, 81.2 (3C), 156.5, 171.5 (2C), 172.8, 173.9; HRMS *m/z* calculated for $C_{34}H_{64}N_5O_9^+$ [M+H]$^+$: 686.4699, found: 686.4702.

1.6. Synthesis of N_3-PEG$_4$-NHS derivative S32

Scheme S4[a]: Synthesis of N_3-PEG$_4$-NHS derivative **S32**

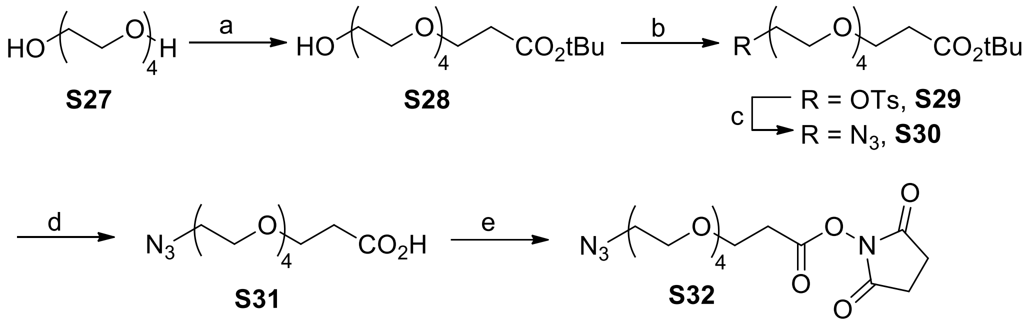

[a]Reaction conditions: a) (i) NaH, THF, RT, 10 min; (ii) *tert*-butyl acrylate, THF, RT, 20 h; b) TsCl, pyridine, 0 °C, 2 h then 4 °C, 60 h; c) NaN_3, DMF, RT then 55 °C, 1.5 h; d) HCl 4M in 1,4-dioxane, RT, 19 h; e) NHS, EDC.HCl, CH_2Cl_2, RT, 5.5 h.

tert-Butyl 1-hydroxy-3,6,9,12-tetraoxapentadecan-15-oate (**S28**)

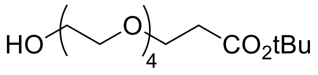

S28

Tetraethylene glycol (**S27**) (80.0 g, 0.41 mol) was dried by co-evaporation of anhydrous toluene (2 x 20 mL) and dissolved under argon in anhydrous tetrahydrofuran (200 mL). Sodium hydride 60% in mineral oil (290 mg, 7.25 mmol) was then added portionwise. After gas evolution stopped (10 min), a solution of *tert*-butyl acrylate (20.1 mL, 138 mmol) in anhydrous tetrahydrofuran (60 mL) was added dropwise over 1 h. After stirring at room temperature for 20 h, the reaction mixture was neutralized by addition of acetic acid (140 µL) and deionized water (690 µL). After stirring at room temperature for 30 min, brine (150 mL) was added and the resulting solution was extracted with ethyl acetate (3 x 150 mL). The combined organic layers were washed with brine (3 x 150 mL), dried over magnesium sulfate, filtered and evaporated under reduced pressure to give ester **S28** as a colourless oil (32.05 g, 99.4 mmol). Yield: 73%; IR (ATR accessory) ν 3700-3300, 3100-2700, 1727, 1366, 1157, 1100 cm^{-1}; ^{1}H NMR (200.13 MHz, $CDCl_3$) δ 1.40 (s, 9H), 2.46 (t, 2H, J = 6.5 Hz), 2.98 (brs, 1H), 3.50-3.70 (m, 18H); ^{13}C NMR (50.32 MHz, $CDCl_3$) δ 28.1 (3C), 36.2, 61.7, 66.9, 70.4, 70.6 (5C), 72.6, 80.6, 171.0; HRMS *m/z* calculated for $C_{15}H_{30}NaO_7^+$ $[M+Na]^+$: 345.1884, found: 345.1885.

tert-Butyl 1-tosyloxy-3,6,9,12-tetraoxapentadecan-15-oate (**S29**)

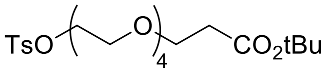

S29

To a solution of alcohol **S28** (17.78 g, 55.2 mmol) in anhydrous pyridine (23 mL) was added portionwise under argon and at 0 °C, tosyl chloride (11.60 g, 60.8 mmol). The reaction was stirred at 0 °C for 2 h then at 4 °C for 60 h. The mixture was poured into ice/water (300 mL) and extracted with dichloromethane (4 x 100 mL). The combined organic layers were washed successively with a 1 N hydrochloric acid solution (2 x 100 mL) and brine (100 mL), dried over magnesium sulfate, filtered and evaporated under reduced pressure. The residue was purified by column chromatography (SiO_2, ethyl acetate/cyclohexane, 8/2, v/v) to give compound **S29** as a colourless oil (24.06 g, 50.5 mmol). Yield: 91%; R_f = 0.60 (SiO_2, ethyl acetate/cyclohexane, 8/2, v/v); IR (ATR accessory) ν 3050-2850, 1732, 1352, 1174, 1095, 917 cm^{-1}; ^1H NMR (200.13 MHz, $CDCl_3$) δ 1.40 (s, 9H), 2.41 (s, 3H), 2.45 (t, 2H, *J* = 6.5 Hz), 3.50-3.60 (m, 14H), 3.66 (t, 2H, *J* = 6.5 Hz), 4.12 (t, 2H, *J* = 4.7 Hz), 7.31 (d, 2H, *J* = 8.3 Hz), 7.75 (d, 2H, *J* = 8.3 Hz); ^{13}C NMR (50.32 MHz, $CDCl_3$) δ 21.5, 27.9 (3C), 36.1, 66.7, 68.4, 69.2, 70.2, 70.3 (2C), 70.4 (2C), 70.5, 80.2, 127.8 (2C), 129.7 (2C), 132.8, 144.7, 170.7; HRMS *m/z* calculated for $C_{22}H_{36}NaO_9S^+$ $[M+Na]^+$: 499.1972, found: 499.1971.

tert-Butyl 1-azido-3,6,9,12-tetraoxapentadecan-15-oate (**S30**)

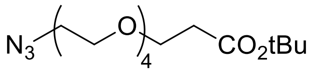

S30

To a solution of tosylated derivative **S29** (35.20 g, 73.9 mmol) in anhydrous *N*,*N*-dimethylformamide (110 mL) was added under argon sodium azide (16.81 g, 259 mmol). The reaction mixture was stirred at room temperature for 26 h and then at 55 °C for 1.5 h. After cooling to room temperature, the reaction mixture was diluted with deionised water (650 mL) and extracted with ethyl acetate (3 x 250 mL). The combined organic layers were washed successively with deionised water (3 x 250 mL) and brine (300 mL), dried over magnesium sulfate, filtered and evaporated under reduced pressure to give compound **S30** as a pale yellow oil (25.52 g, 73.5 mmol), which was engaged in the next step without further purification. Yield: 99%; IR (ATR accessory) ν 2950-2800, 2101, 1727, 1366, 1281, 1253, 1106 cm^{-1}; ^1H NMR (200.13 MHz, $CDCl_3$) δ 1.38 (s, 9H), 2.43 (t, 2H, *J* = 6.5 Hz), 3.32 (t, 2H, *J* = 5.0 Hz), 3.50-3.75 (m, 16H); ^{13}C NMR (50.32 MHz, $CDCl_3$) δ 28.0 (3C), 36.2, 50.6, 66.8, 70.0, 70.3, 70.4, 70.6 (4C), 80.4, 170.8; ESI-MS *m/z* calculated for $C_{15}H_{29}N_3NaO_6^+$ $[M+Na]^+$: 370.20, found: 370.21.

tert-Butyl 1-azido-3,6,9,12-tetraoxapentadecan-15-oic acid (**S31**)

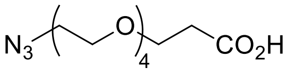

S31

Compound **S30** (418 mg, 1.20 mmol) was dissolved under argon in a 4 M solution of hydrogen chloride in anhydrous 1,4-dioxane (4 mL). After stirring at room temperature for 19 h, the reaction mixture was evaporated under reduced pressure (bath temperature < 40 °C) to give acid **S31** as a yellow oil (338 mg, 1.16 mmol), which was used in the next step without further purification. Yield: 96%; R_f = 0.16 (SiO_2, ethyl acetate); IR (ATR accessory) ν 2950-2800, 2105, 1724, 1350, 1173, 1095 cm^{-1}; ^{1}H NMR (500.13 MHz, $CDCl_3$) δ 2.62 (t, 2H, *J* = 6.3 Hz), 3.38 (t, 2H, *J* = 5.2 Hz), 3.58-3.70 (m, 14H), 3.75 (t, 2H, *J* = 6.3 Hz), 9.14 (brs, 1H); ^{13}C NMR (125.76 MHz, $CDCl_3$) δ 35.0, 50.8, 66.5, 70.1, 70.5, 70.6, 70.7, 70.8, 76.9, 77.2, 176.1; ESI-MS *m/z* calculated for $C_{11}H_{21}N_3NaO_6^+$ $[M+Na]^+$: 314.13, found: 314.14.

2,5-Dioxopyrrolidin-1-yl 1-azido-3,6,9,12-tetraoxapentadecan-15-oate (**S32**)

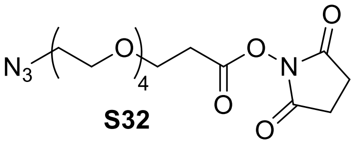

S32

To a solution of acid **S31** (338 mg, 1.16 mmol) in anhydrous dichloromethane (8 mL) were successively added, under argon, *N*-hydroxysuccinimide (200 mg, 1.74 mmol) and *N*-ethyl-*N'*-(3-dimethylaminopropyl)carbodiimide hydrochloride salt (334 mg, 1.74 mmol). The reaction mixture was stirred at room temperature for 5.5 h. After evaporation under reduced pressure, the residue was purified by column chromatography (SiO_2, ethyl acetate) to give activated ester **S32** as a colourless oil (290 mg, 747 μmol). Yield: 64%; R_f = 0.59 (SiO_2, ethyl acetate); IR (ATR accessory) ν 3000-2800, 2102, 1734, 1203, 1150-1000 cm^{-1}; ^{1}H NMR (500.13 MHz, $CDCl_3$) δ 2.76 (brs, 4H), 2.83 (t, 2H, *J* = 6.4 Hz), 3.32 (t, 2H, *J* = 5.2 Hz), 3.56-3.63 (m, 14H), 3.78 (t, 2H, *J* = 6.4 Hz); ^{13}C NMR (50.32 MHz, $CDCl_3$) δ 25.5 (2C), 32.1, 50.6, 65.6, 69.9, 70.4, 70.5 (2C), 70.6 (3C), 166.7, 169.1 (2C); ESI-MS *m/z* calculated for $C_{15}H_{24}N_4NaO_8^+$ $[M+Na]^+$: 411.15, found: 411.15.

2. ^{125}I-labelling of trastuzumab and immunoreactive fraction (IRF) determination

2.1. General

[^{125}I]NaI (3.60-3.63 GBq/mL, 643.8 MBq/mg) was purchased from PerkinElmer Life and Analytical Sciences (331 Treble Cove Road, Billerica, MA 01862, US) as a no-carrier-added solution in reductant free 1.0 × 10^{-5} M aqueous sodium hydroxide solution (pH 8-11). Pierce iodination tubes were purchased from Thermo Fischer Scientific (28601). Radio-instant thin-layer chromatography (radio-ITLC) analyses were measured on a miniGITA Dual radio-TLC system (Elysia-Raytest) using silica gel-impregnated chromatography paper (Varian inc.) eluted with PBS. The anti-HER2 antibody, Trazimera a biosimilar of trastuzumab (also called trastuzumab-qyyp, PF-05280014) was provided by the Cancer Center Jean Perrin, Clermont-Ferrand, France.

2.2. Radiolabelling of trastuzumab with iodine-125

Native trastuzumab was radiolabelled *via* direct electrophilic radioiodination on tyrosine residues using Pierce tubes pre-coated with iodogen, a mild oxidative reagent. Typically, a solution of antibody (400 µg) in PBS (200-250 µL) was introduced in the Pierce tube before addition of carrier-free [^{125}I]NaI (18-19 µL, 36.6-38.5 MBq). The Pierce tube was sealed and maintained under gentle agitation at room temperature. After 20 min, the reaction media was transferred to a glass vial. The final radiochemical purity (RCP) of the radiotracer was determined by radio-ITLC analyses (see Figure S1). RCY: 68 ± 7%; RCP: 96.3 ± 2.8%; Molar activity: 13.5 ± 1.2 GBq/µmol (n = 3).

2.3. IRF determination

The immunoreactivity of [^{125}I]I-trastuzumab was determined by a conventional saturation assay according to the method recommended by Denoël *et al.* [21]. Increasing concentrations of BT474 cells (1.10^6, 2.10^6, 5.10^6 and 10.10^6 cells per tube) were incubated in 0.5 mL of binding media (25 mM HEPES pH 7, completed with Dulbecco's Modified Eagle's Medium (DMEM) F12 (1/1, v/v) glutamax) with 0.65 pmol of [^{125}I]I-trastuzumab (31 GBq/µmol) in a final volume of 1 mL. After 30 min of gentle shaking, samples were centrifuged at 460 g for 8 min at 4 °C. The supernatants were removed, the cell pellets were washed with PBS containing 0.2% of BSA and centrifuged again at 460 g for 8 min. The collected supernatants and pellets were separately recovered for radioactivity counting using a γ-counter (Wallac 1480 Wizard 3'', Perkin Elmer). The IRF was determined by performing a rectangular hyperbolic fit (one site specific binding, GraphPad Prism 9.4.1) of the binding curve obtained by plotting B/(B+S) as a function of cell concentration where B and S are the activities

counted in pellets and supernatants, respectively. IRF was obtained from the extrapolation of the quadratic hyperbola value at infinite antigen concentration. Experiment was performed in triplicates.

3. Supplementary figures

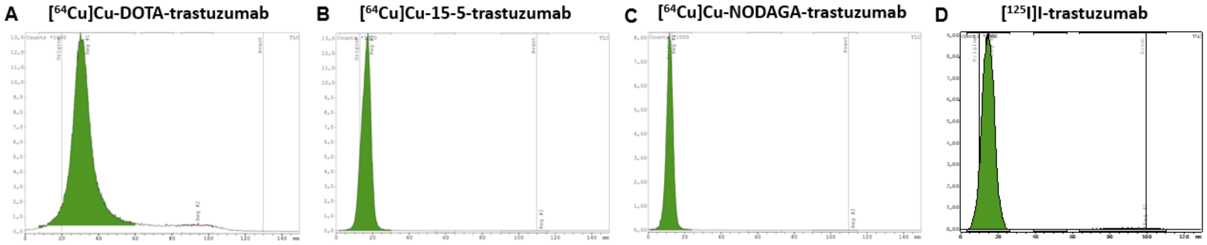

Figure S1. Radiochemical purity of [^{64}Cu]Cu-DOTA-trastuzumab (**A**), [^{64}Cu]Cu-15-5-trastuzumab (**B**)**,** [^{64}Cu]Cu-NODAGA-trastuzumab (**C**) and [^{125}I]I-trastuzumab (**D**) assessed by radio-ITLC chromatograms (eluent: PBS with 1 mM EDTA for ^{64}Cu-labelled conjugates). The radiolabelled immunoconjugates remained at the origin (R_f = 0) while unbound $^{64}Cu^{2+}$ migrated near the solvent front (R_f = 0.9-1).

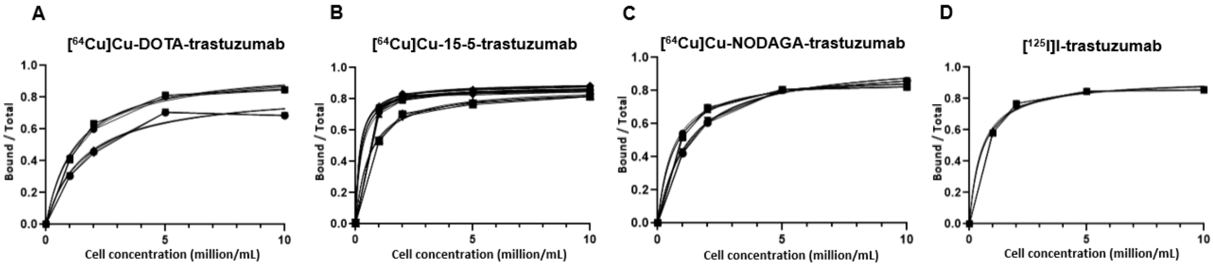

Figure S2. Binding assays plots used to determine the IRF of [^{64}Cu]Cu-DOTA-trastuzumab (**A**), [^{64}Cu]Cu-15-5-trastuzumab (**B**), [^{64}Cu]Cu-NODAGA-trastuzumab (**C**) and [^{125}I]I-trastuzumab (**D**) in HER2-expressing BT474 cell line by nonlinear regression analyses (one site specific binding) on GraphPad Prism 9.4.1. Each curve represents one replicate from one to four experiments performed in triplicate.

4. ^{1}H and ^{13}C NMR spectra of all synthesised compounds

^{1}H (top) and ^{13}C (down) NMR spectra of compound **S2** in $CDCl_3$

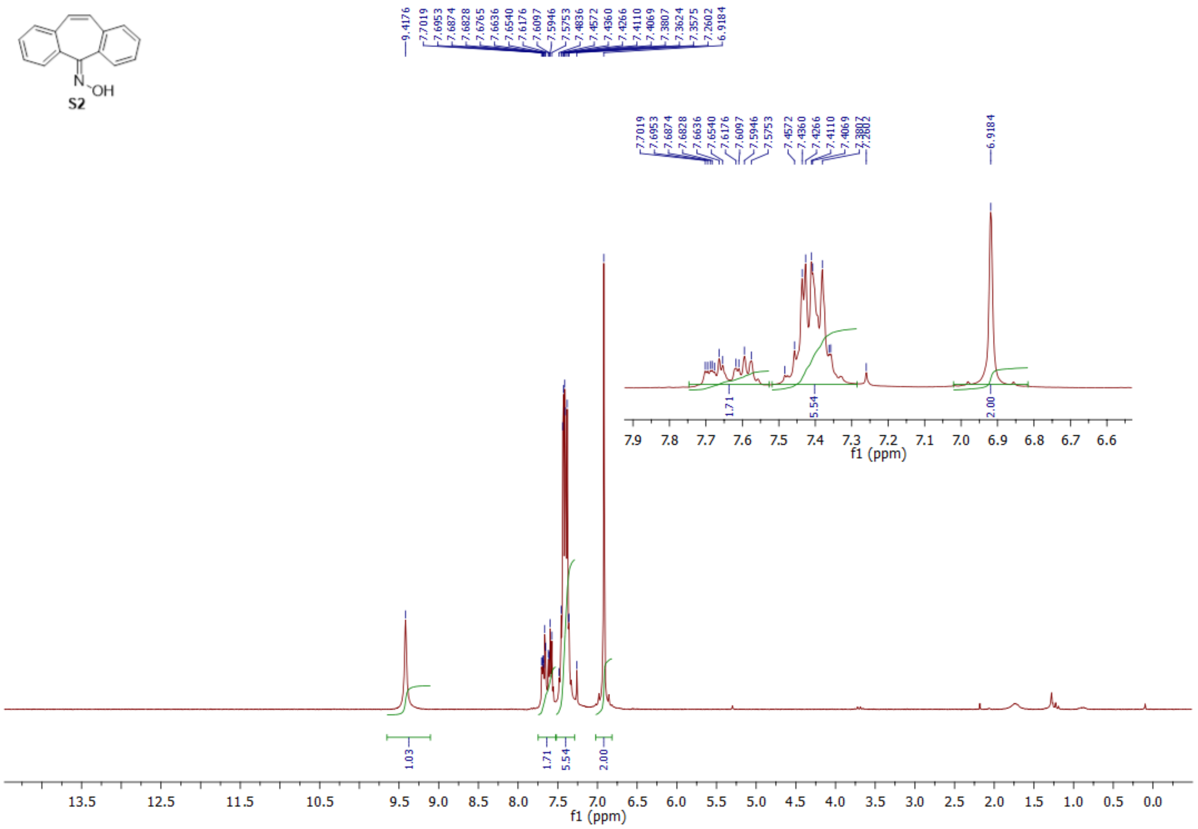

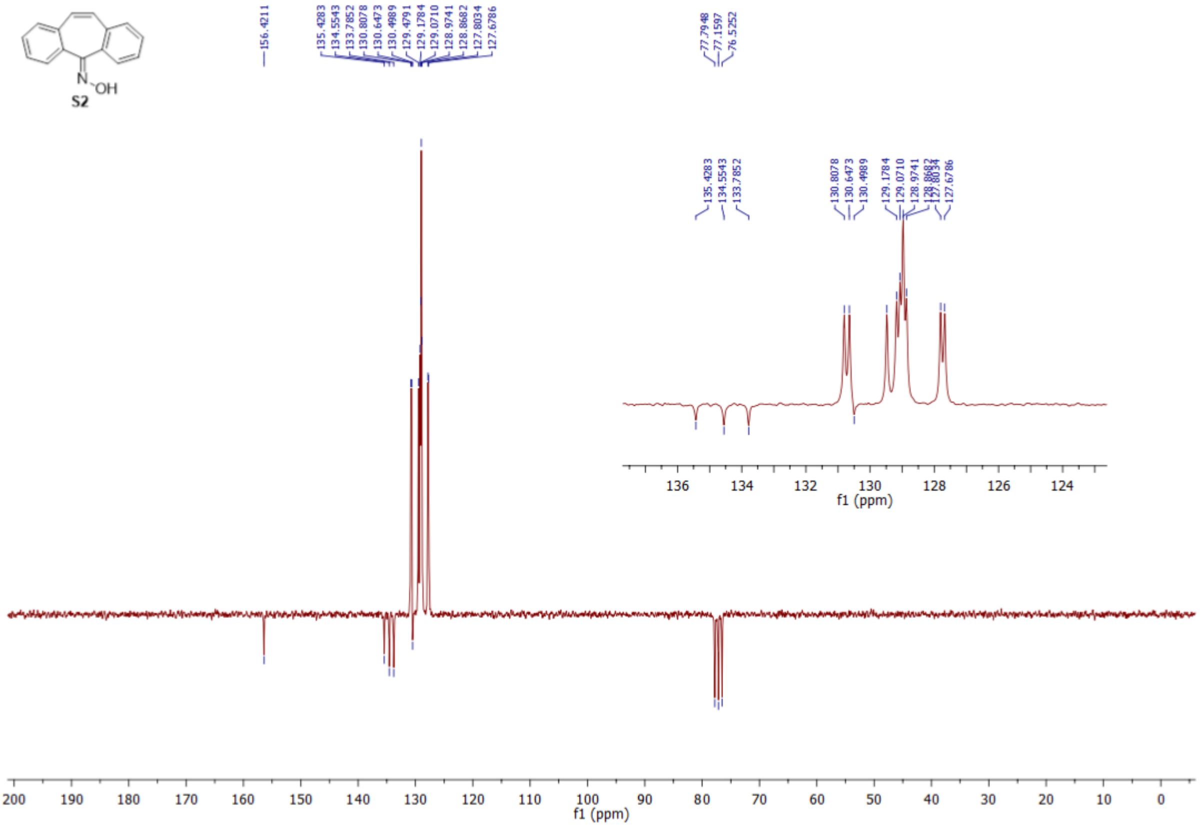

^1H (top) and ^{13}C (down) NMR spectra of compound **S3** in DMSO-d$_6$

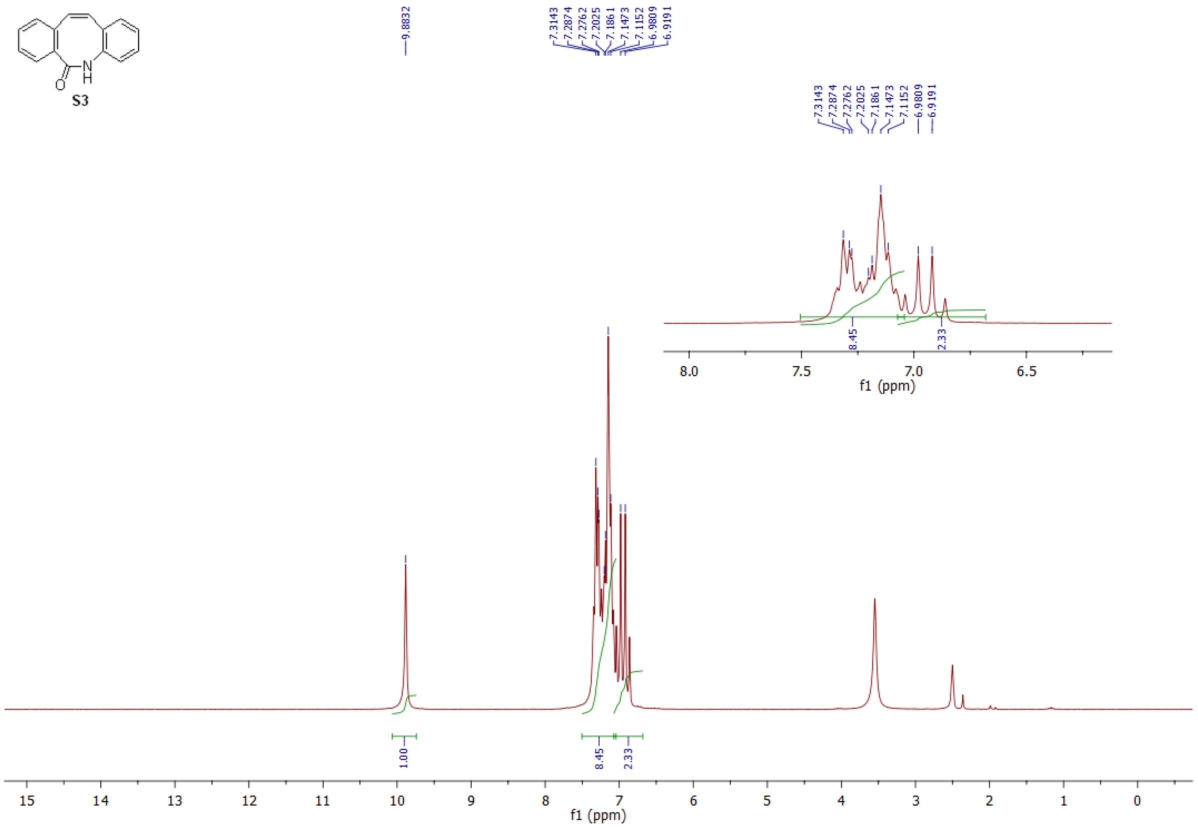

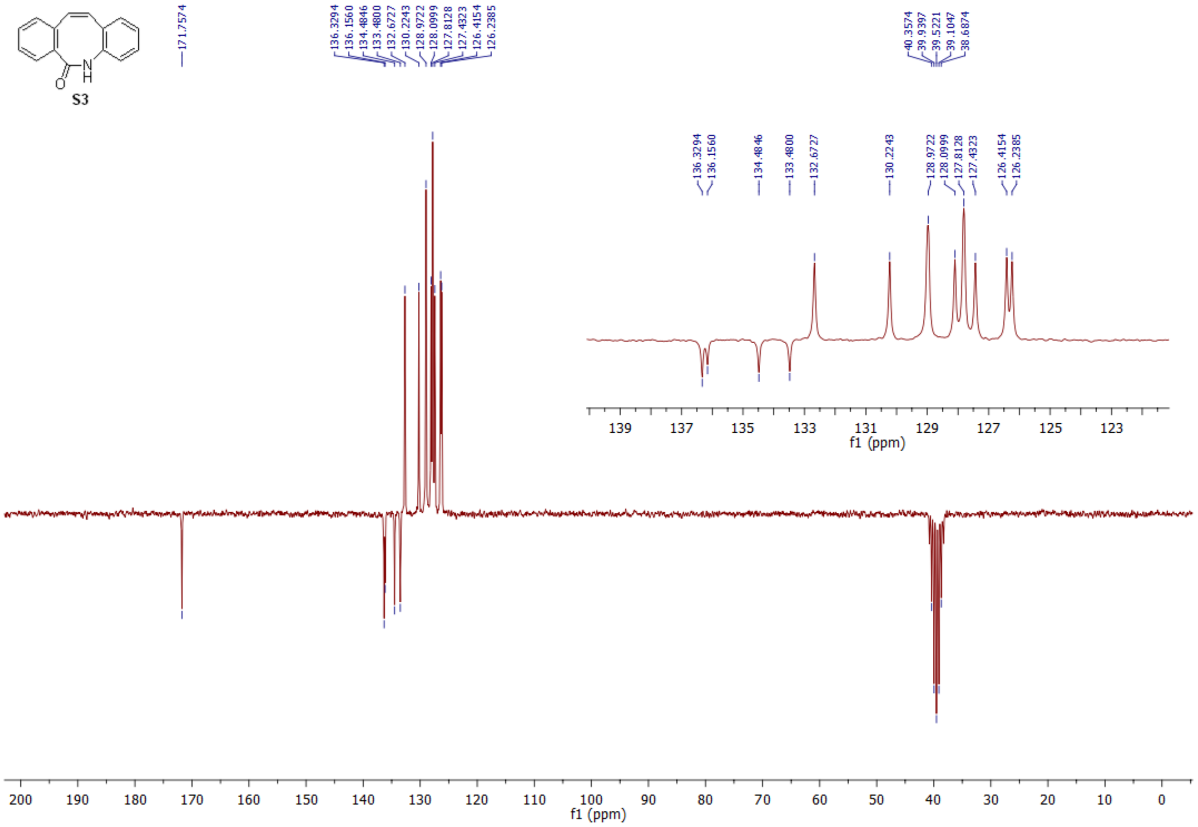

^{1}H (top) and ^{13}C (down) NMR spectra of compound **S4** in $CDCl_3$

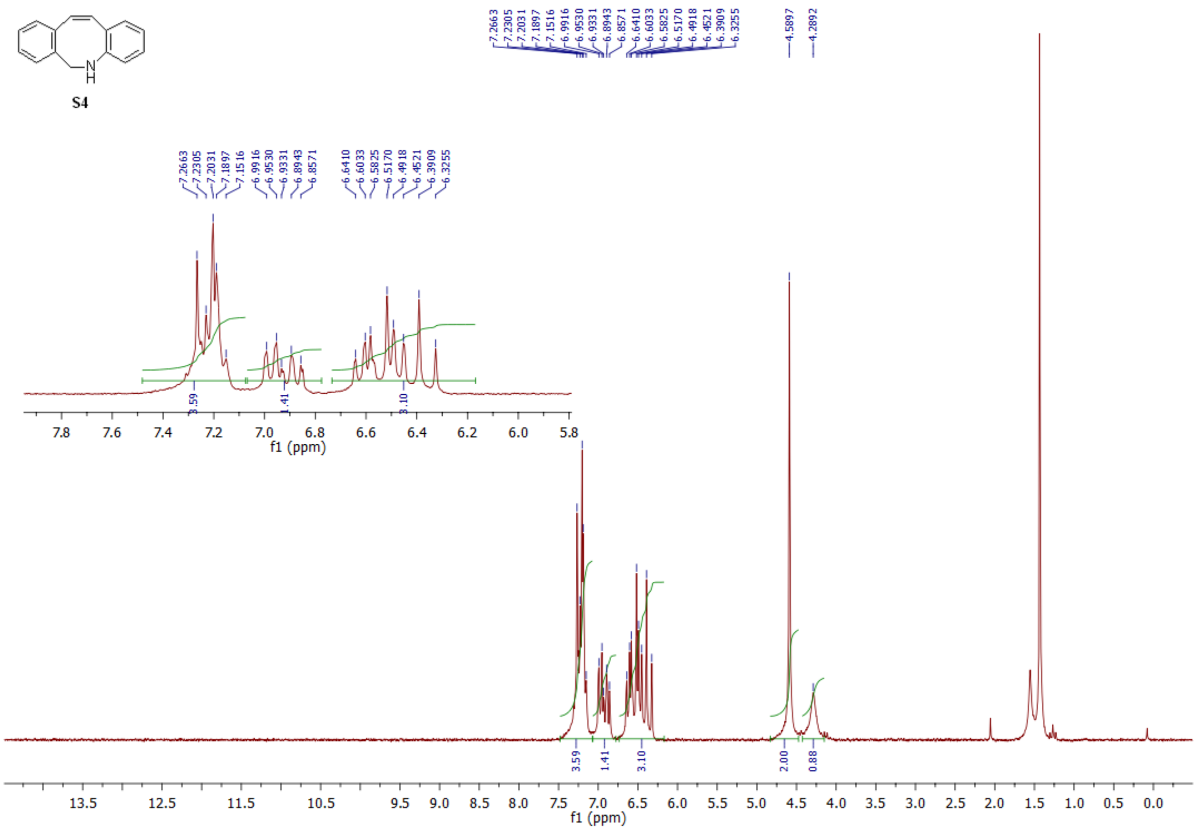

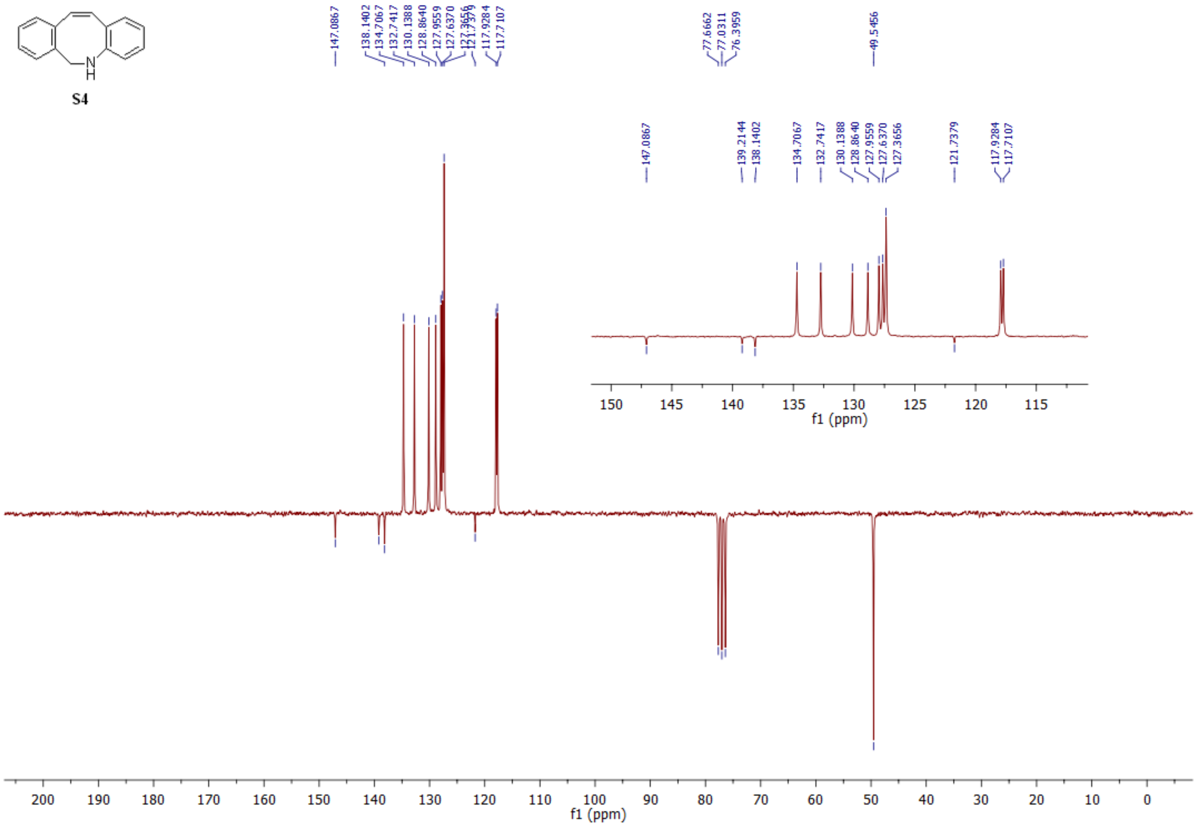

^{1}H (top) and ^{13}C (down) NMR spectra of compound **S6** in $CDCl_3$

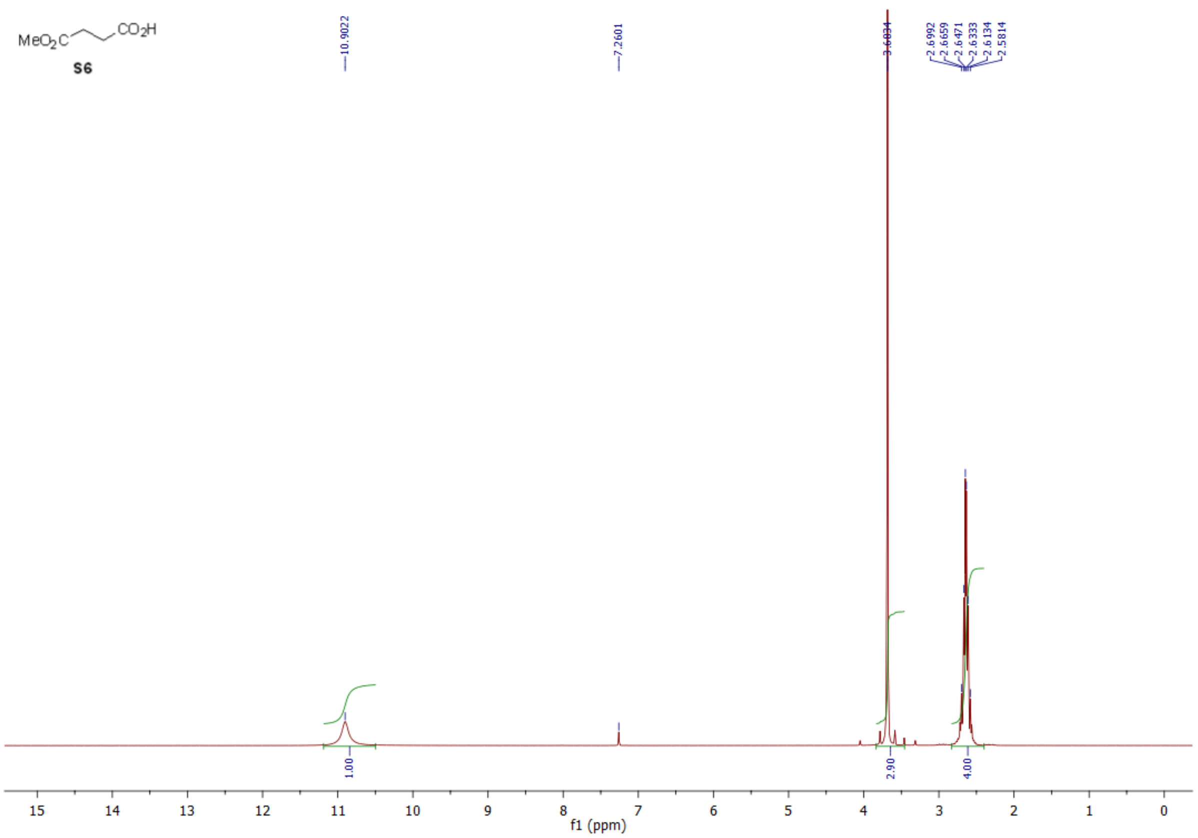

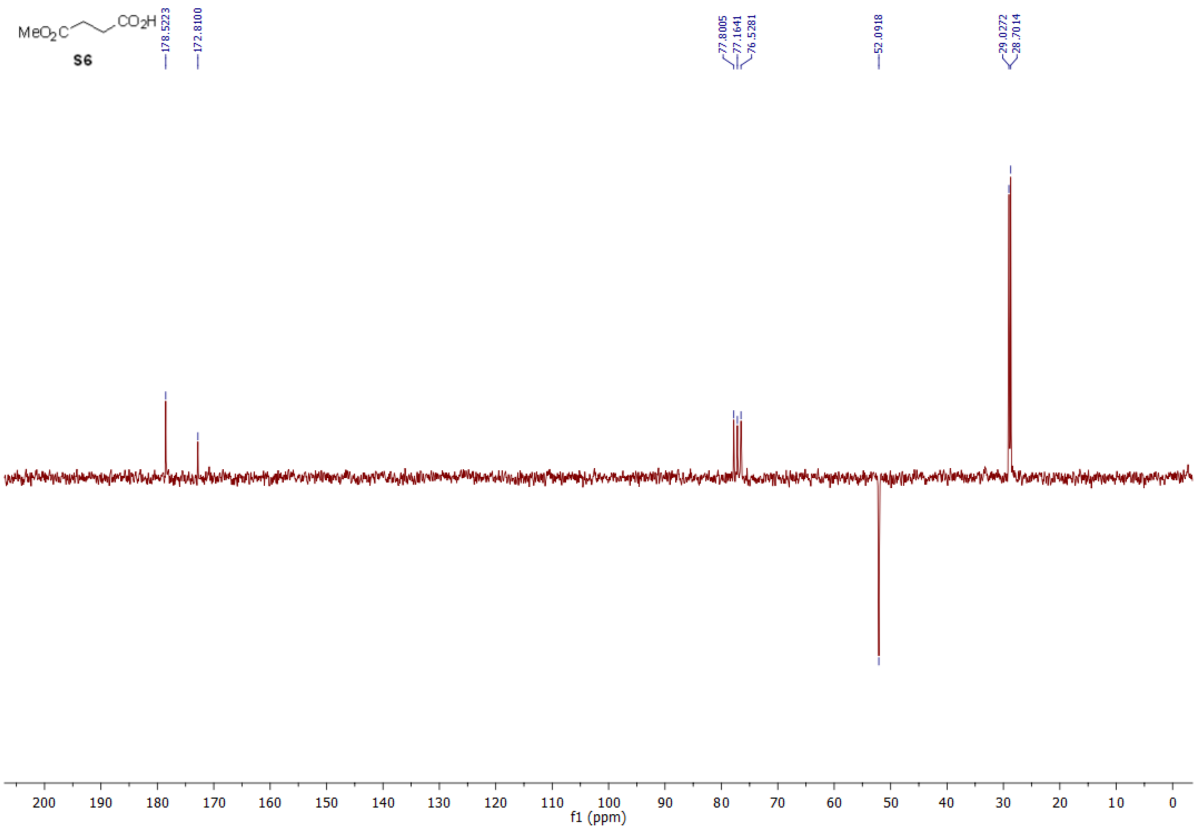

^{1}H (top) and ^{13}C (down) NMR spectra of compound **S7** in $CDCl_3$

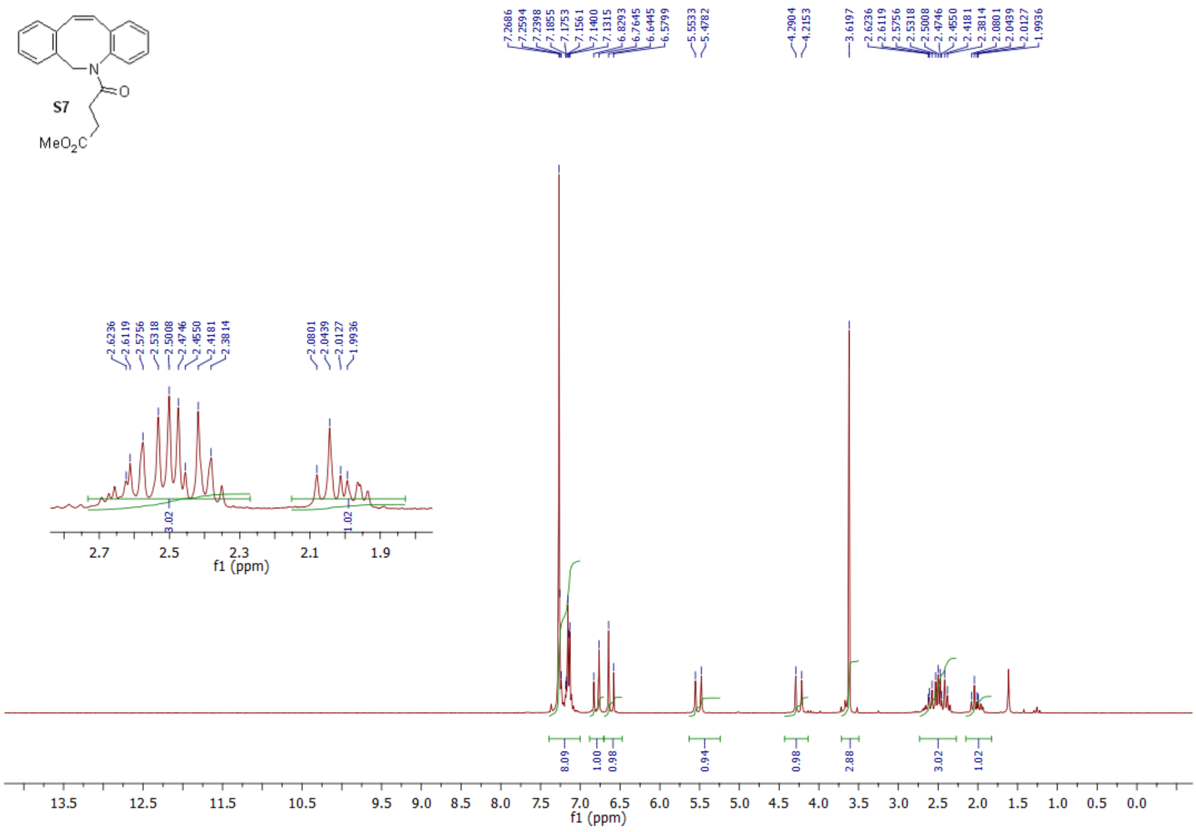

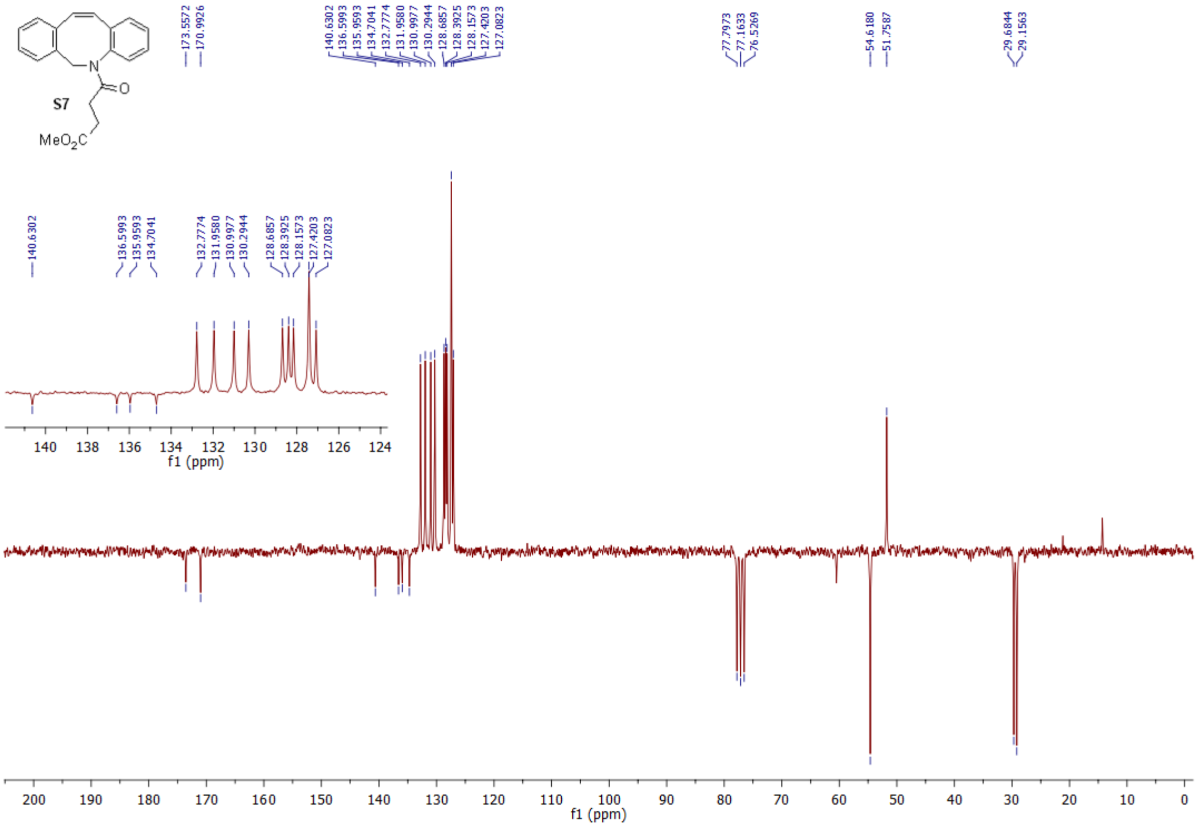

^{1}H (top) and ^{13}C (down) NMR spectra of compound **S8** in $CDCl_3$

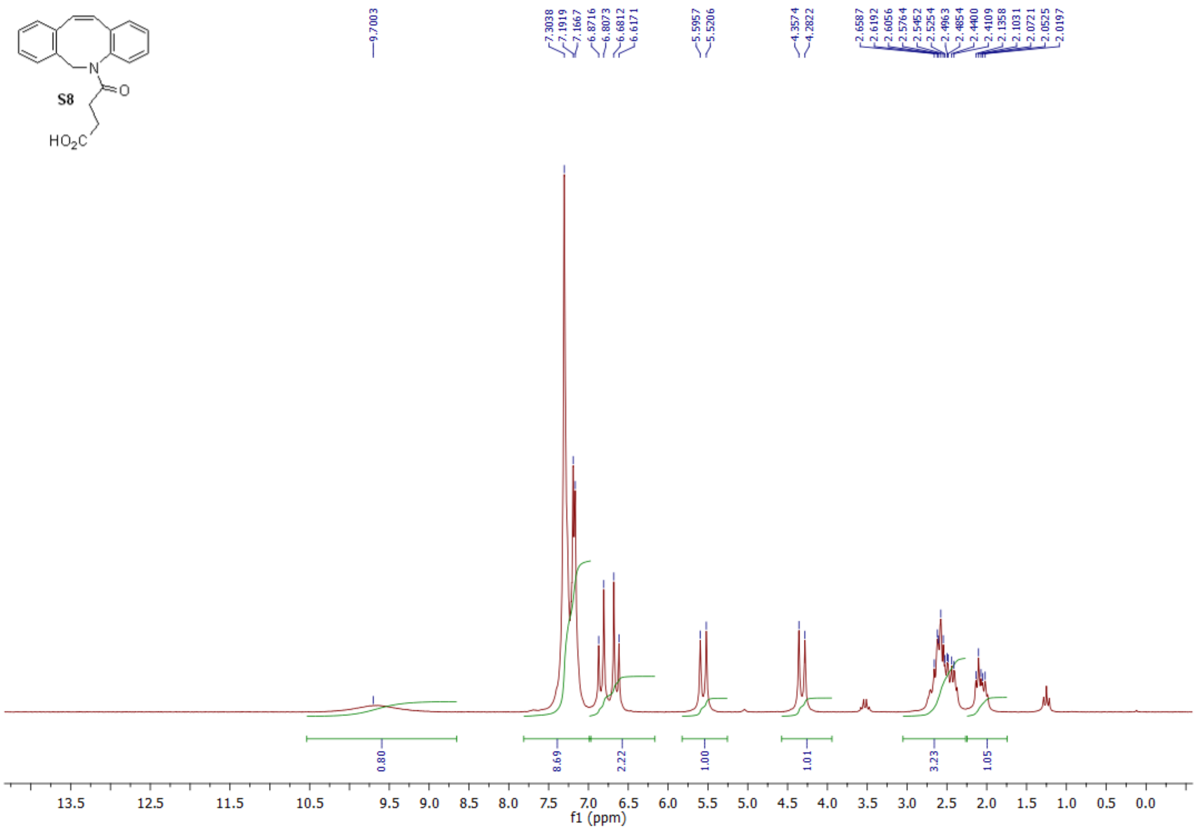

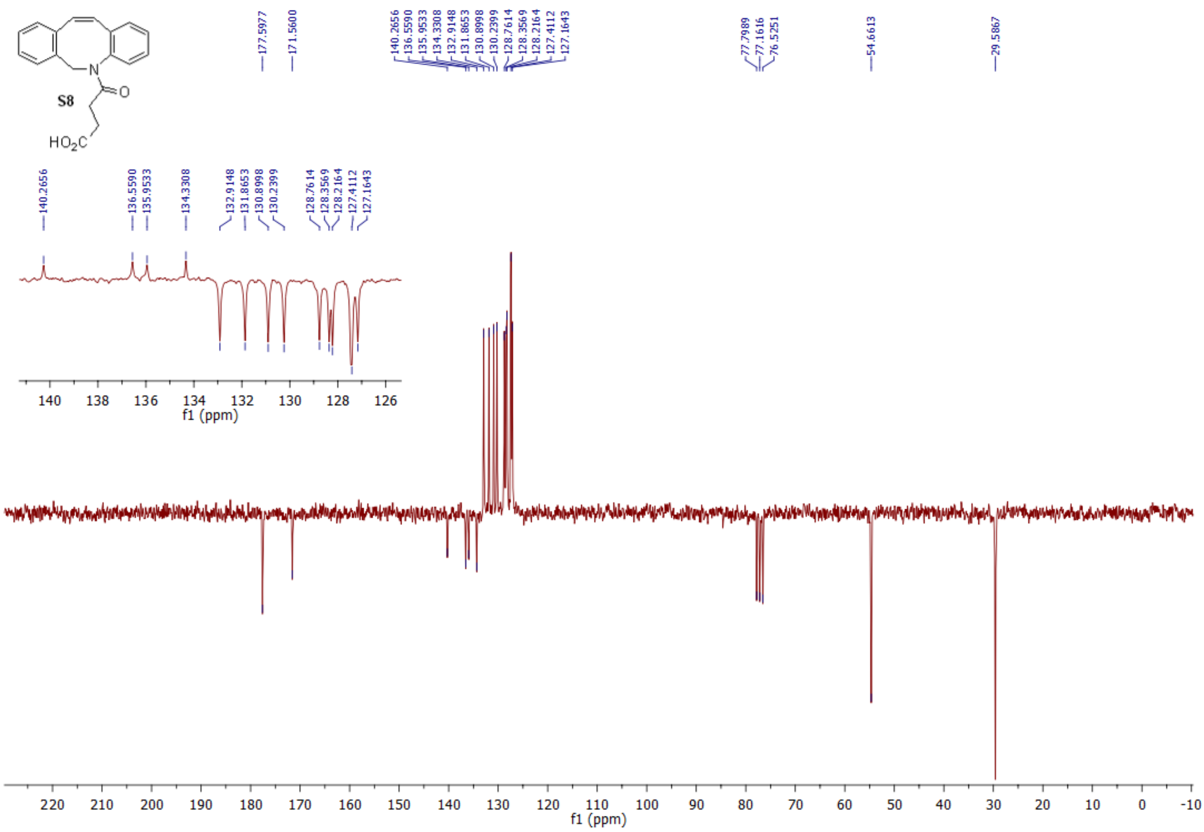

^{1}H (top) and ^{13}C (down) NMR spectra of compound **S9** in DMSO-d_6

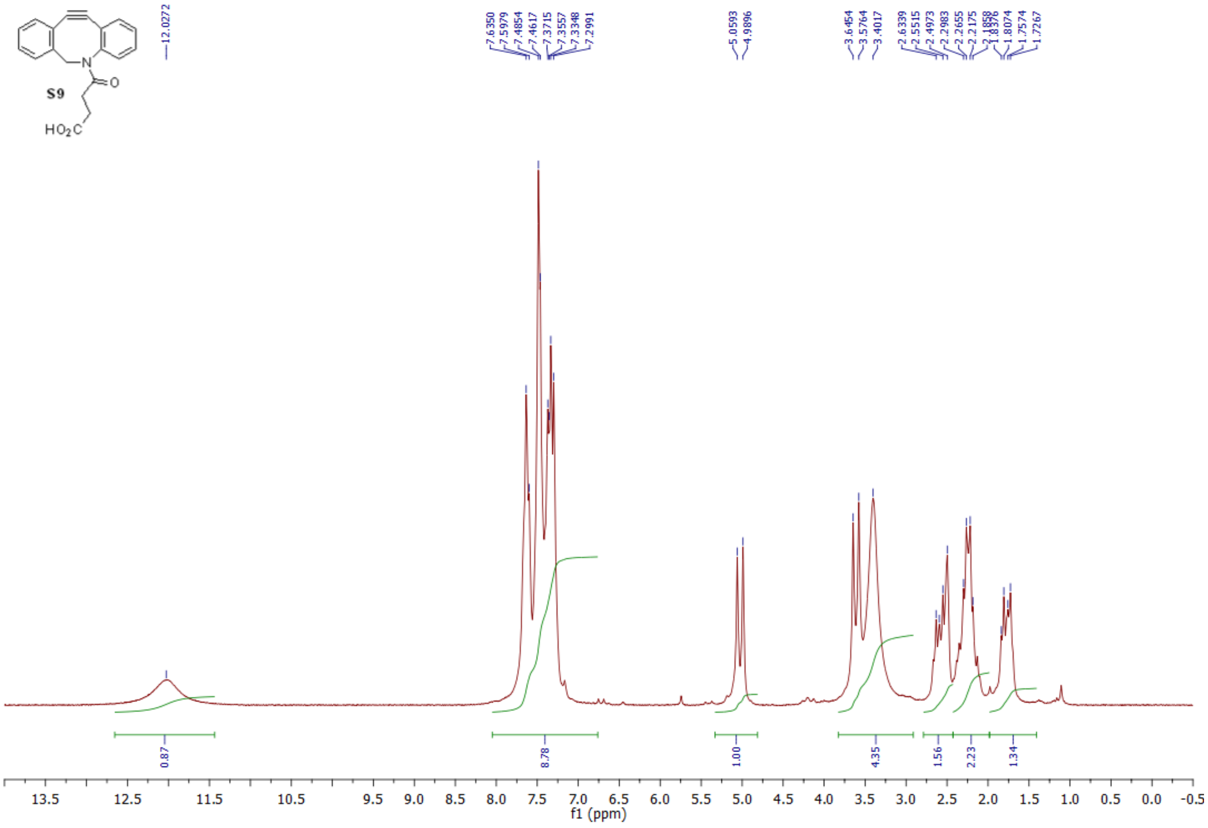

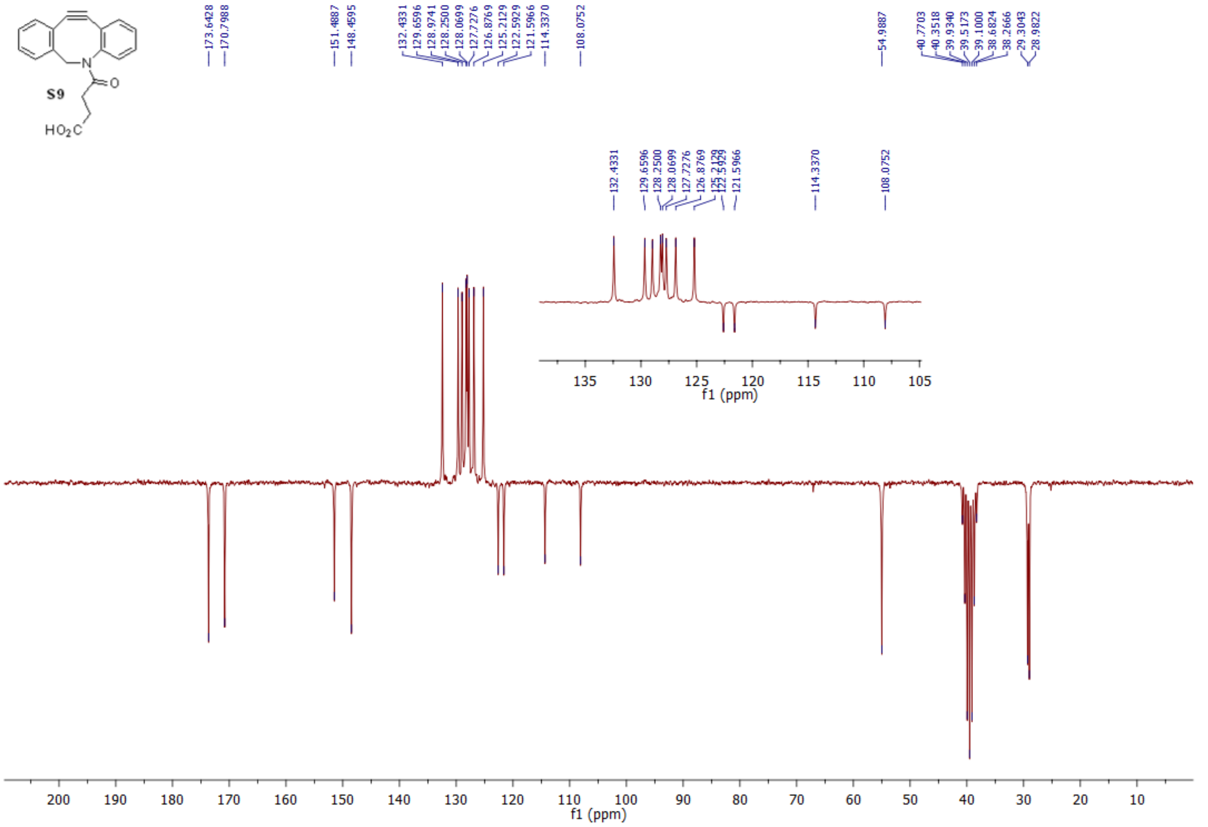

^{1}H (top) and ^{13}C (down) NMR spectra of compound **S10** in $CDCl_3$

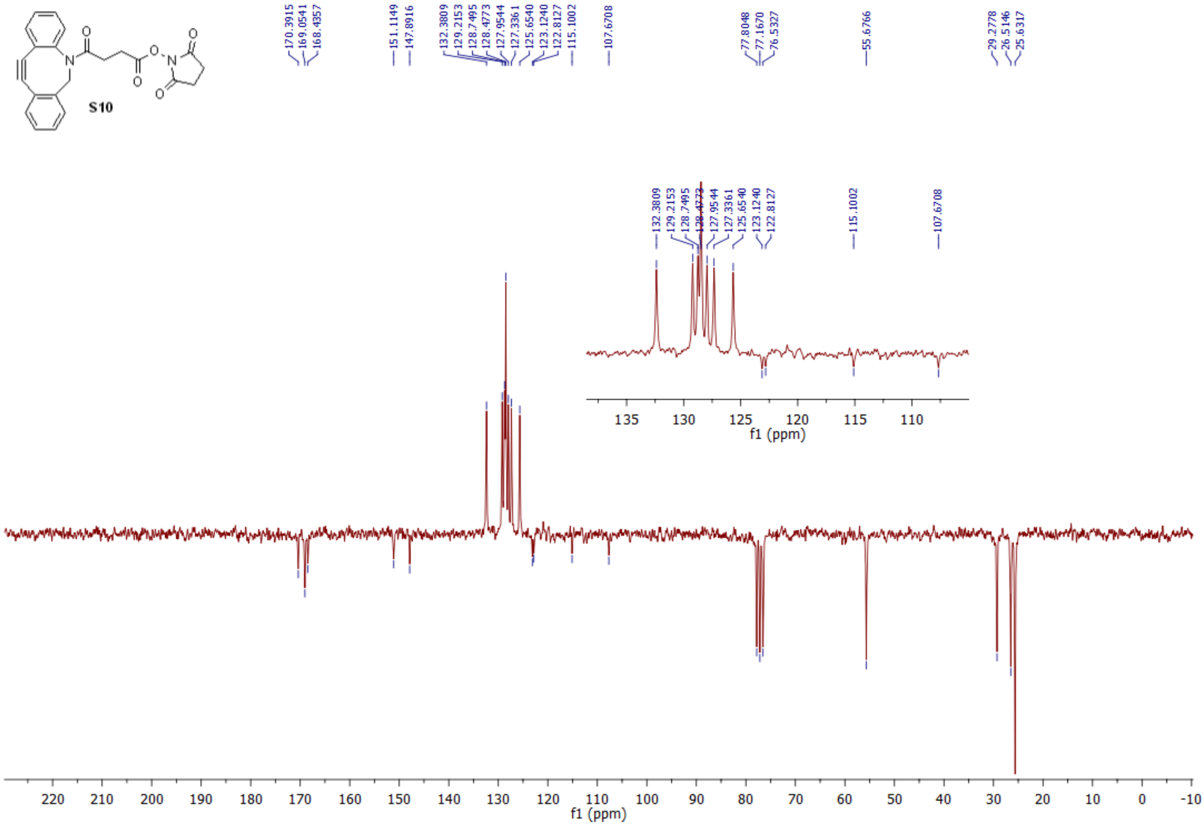

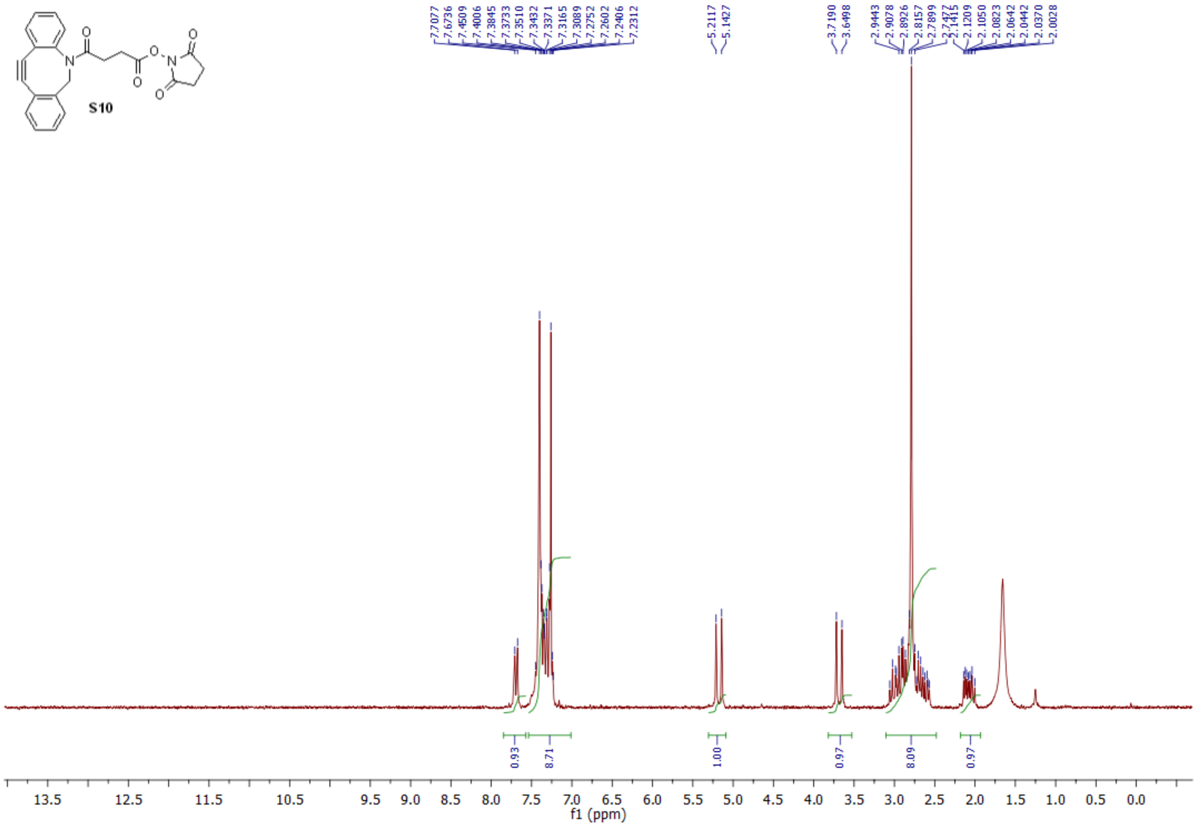

^{1}H (top) and ^{13}C (down) NMR spectra of compound **S11** in $CDCl_3$

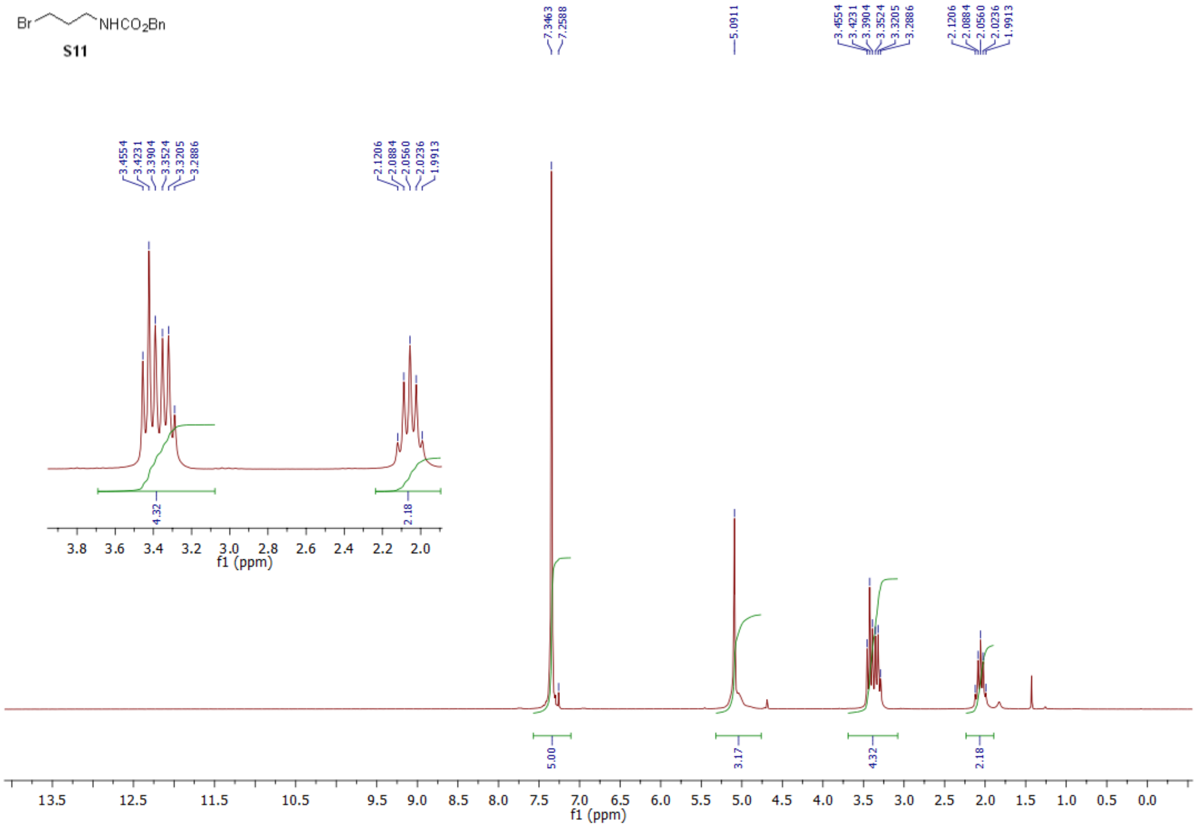

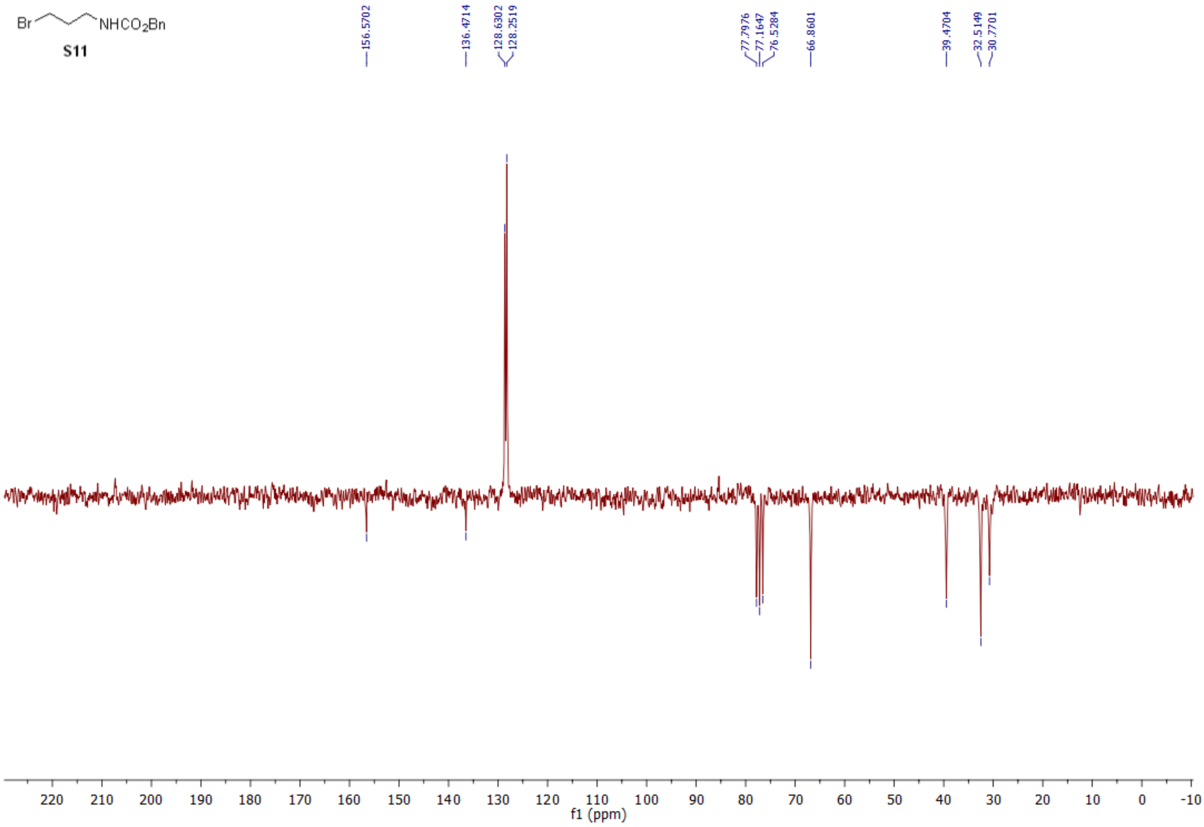

^{1}H (top) and ^{13}C (down) NMR spectra of compound **S13** in $CDCl_3$

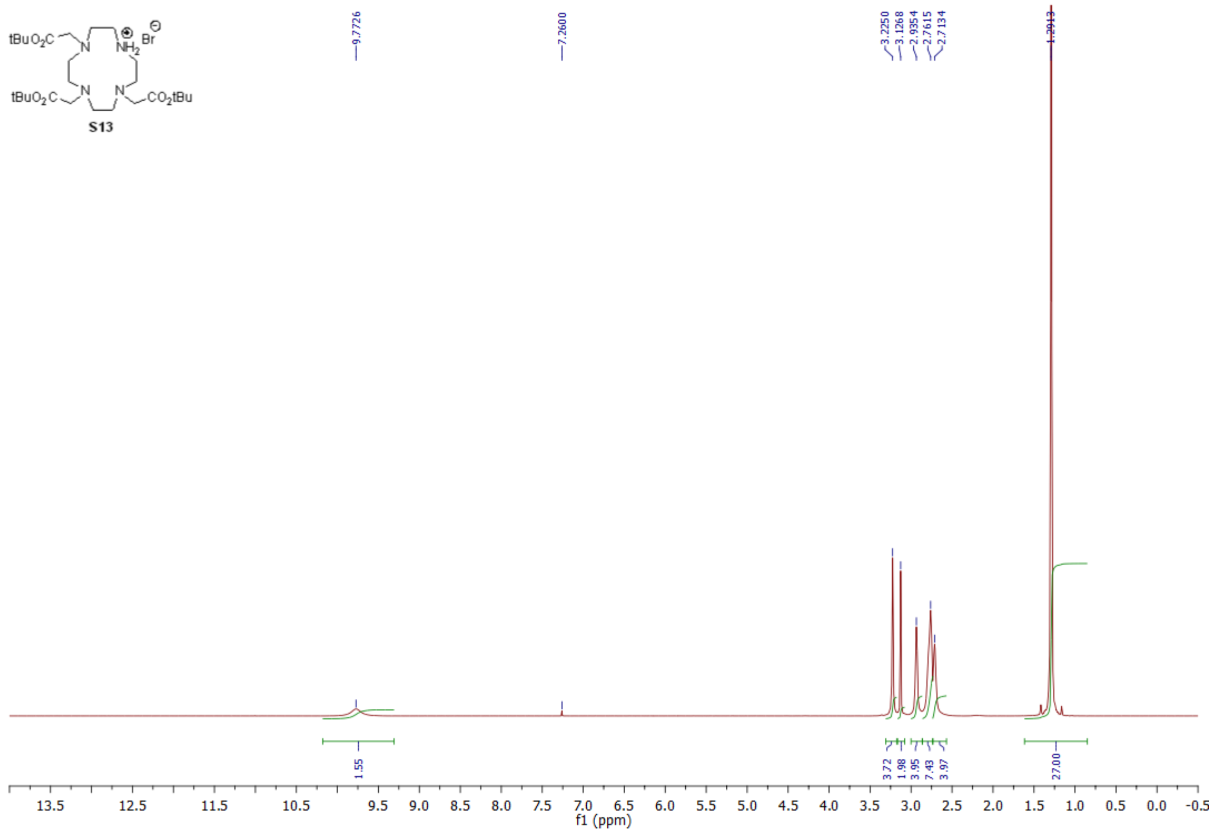

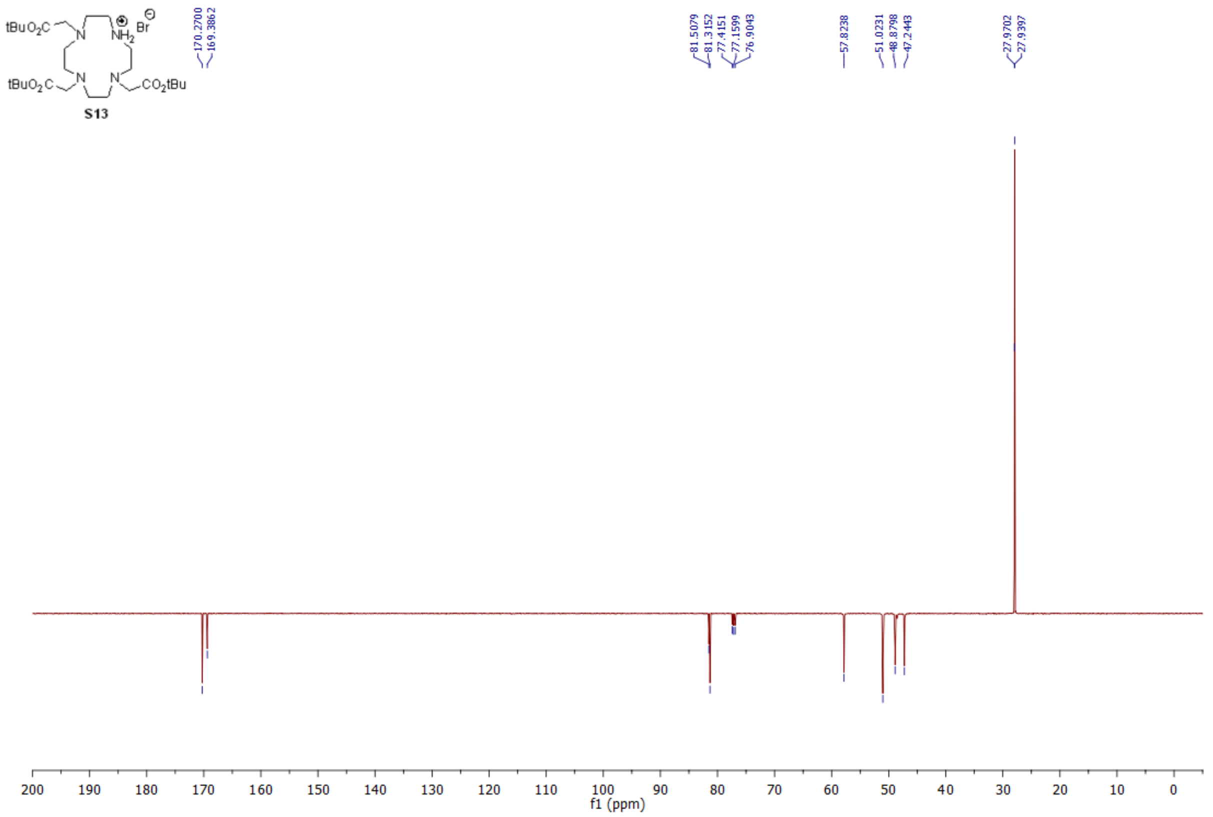

^{1}H (top) and ^{13}C (down) NMR spectra of compound **S14** in CD_3CN

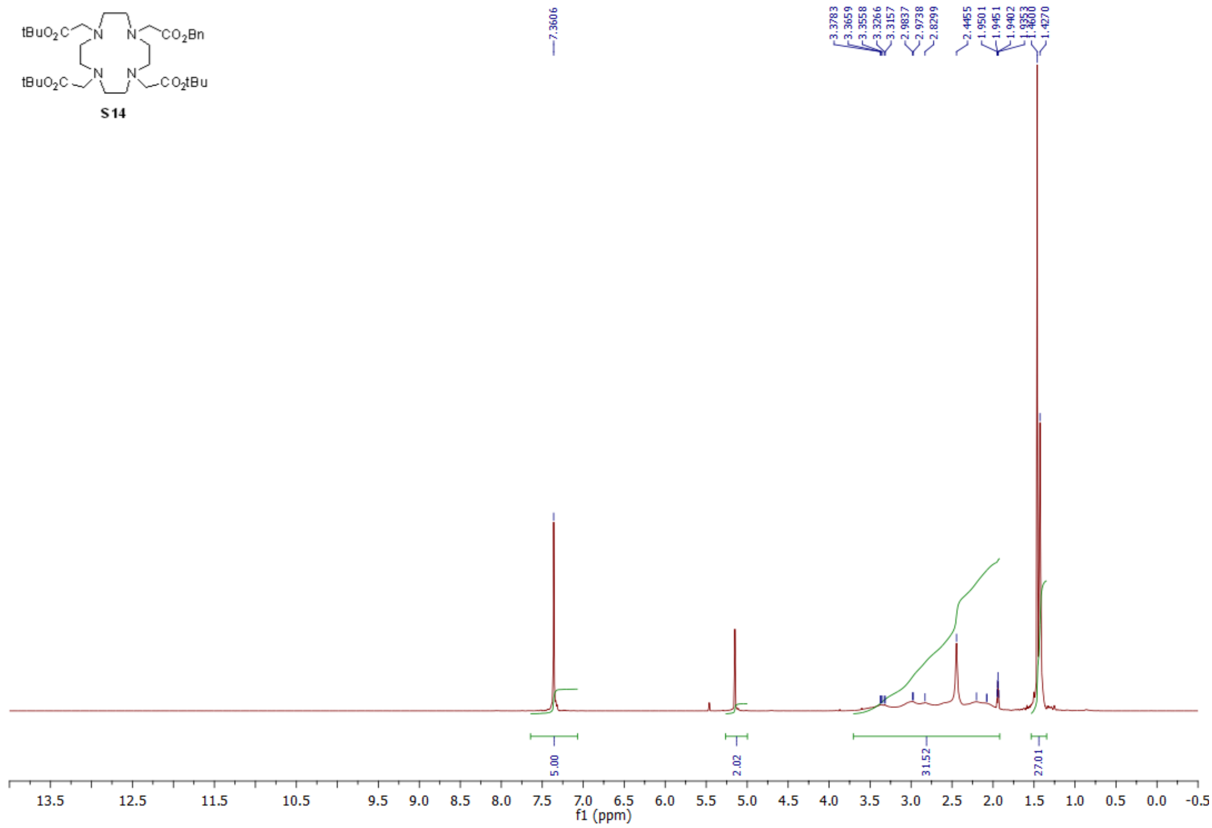

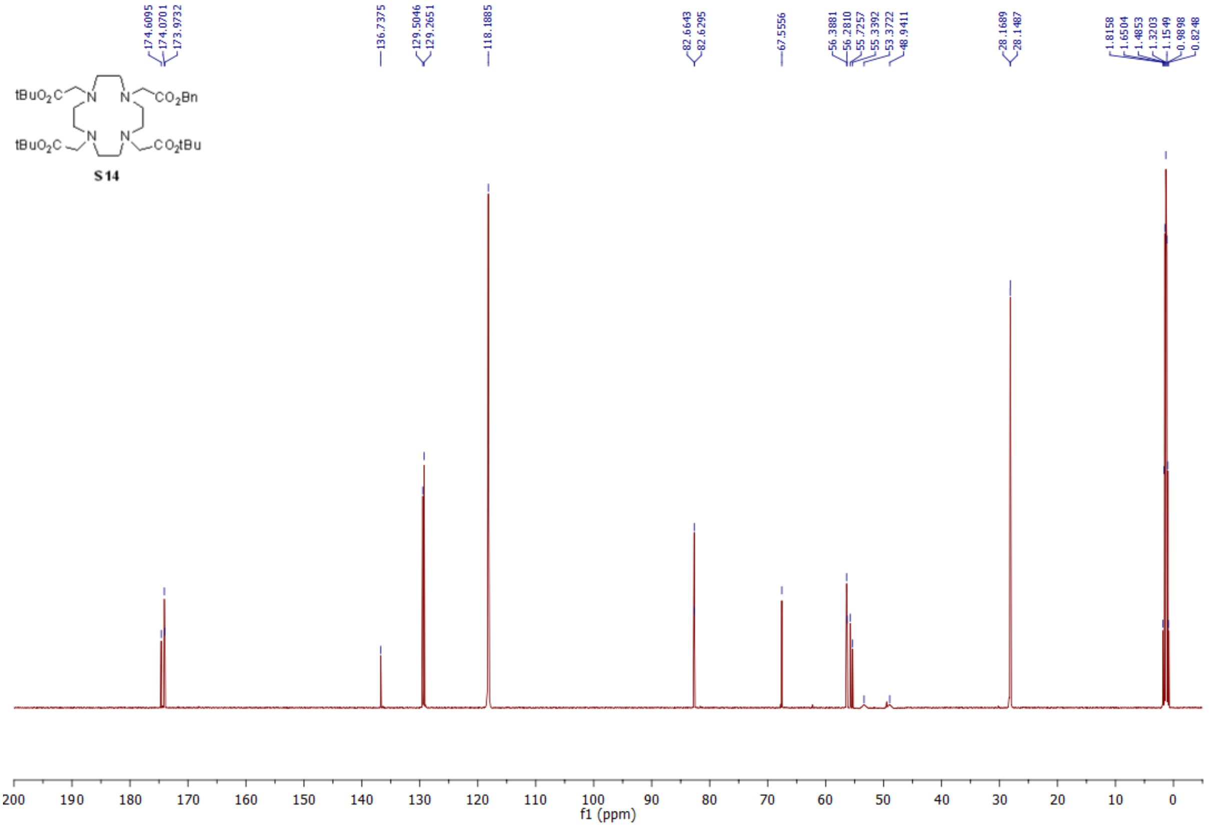

^{1}H (top) and ^{13}C (down) NMR spectra of compound **S15** in $CDCl_3$ and CD_3CN, respectively

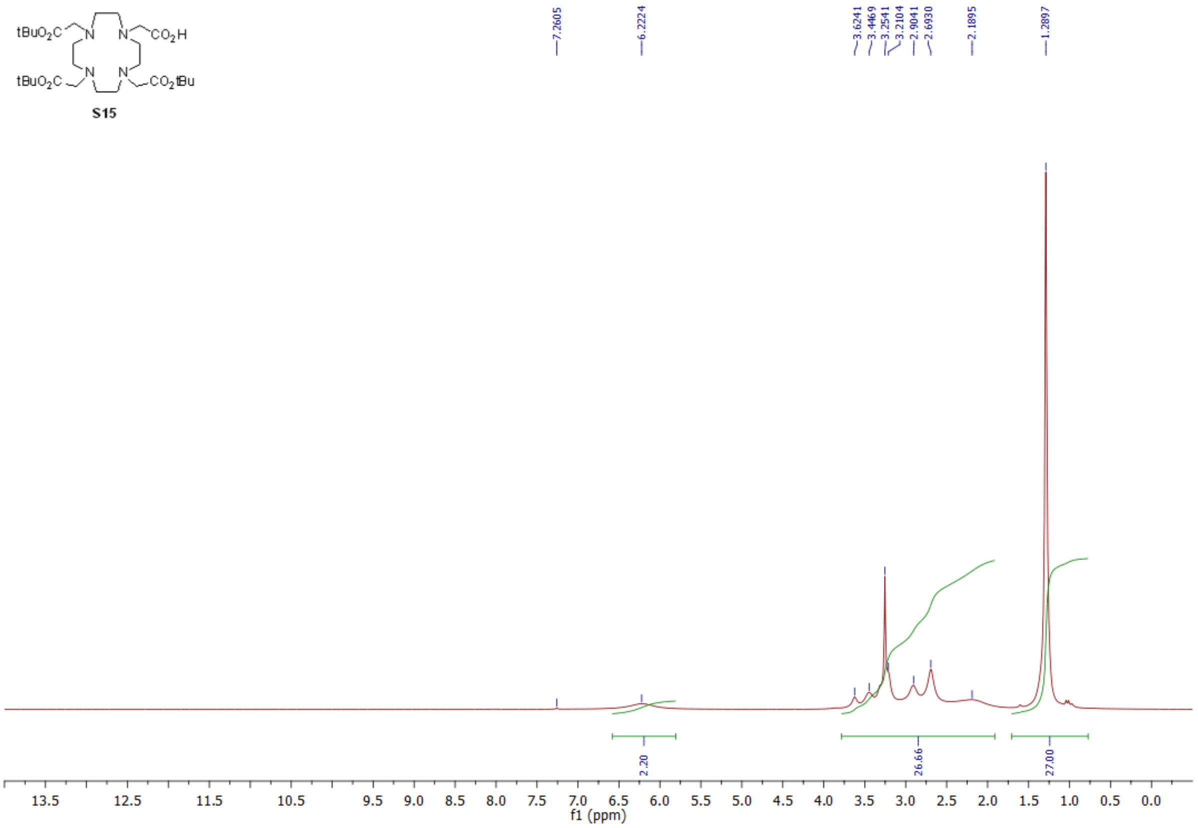

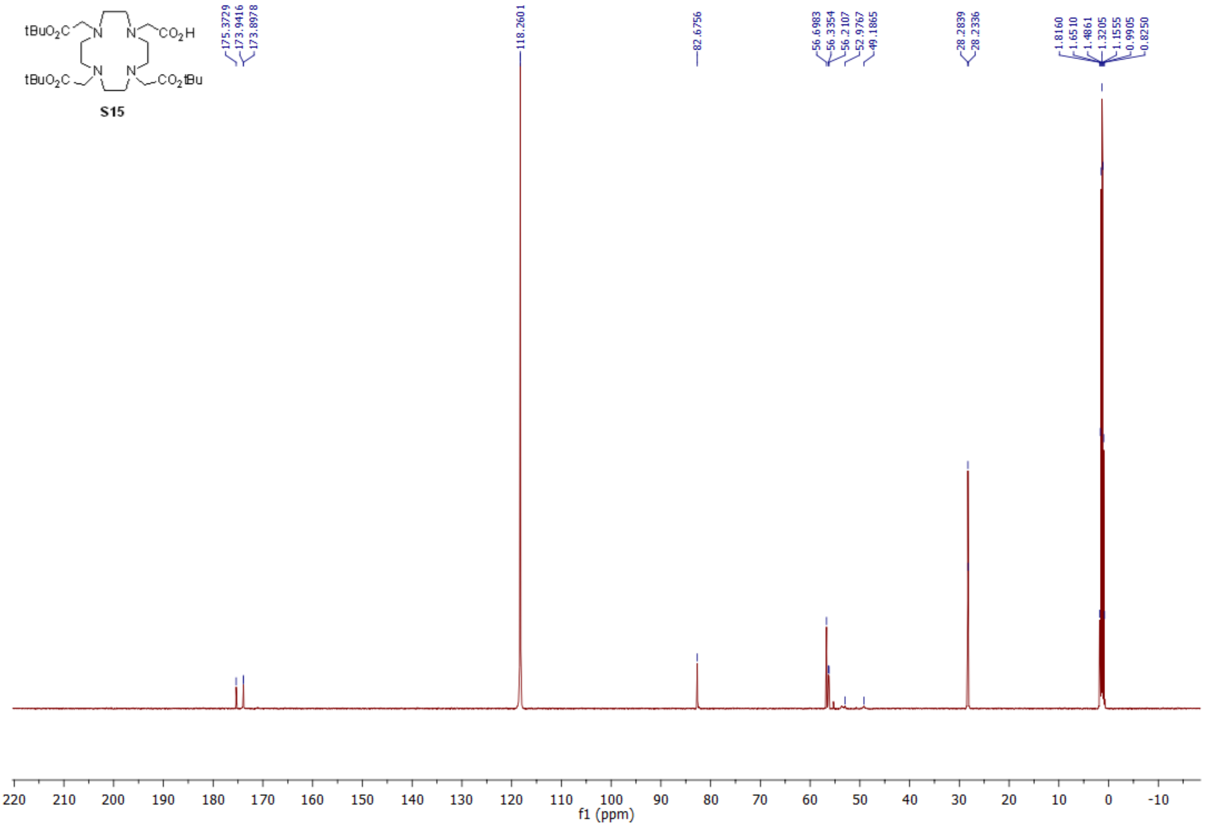

^{1}H (top) and ^{13}C (down) NMR spectra of compound **S17** in $CDCl_3$

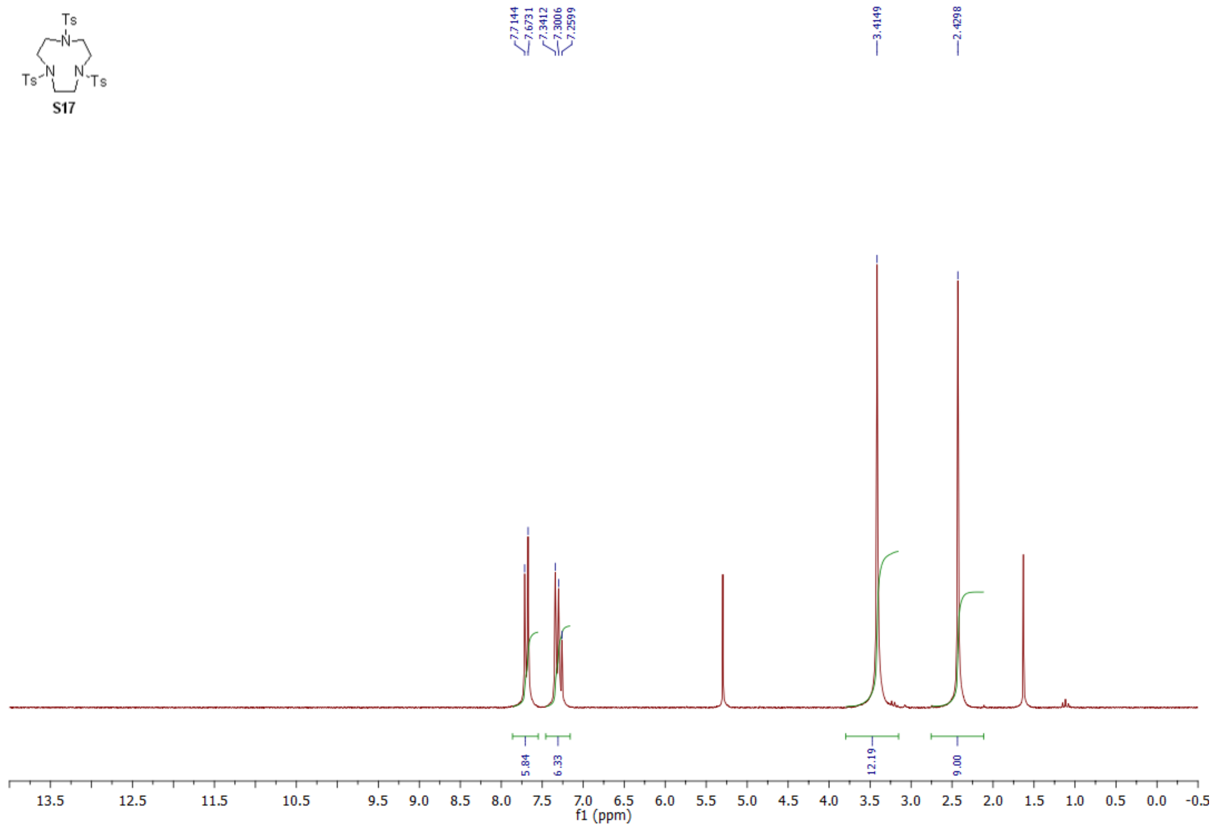

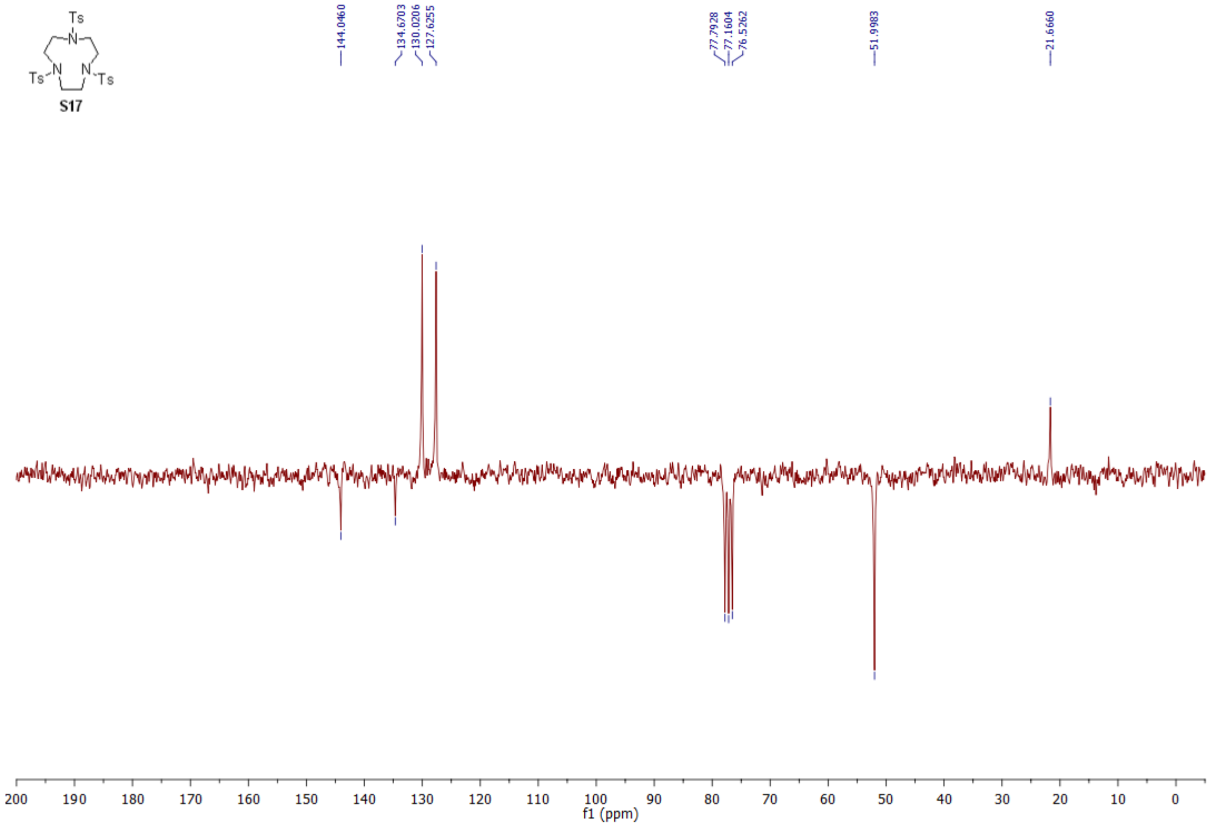

^1H (top) and ^{13}C (down) NMR spectra of compound **S18** in D_2O+1,4-dioxane

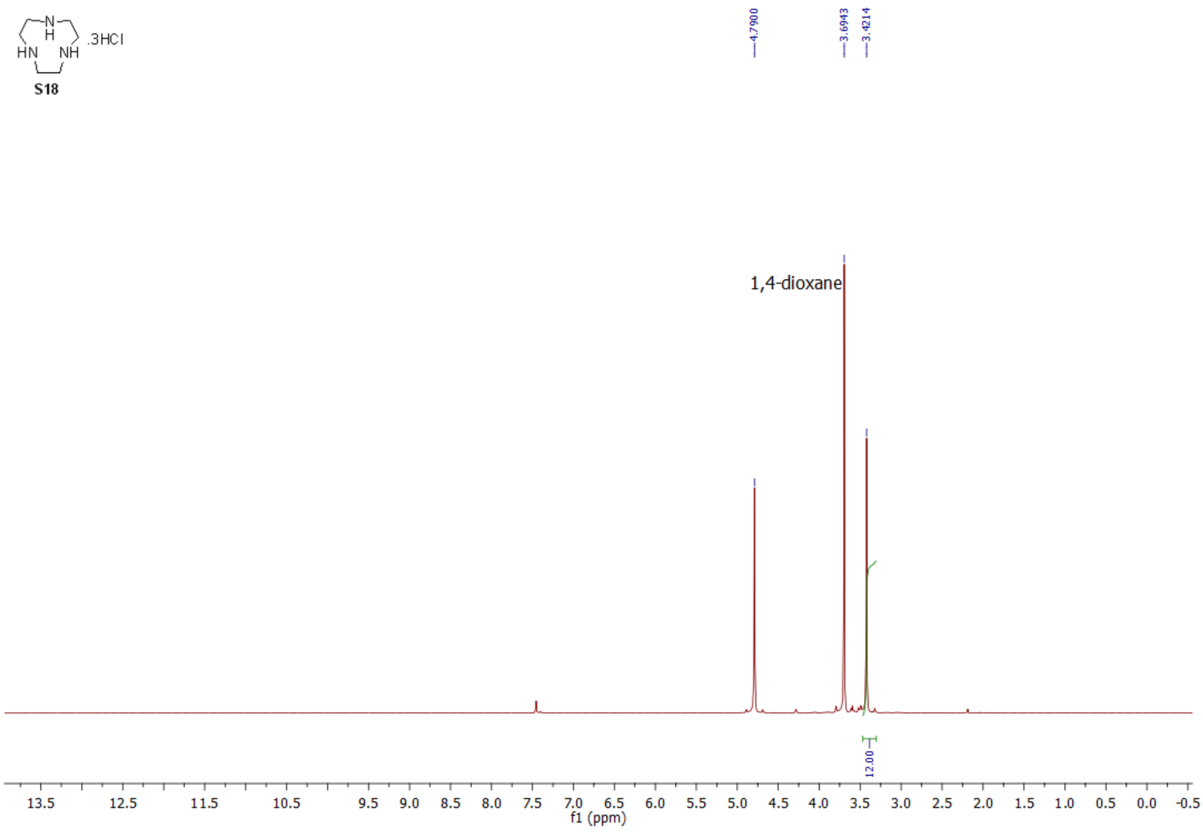

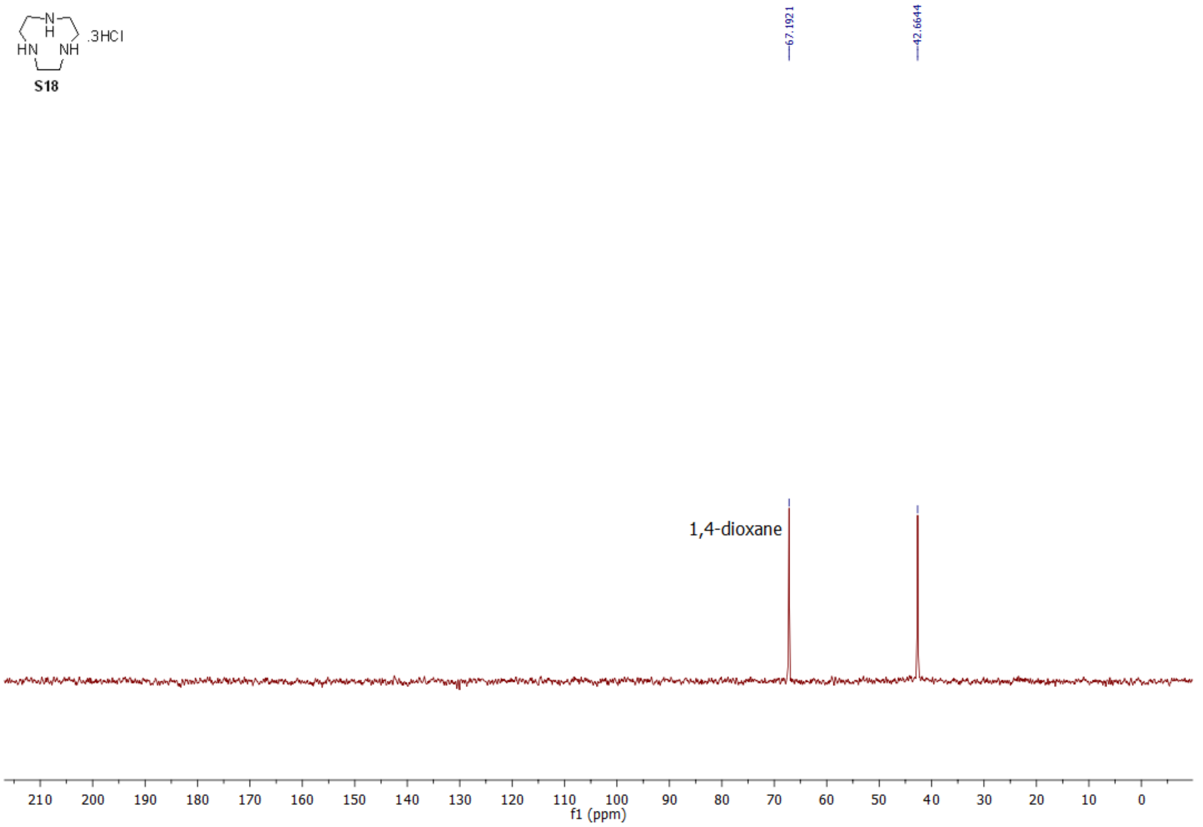

^{1}H (top) and ^{13}C (down) NMR spectra of compound **S19** in $CDCl_3$

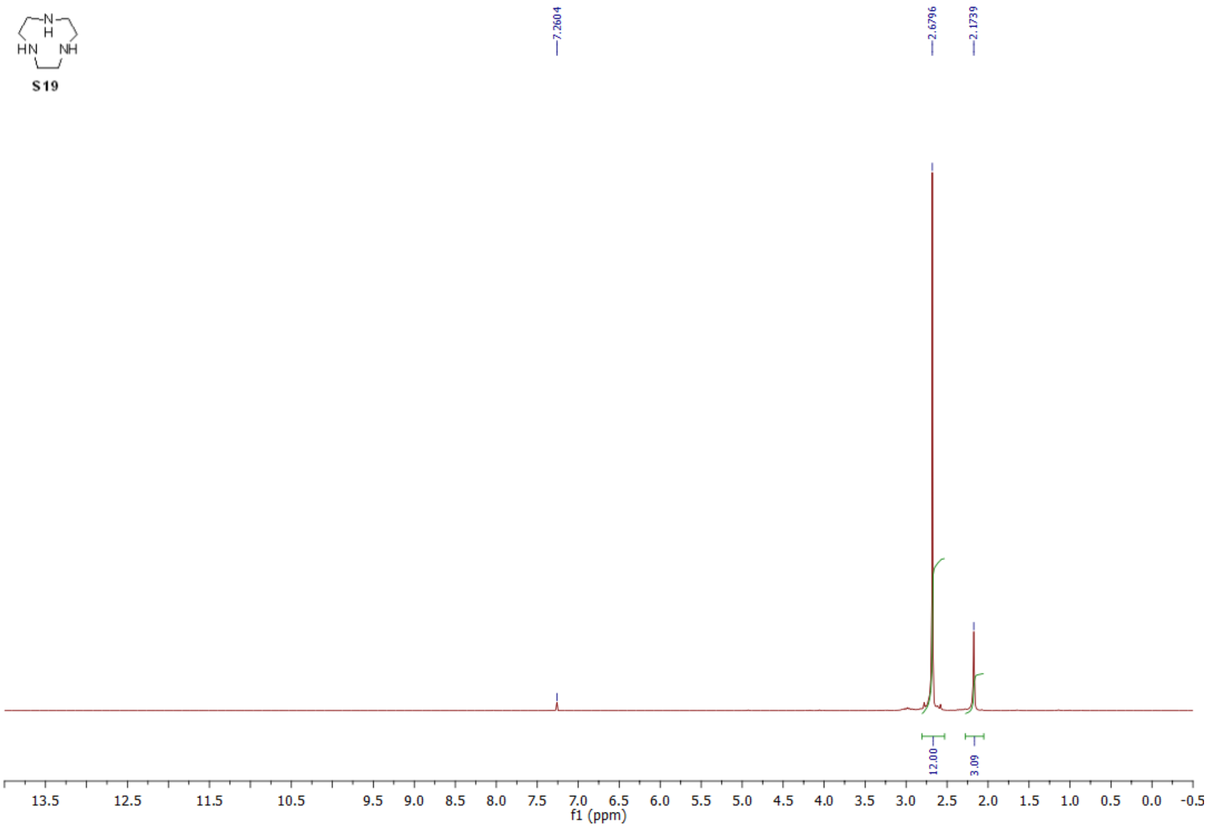

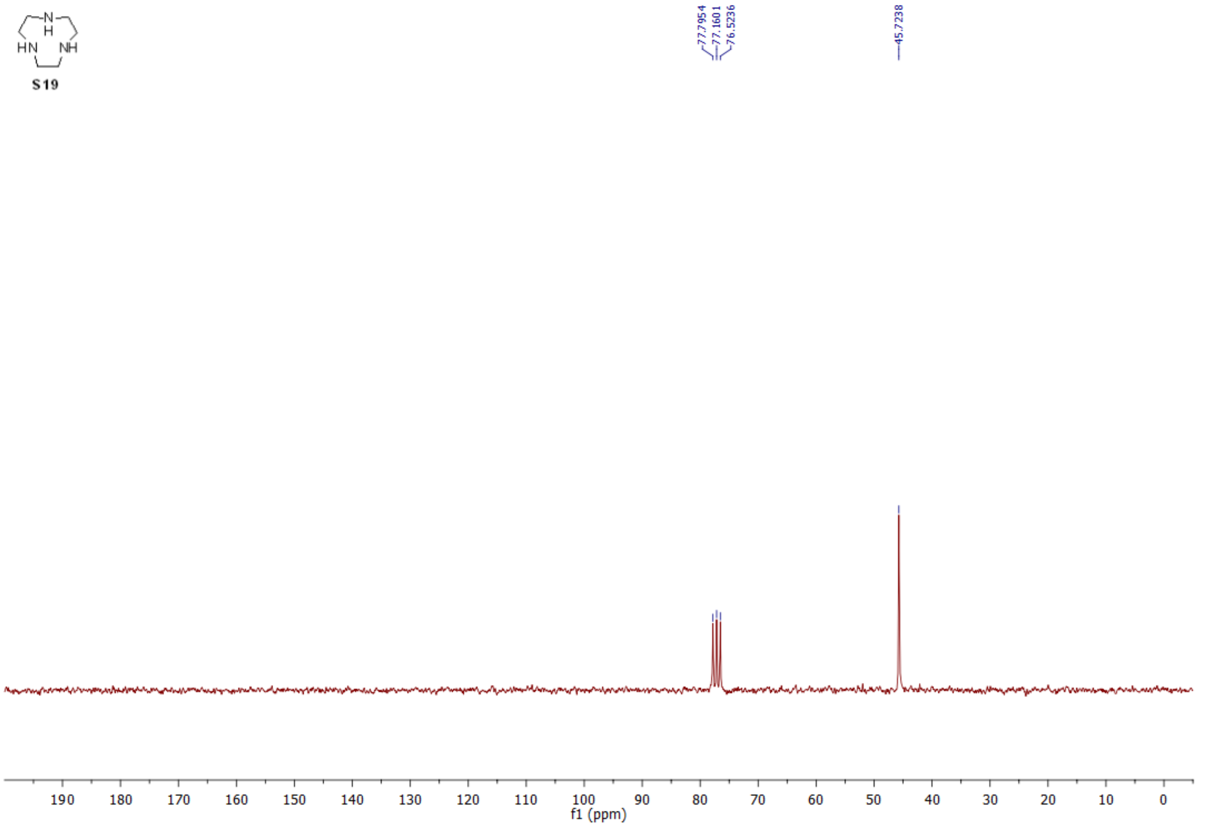

^{1}H (top) and ^{13}C (down) NMR spectra of compound **S21** in conc. HCl/D_2O (with 1,4-dioxane as internal reference for ^{13}C NMR)

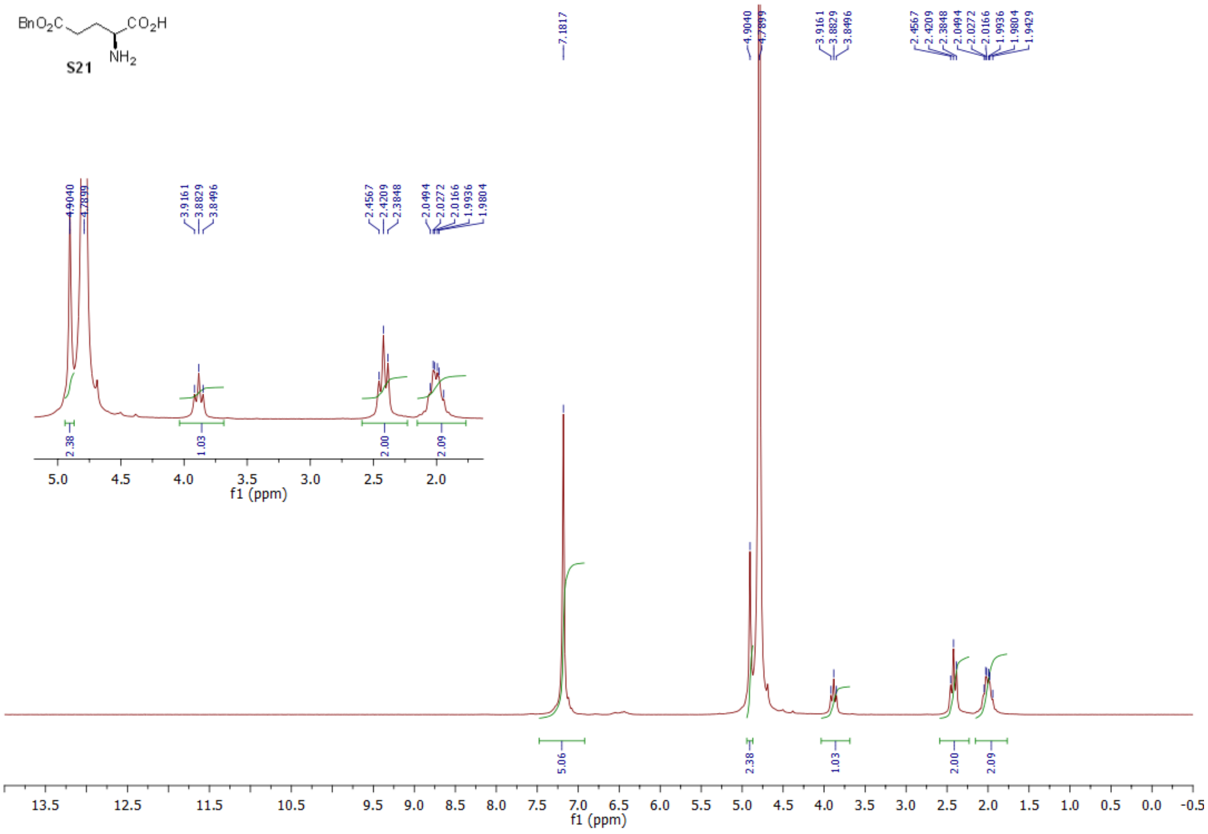

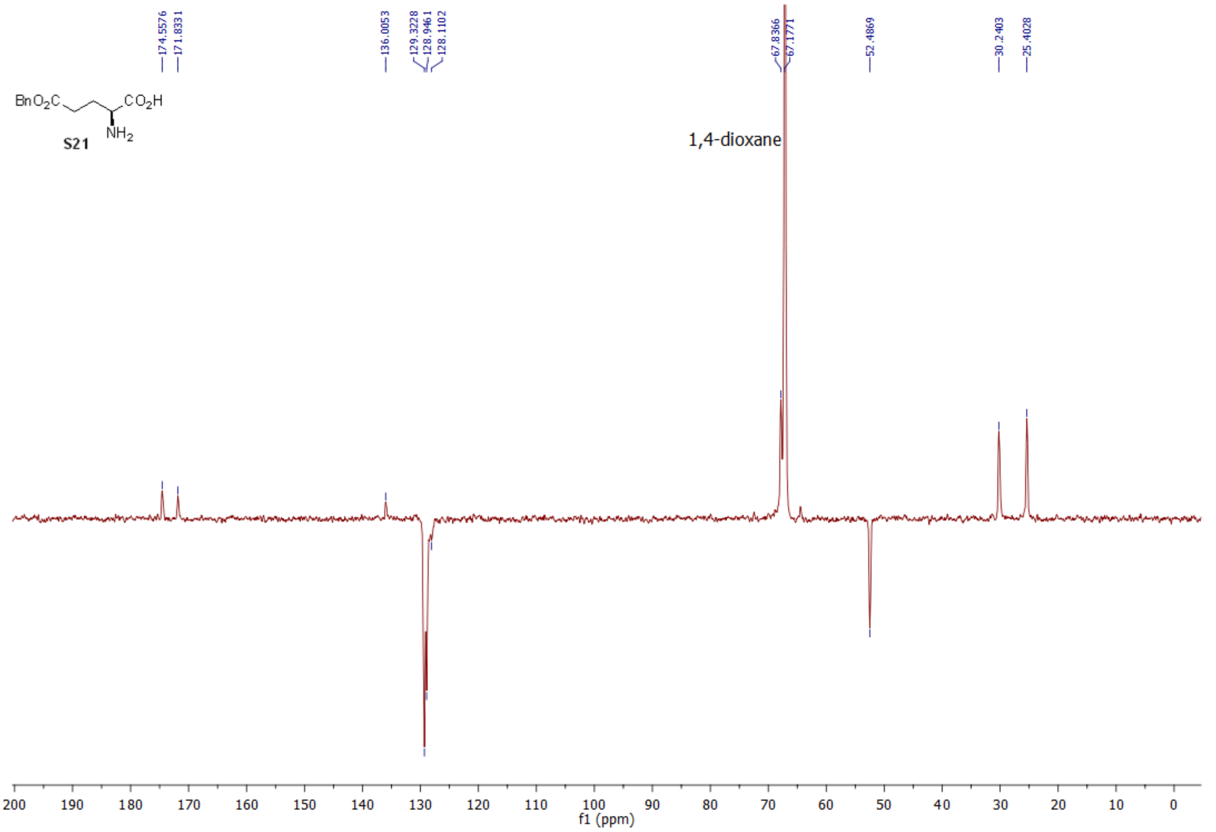

^{1}H (top) and ^{13}C (down) NMR spectra of compound **S22** in $CDCl_3$

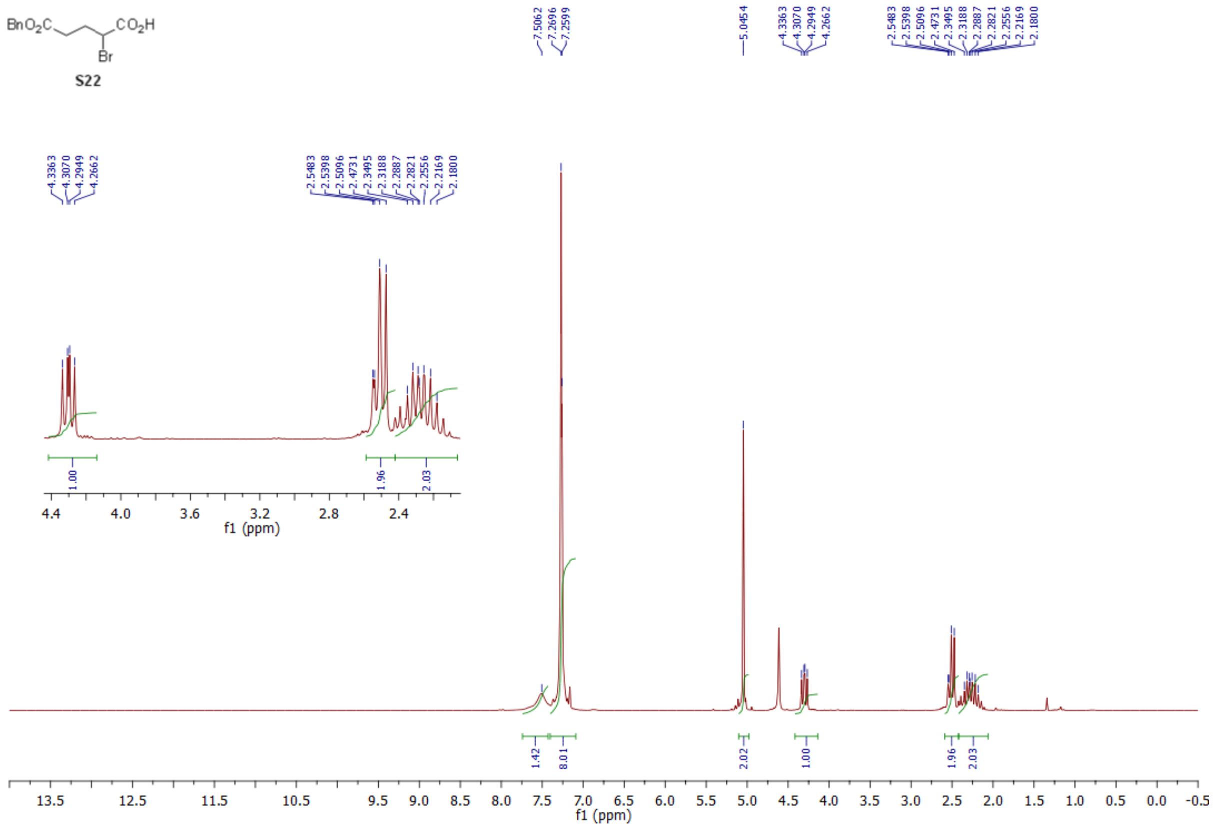

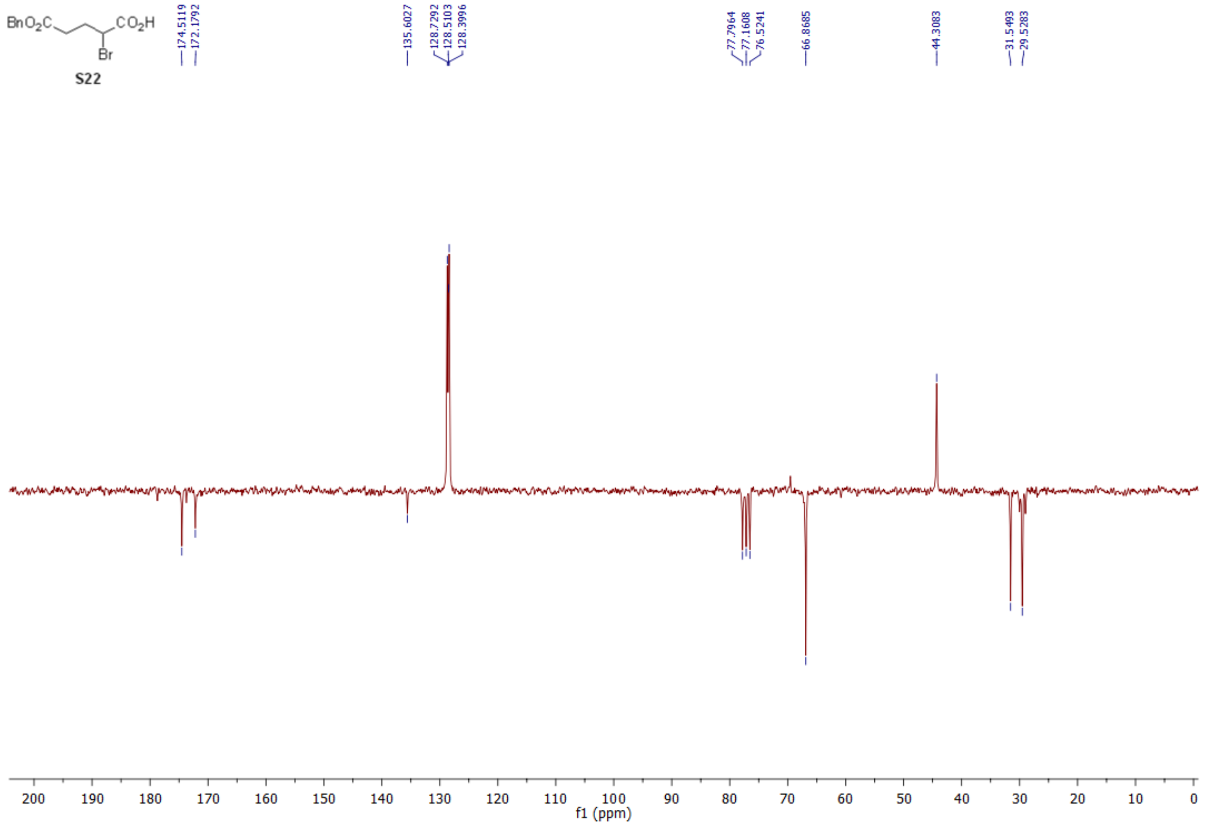

^{1}H (top) and ^{13}C (down) NMR spectra of compound **S23** in $CDCl_3$

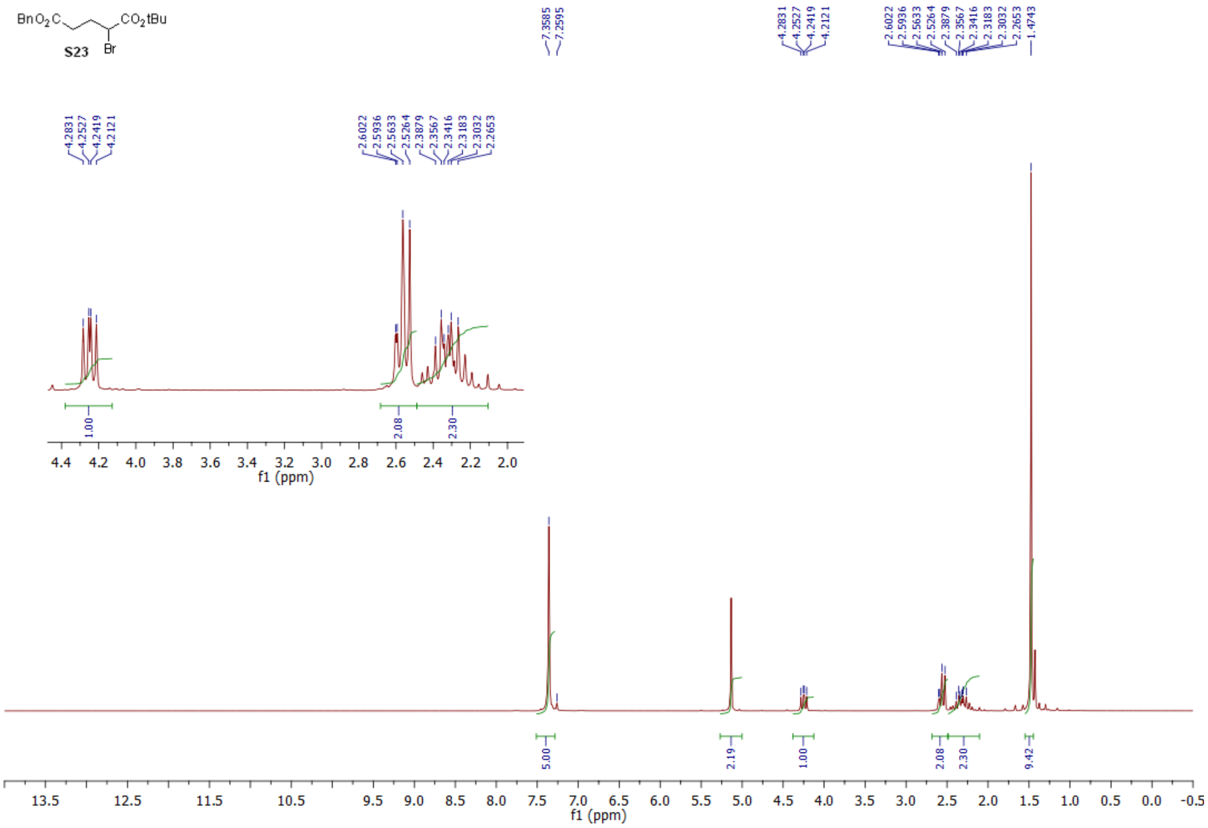

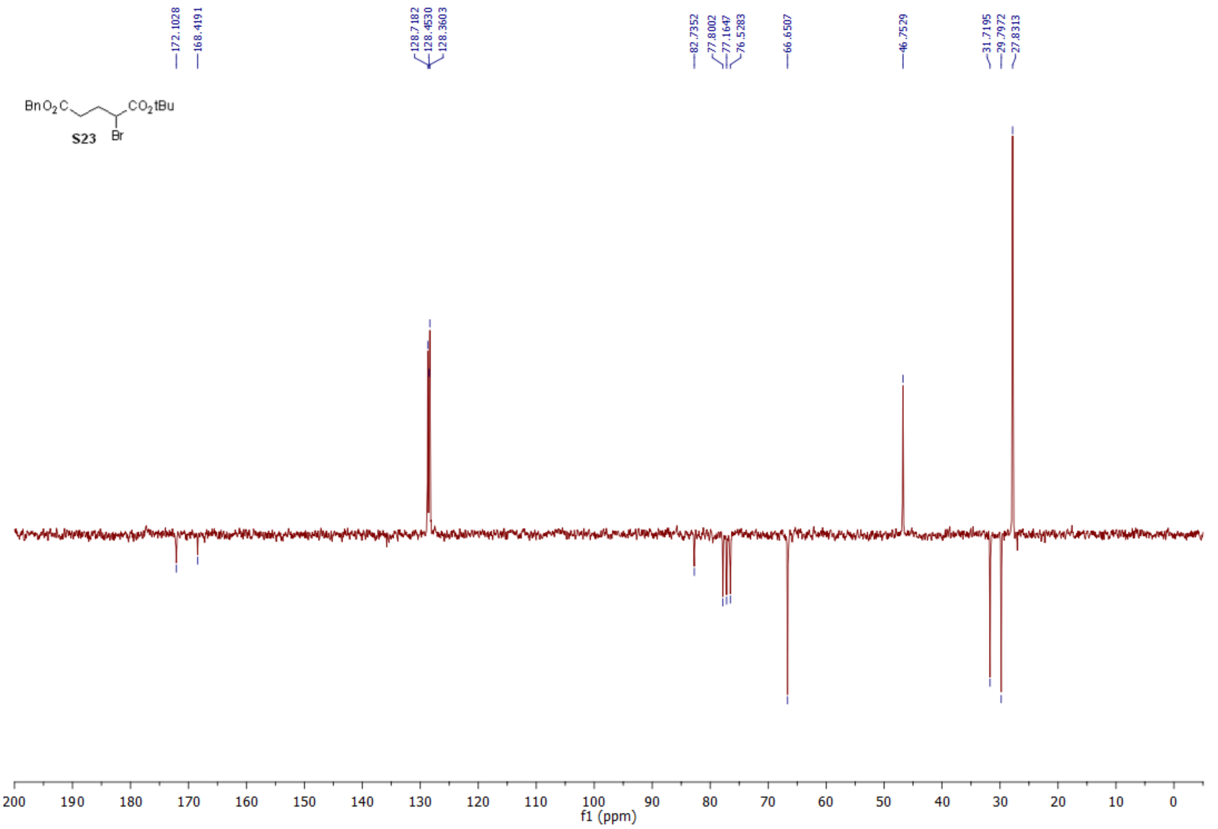

^1H (top) and ^{13}C (down) NMR spectra of compound **S24** in $CDCl_3$

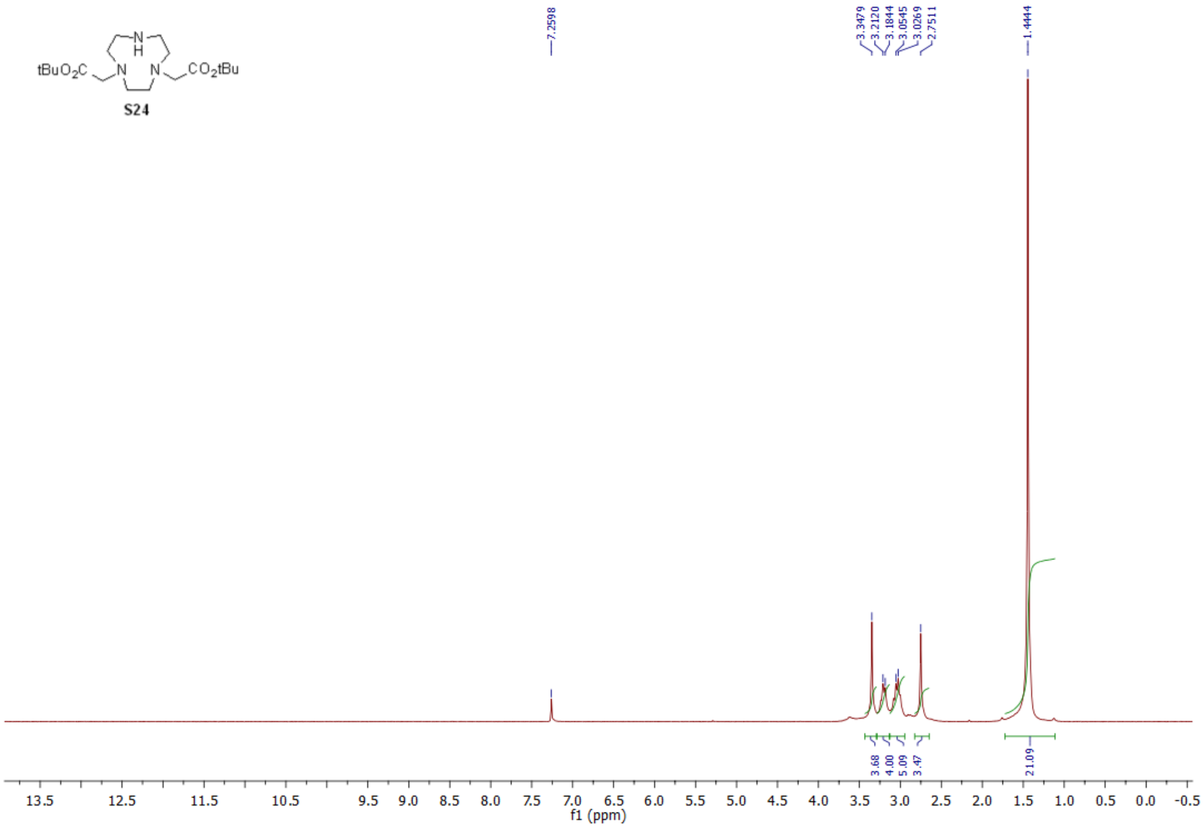

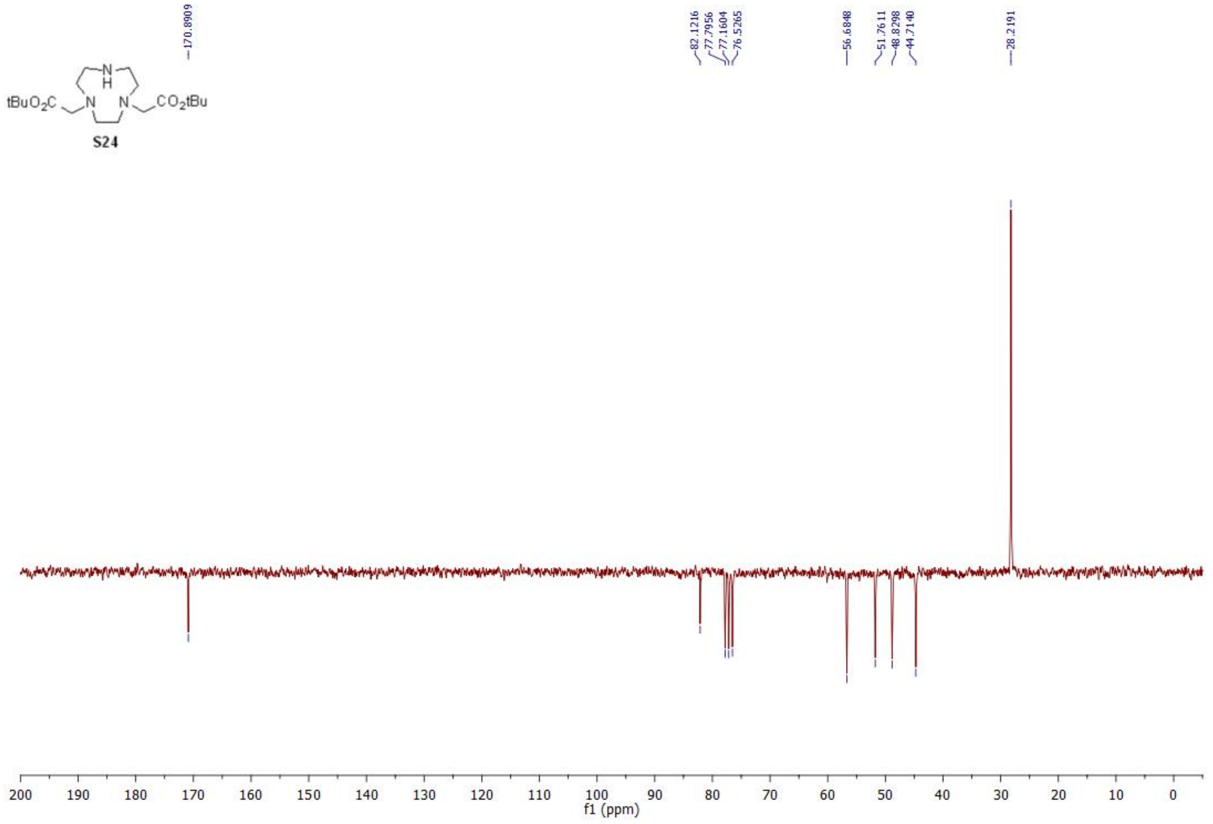

^{1}H (top) and ^{13}C (down) NMR spectra of compound **S25** in $CDCl_3$

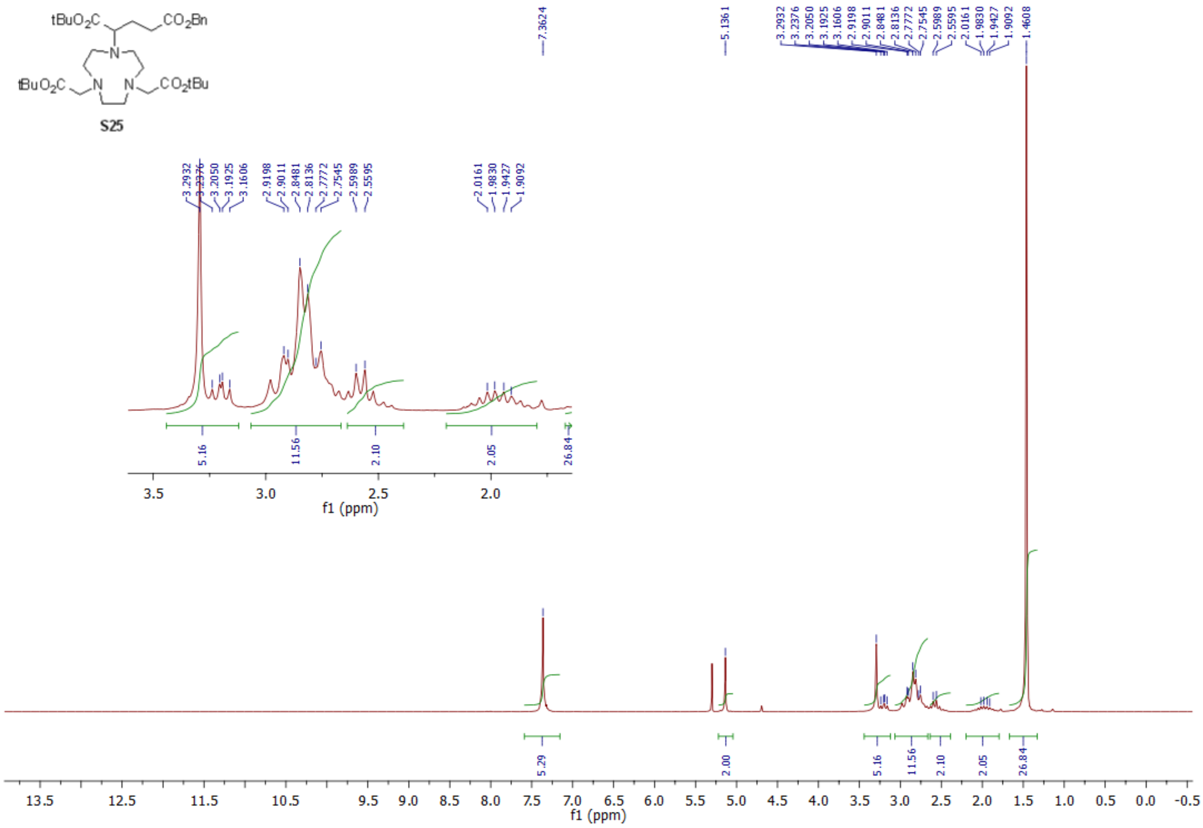

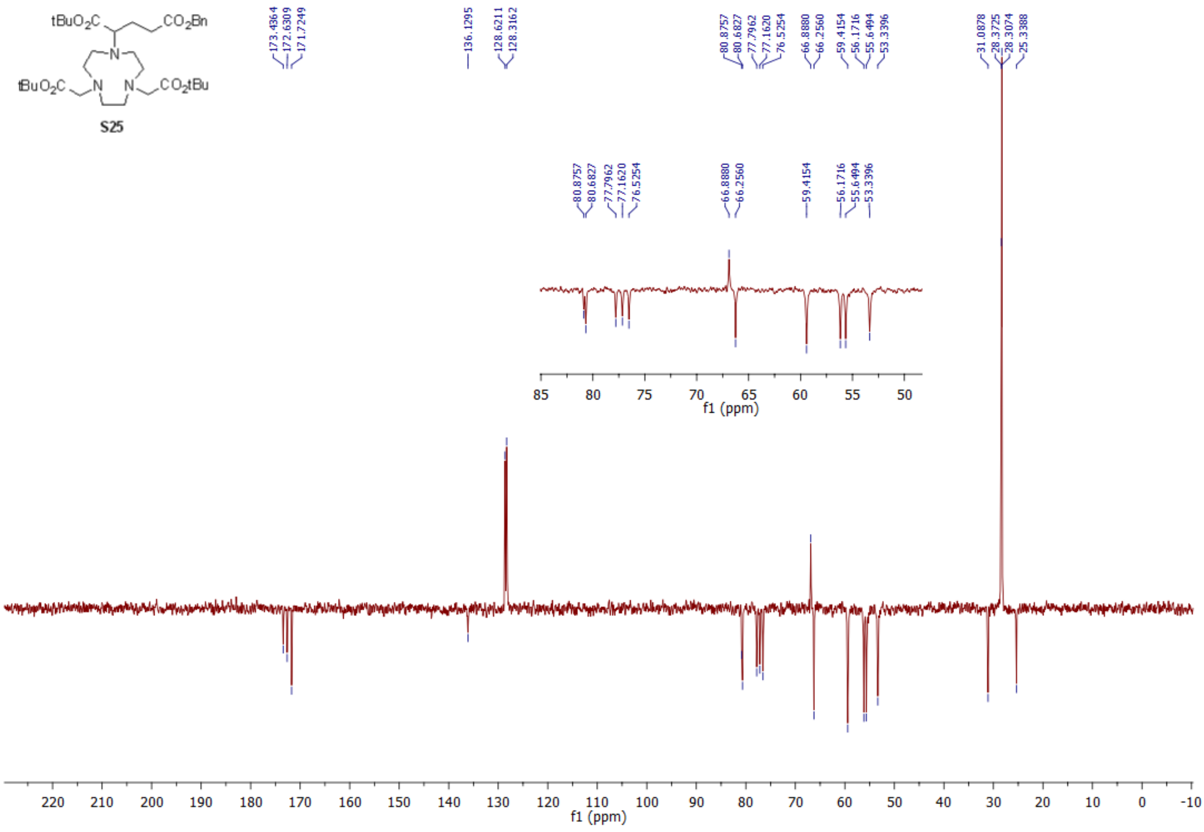

^{1}H (top) and ^{13}C (down) NMR spectra of compound **S26** in $CDCl_3$

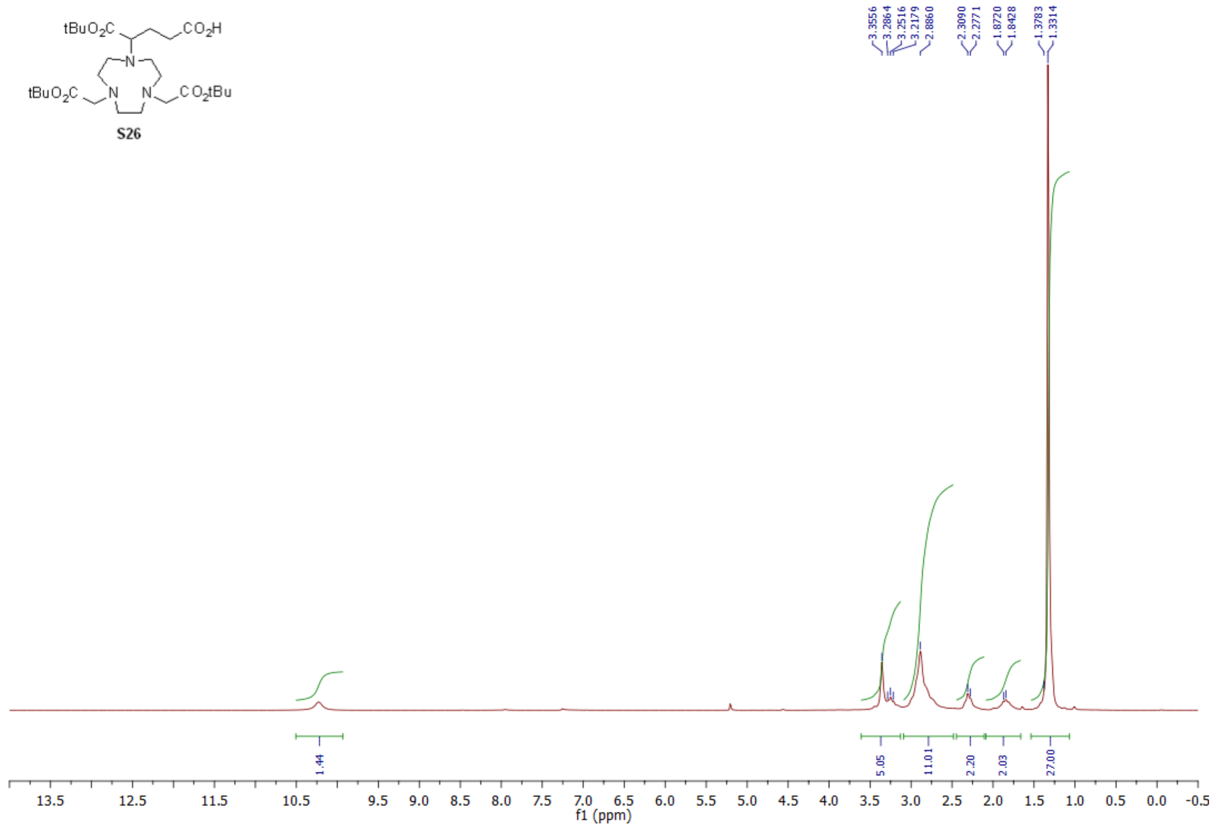

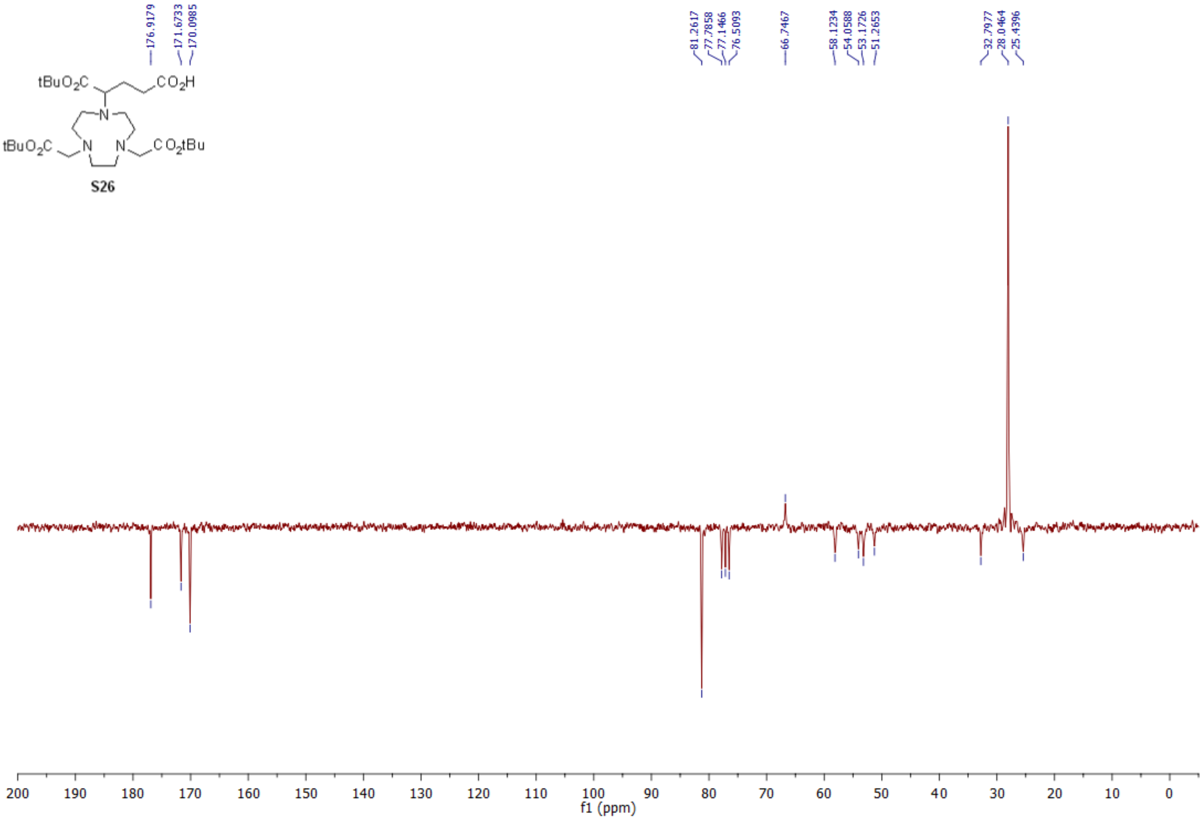

^{1}H (top) and ^{13}C (down) NMR spectra of compound **S28** in $CDCl_3$

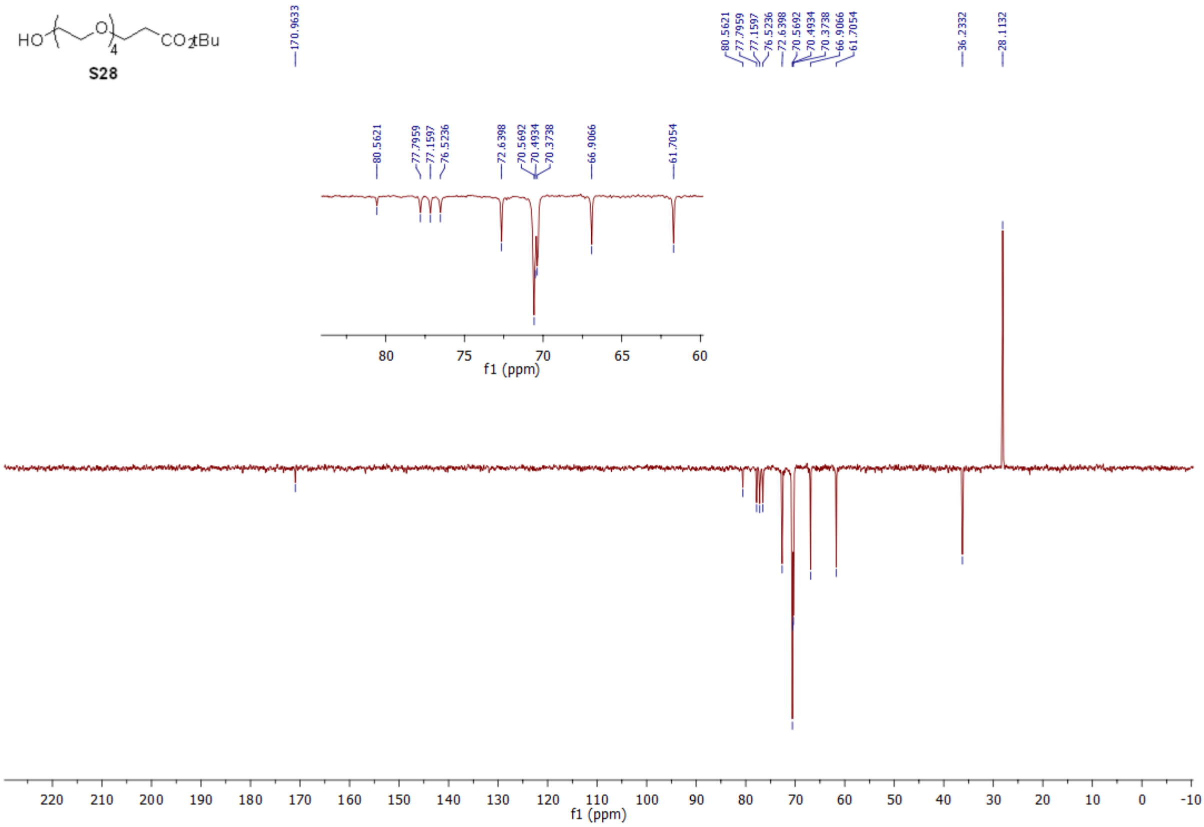

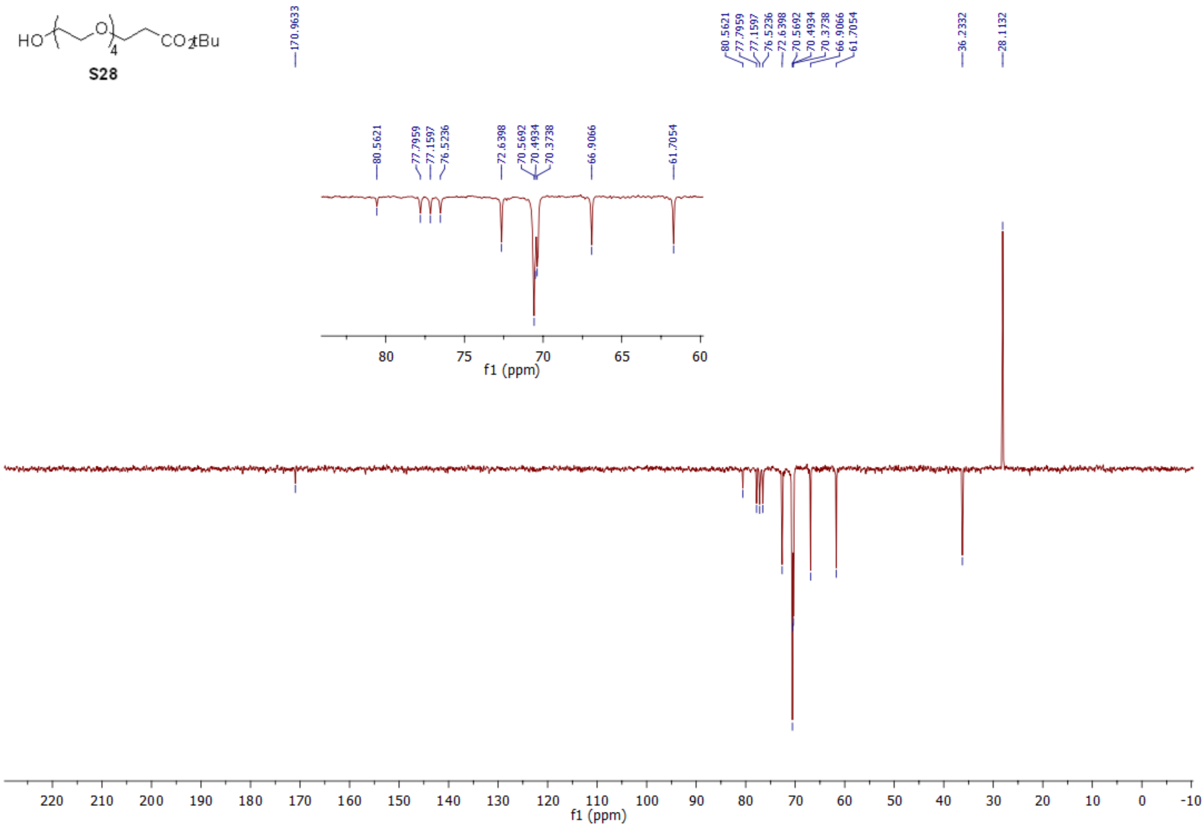

^{1}H (top) and ^{13}C (down) NMR spectra of compound **S29** in $CDCl_3$

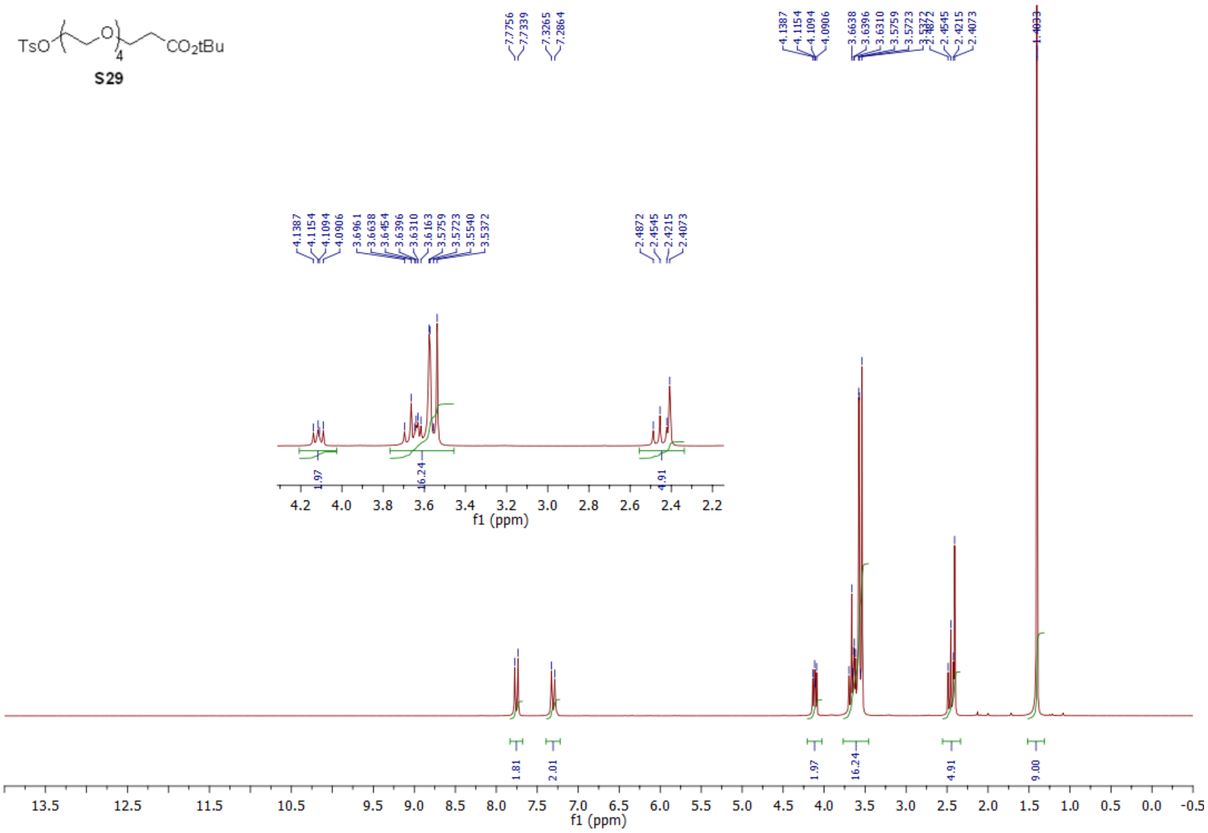

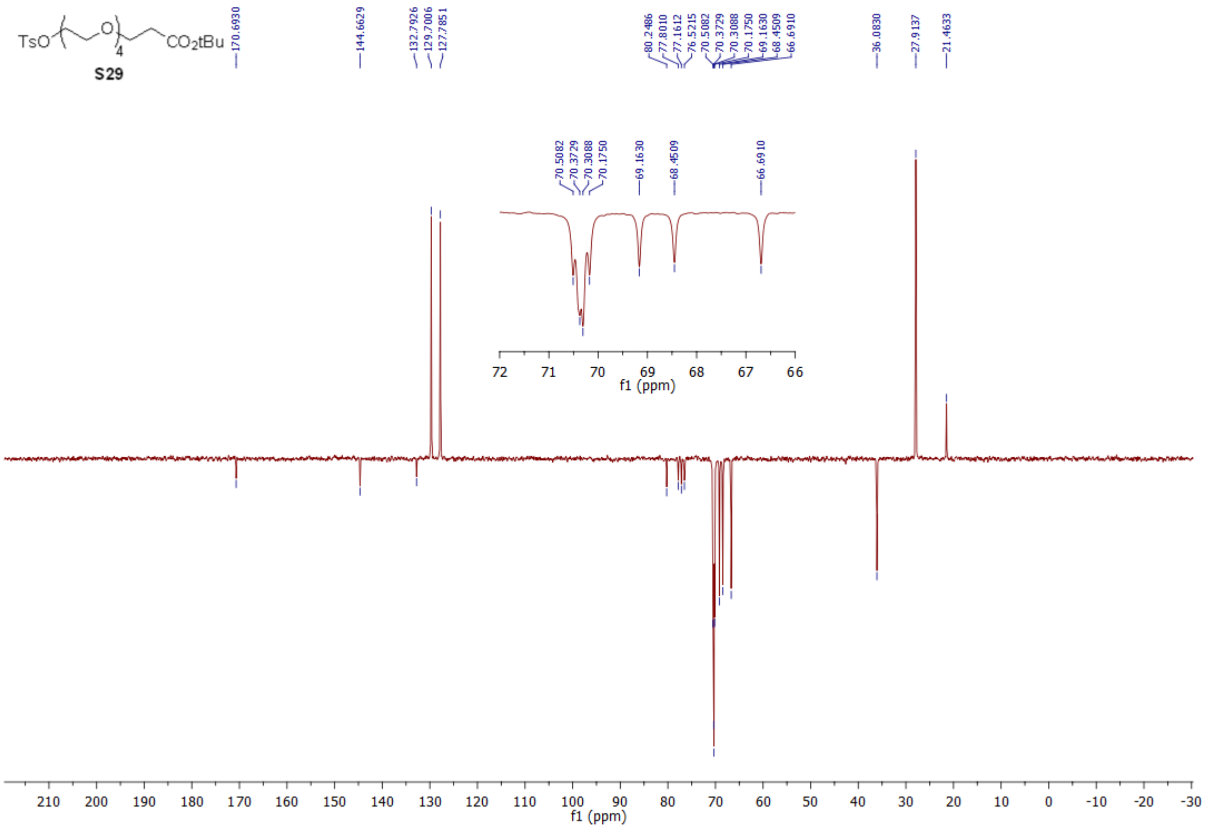

^{1}H (top) and ^{13}C (down) NMR spectra of compound **S30** in $CDCl_3$

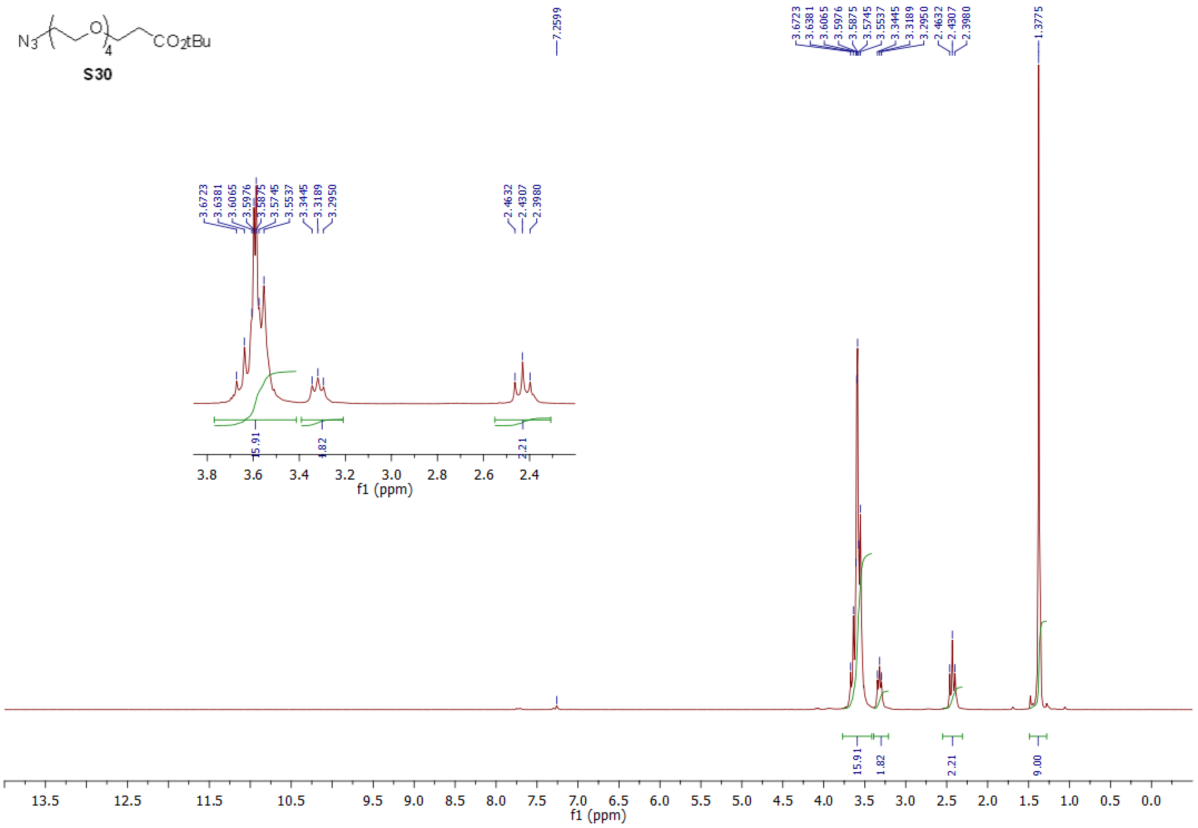

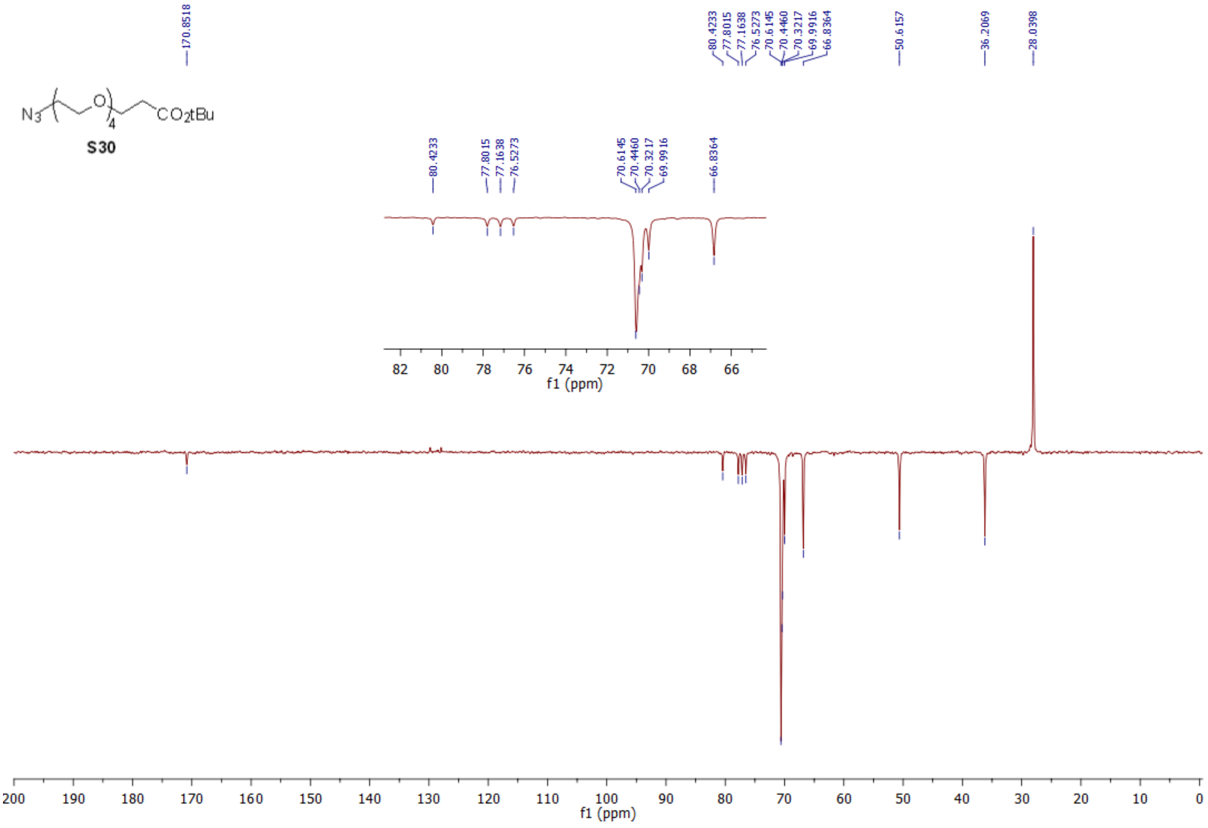

^{1}H (top) and ^{13}C (down) NMR spectra of compound **S31** in $CDCl_3$

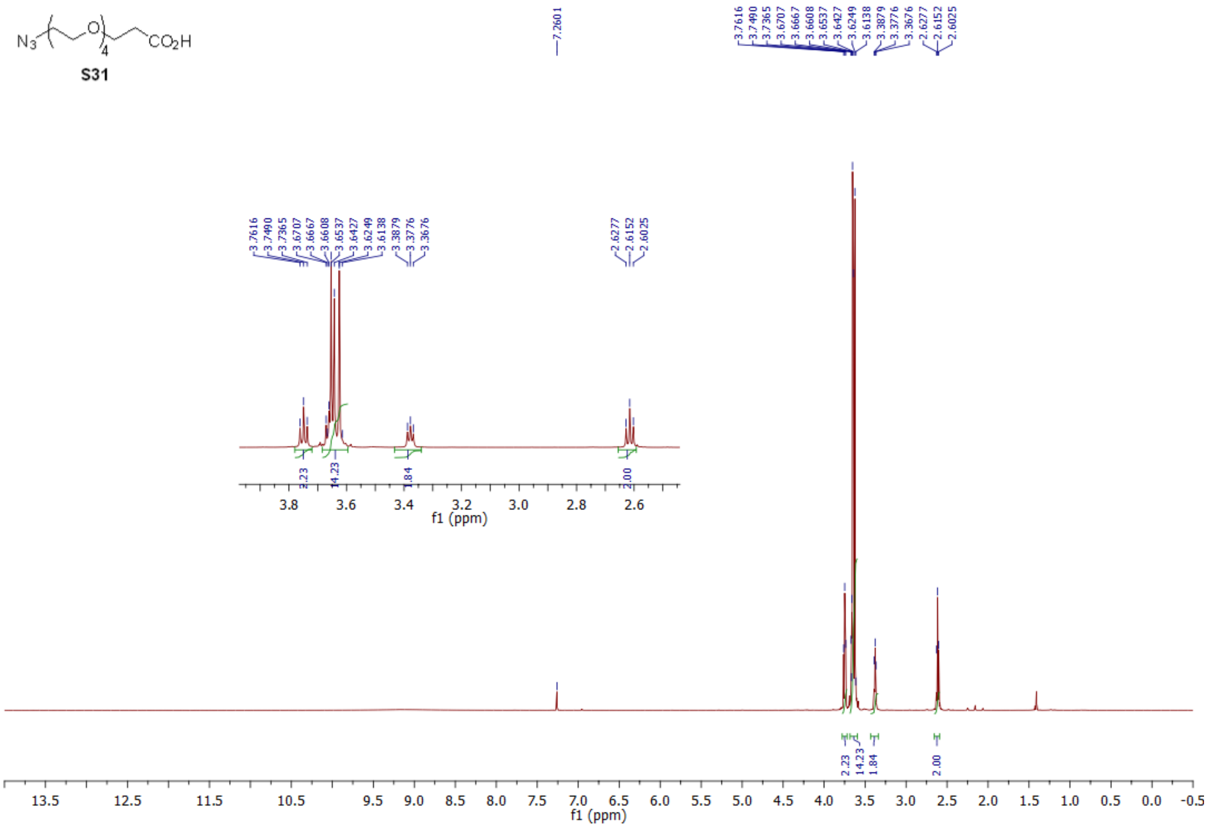

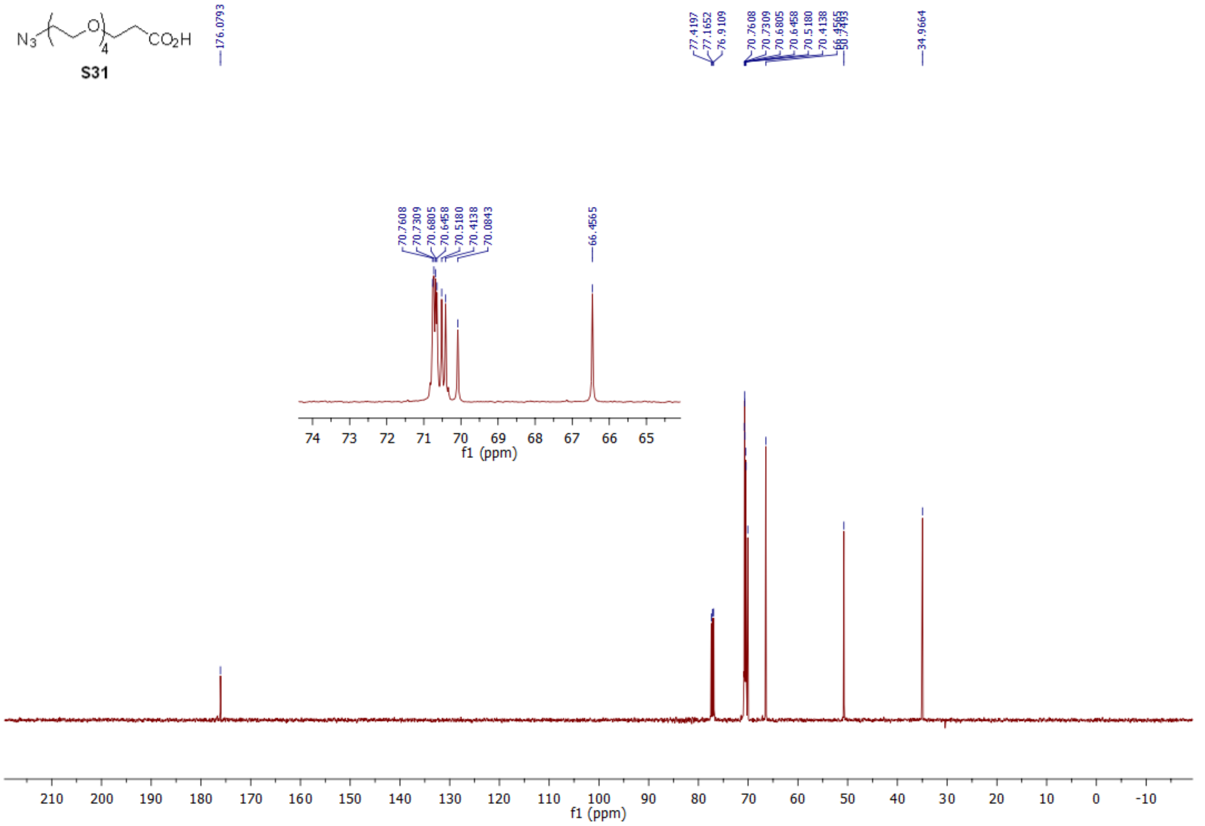

^{1}H (top) and ^{13}C (down) NMR spectra of compound **S32** in $CDCl_3$

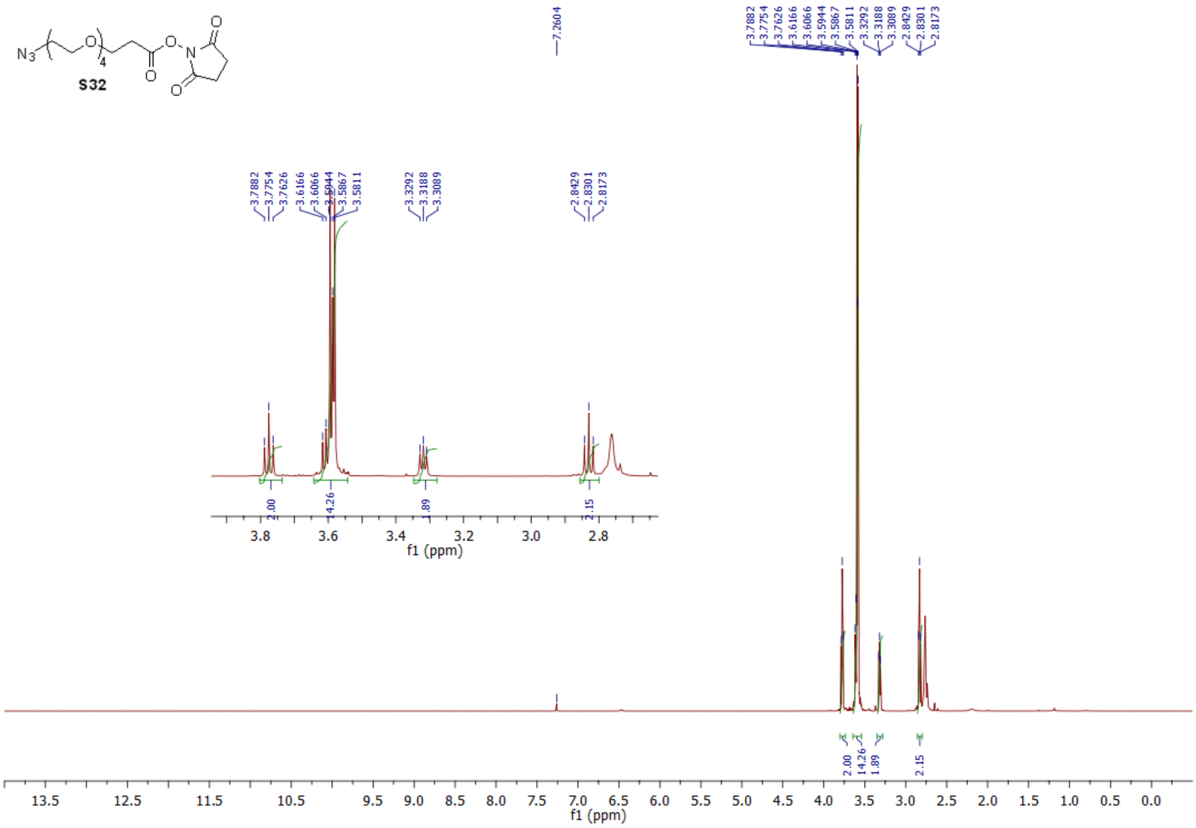

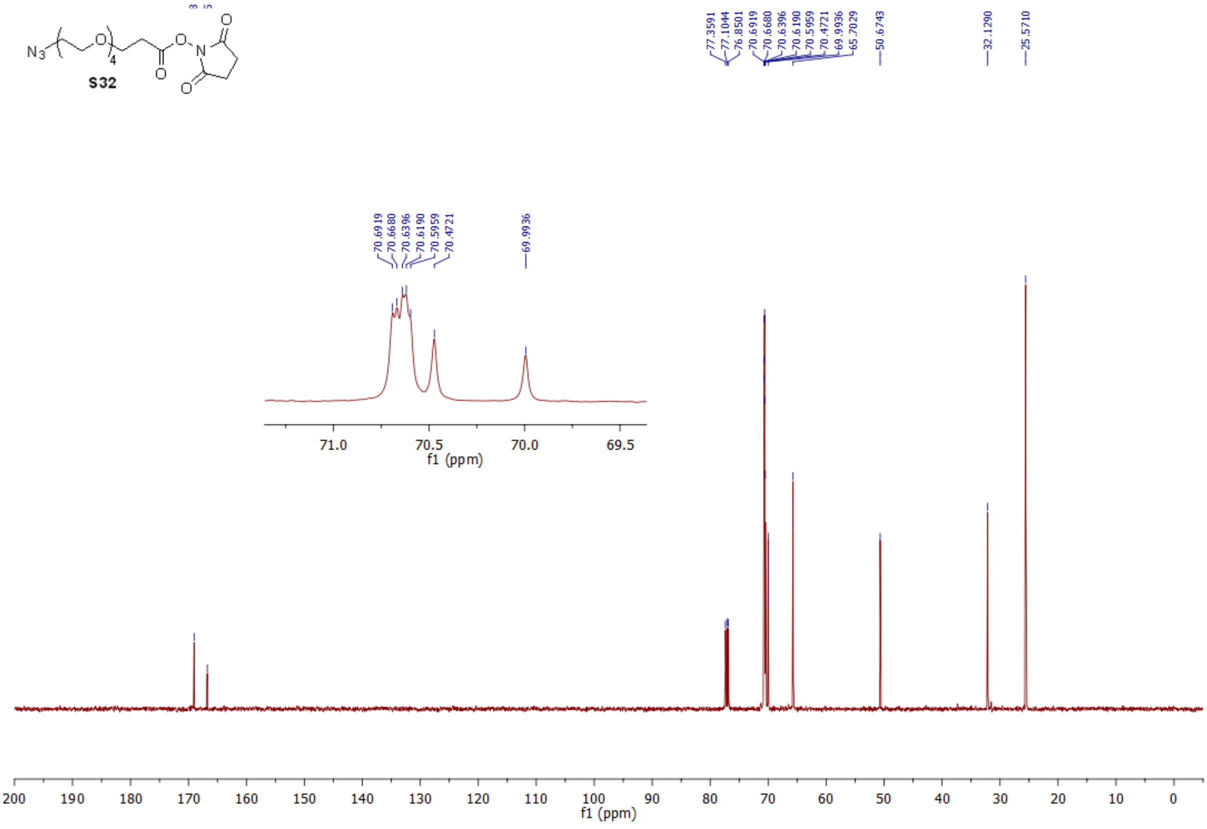

^{1}H (top) and ^{13}C (down) NMR spectra of compound **2** in D_2O+1,4-dioxane

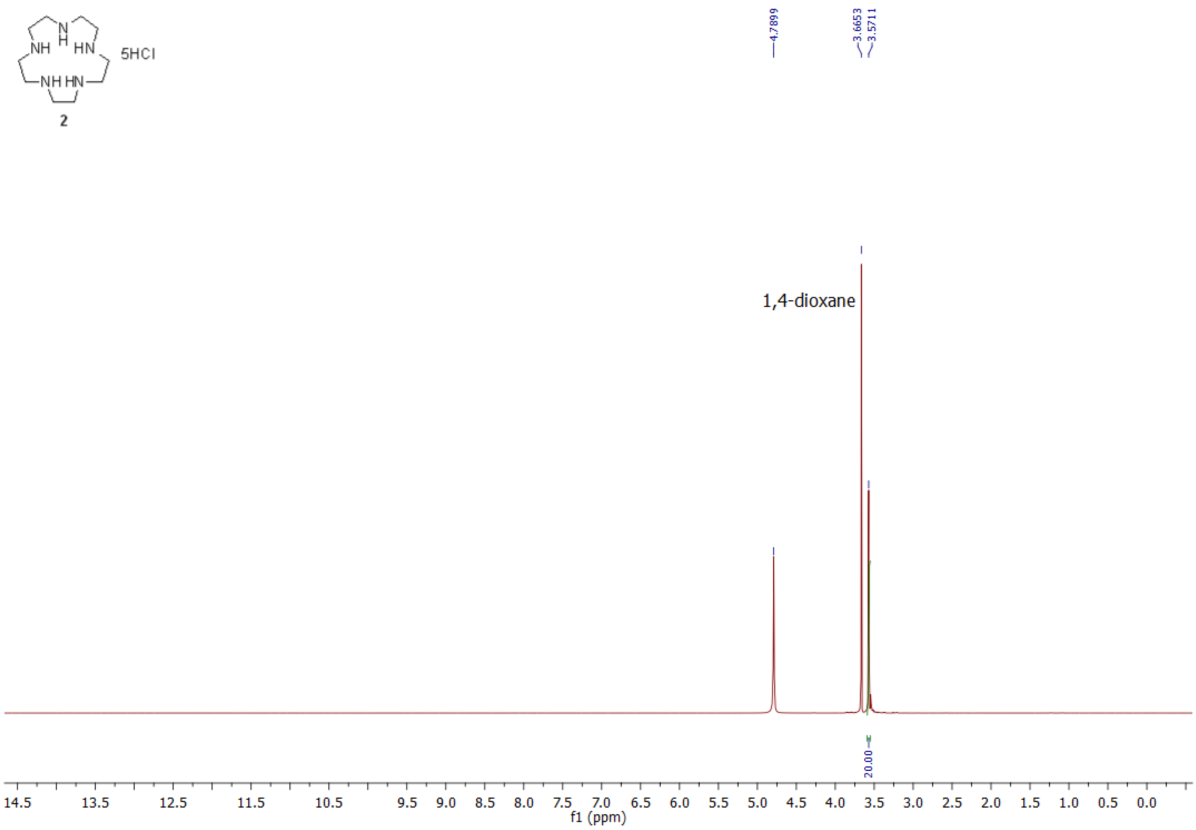

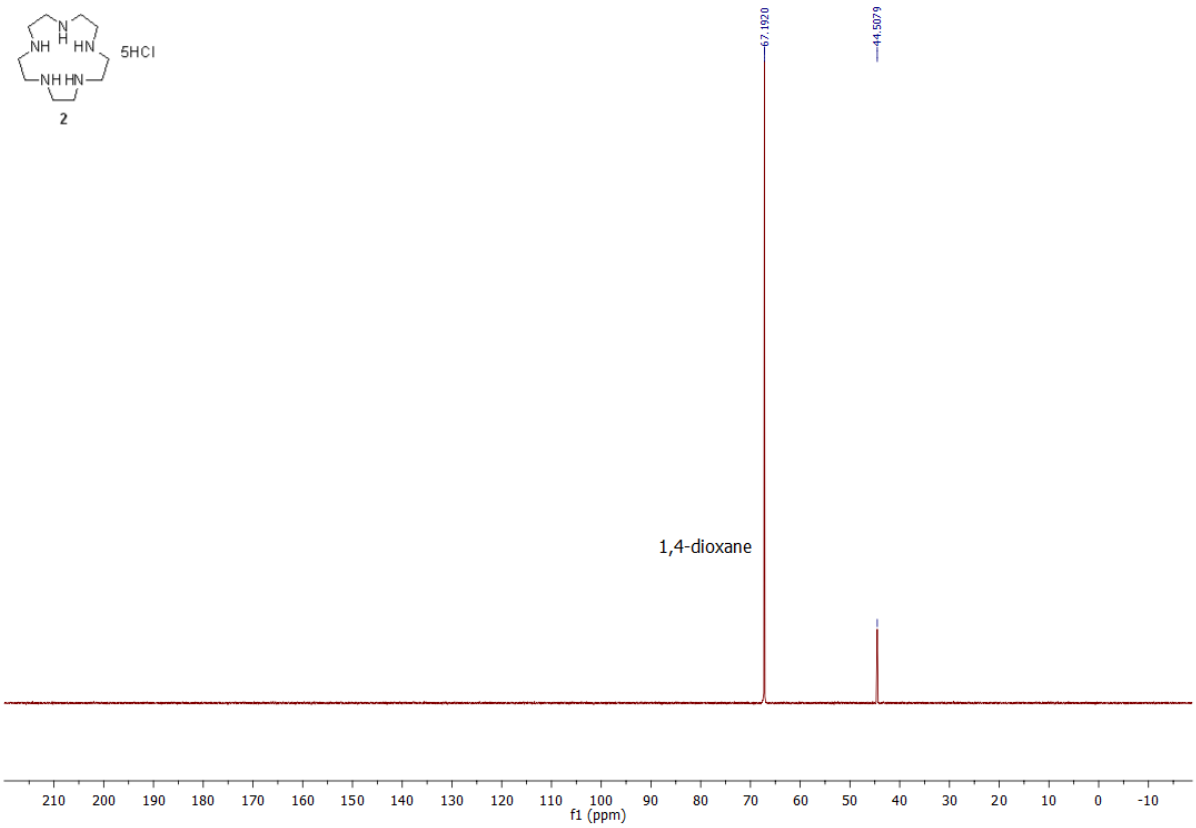

^1H (top) and ^{13}C (down) NMR spectra of compound **3** in $CDCl_3$

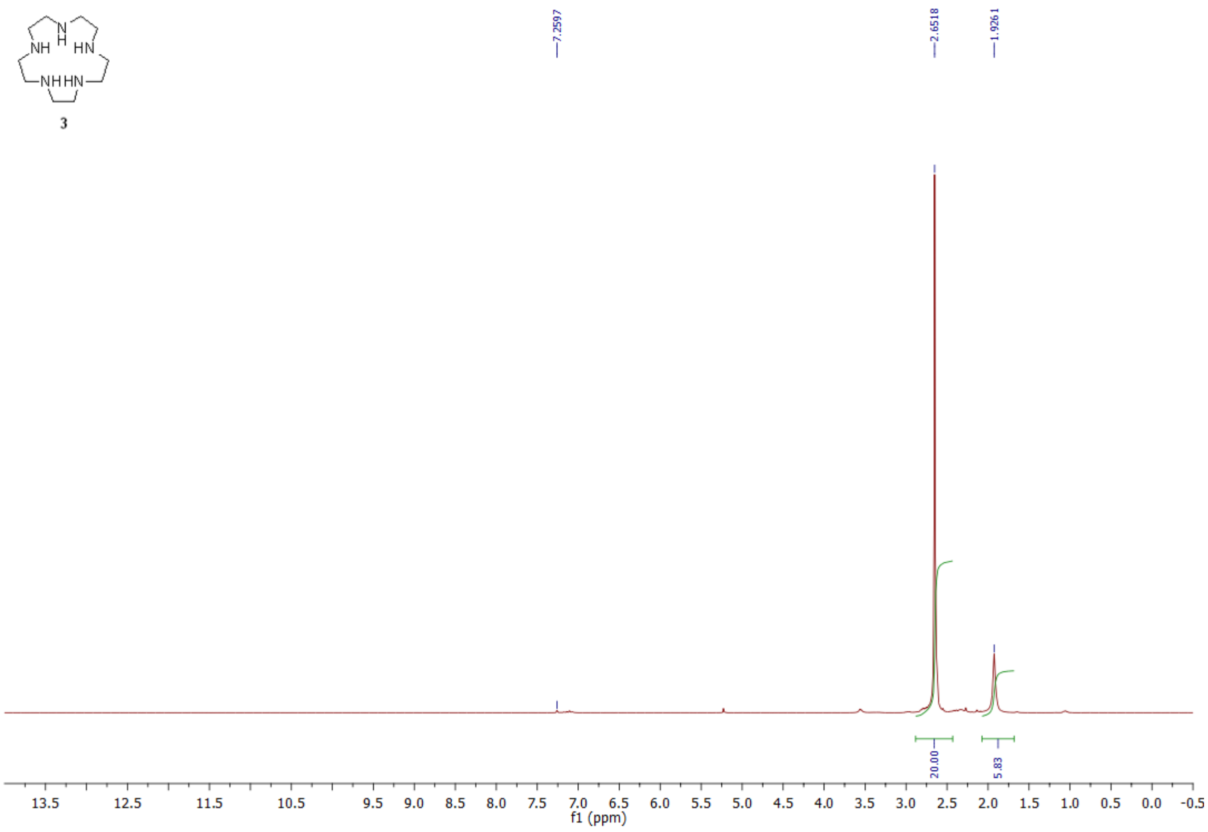

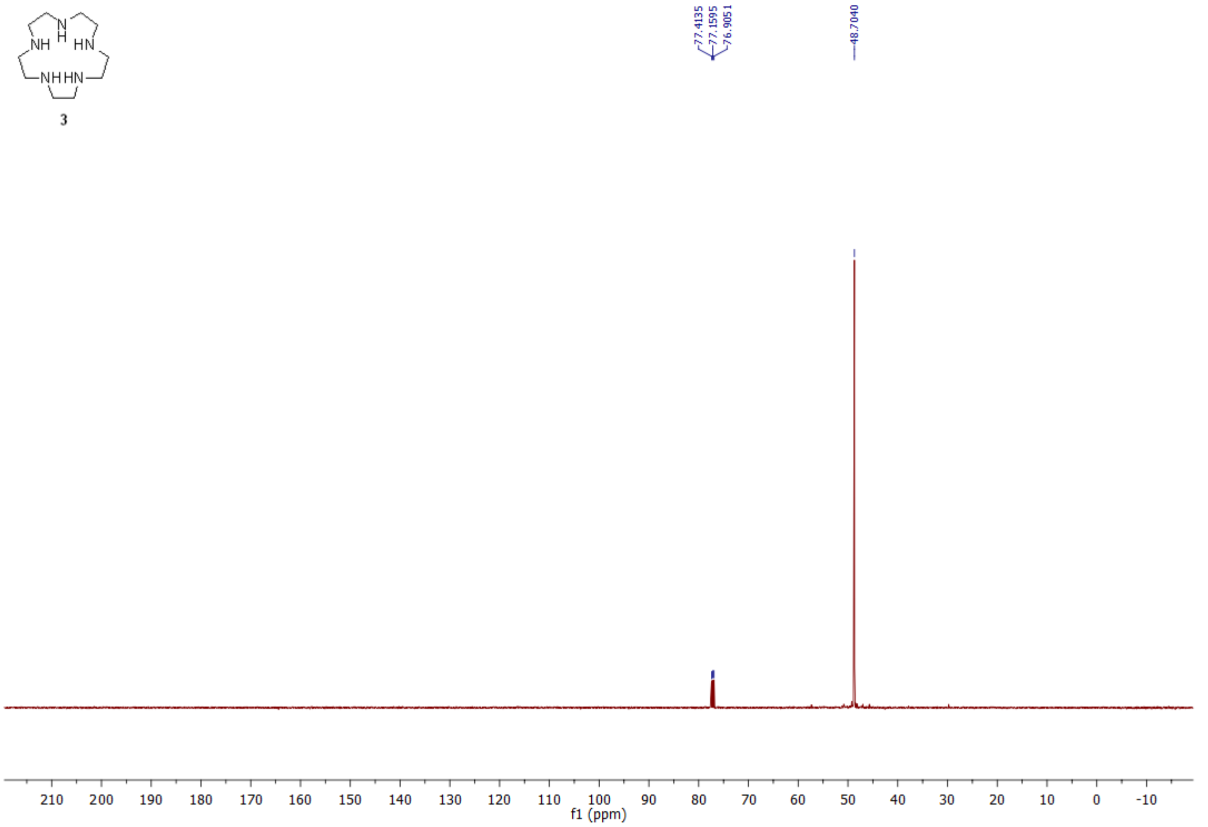

^{1}H (top) and ^{13}C (down) NMR spectra of compound **4** in $CDCl_3$

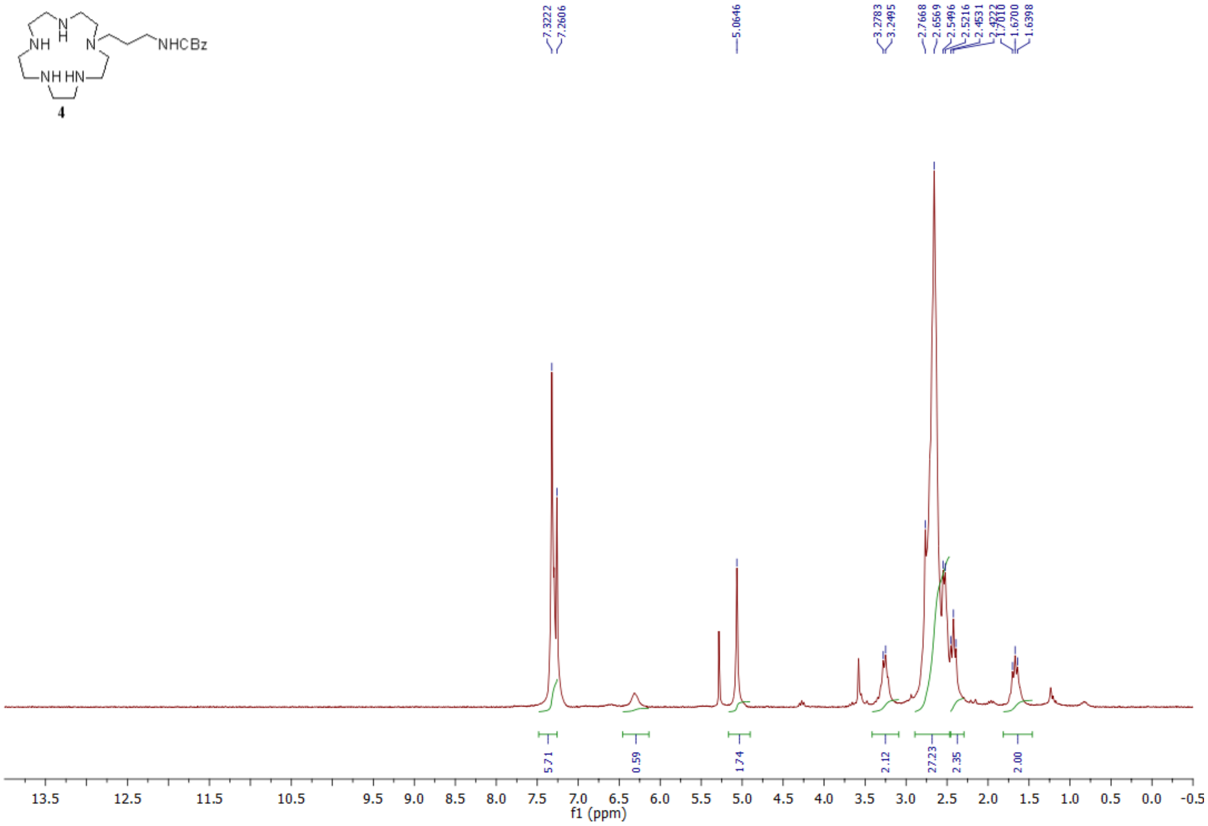

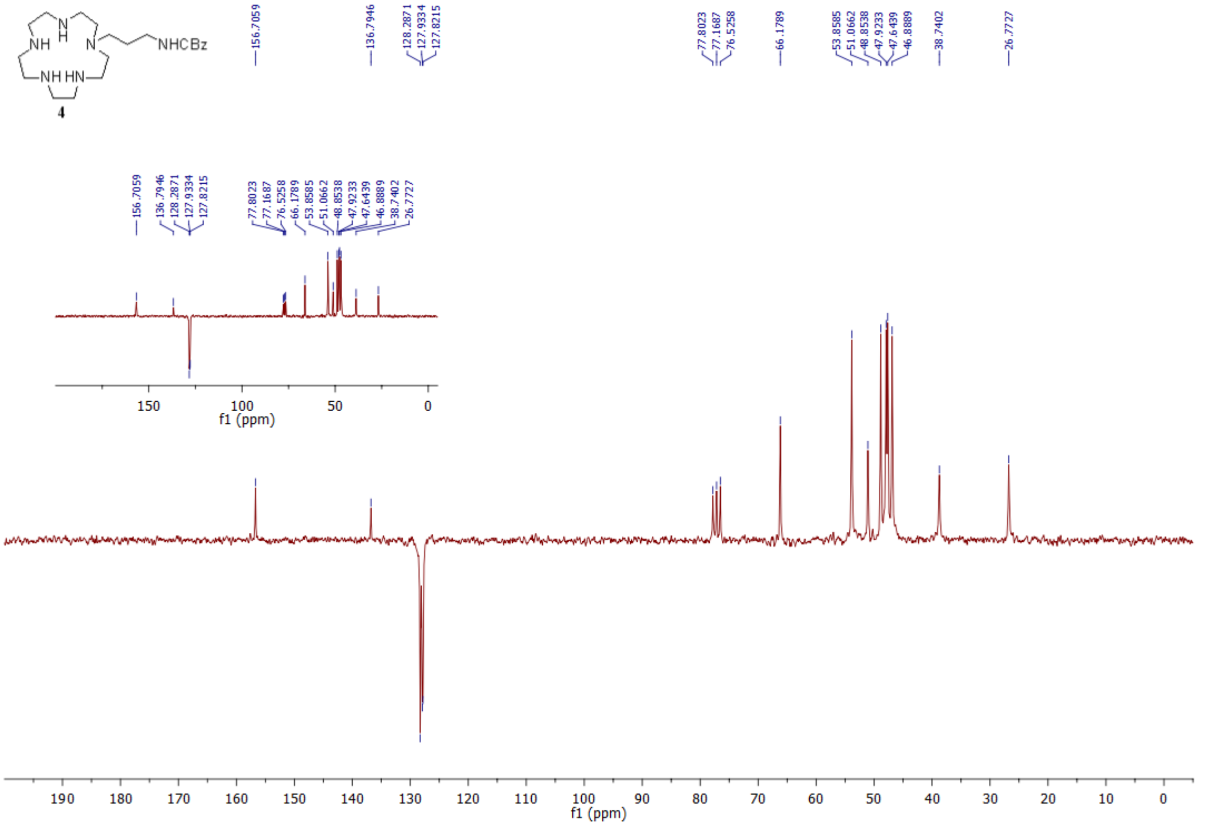

^{1}H (top) and ^{13}C (down) NMR spectra of compound **5** in $CDCl_3$

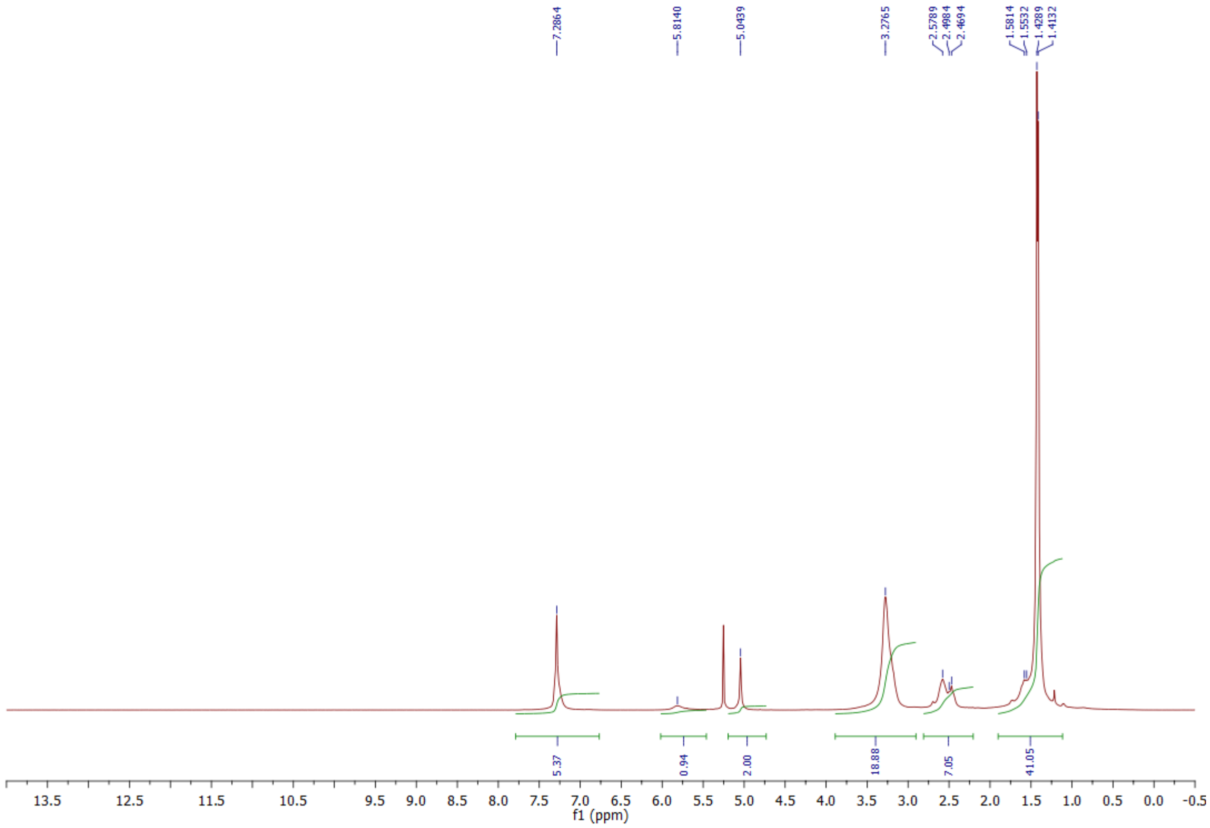

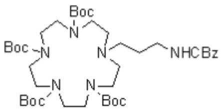

5

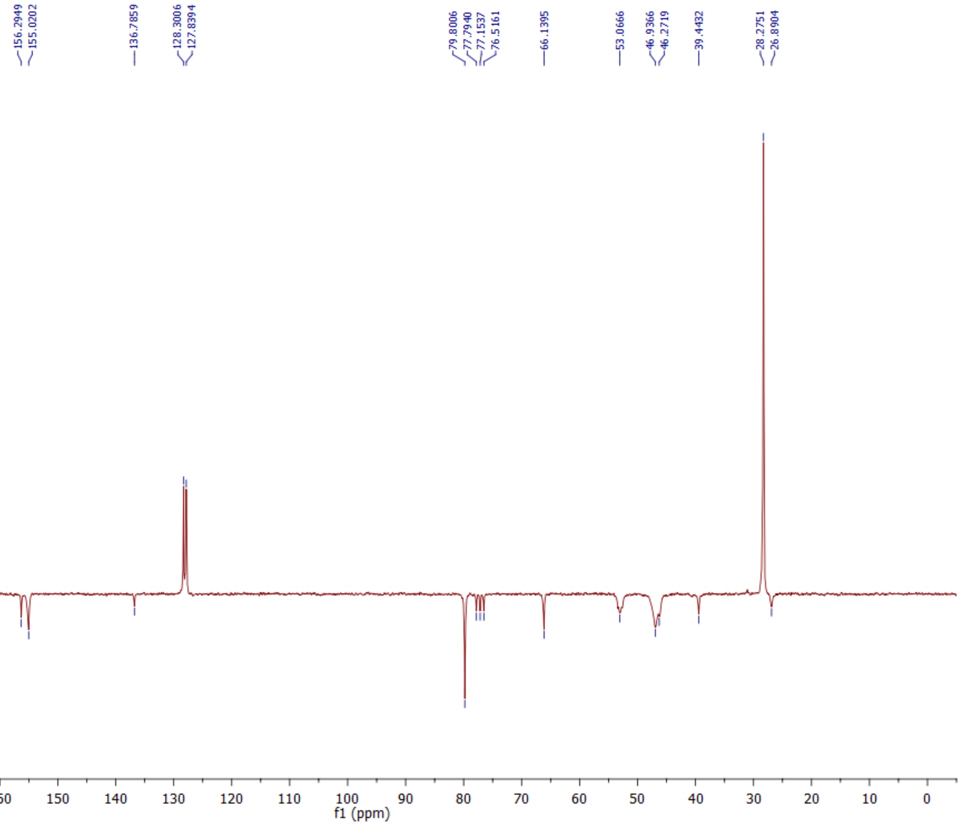

^{1}H (top) and ^{13}C (down) NMR spectra of compound **6** in $CDCl_3$

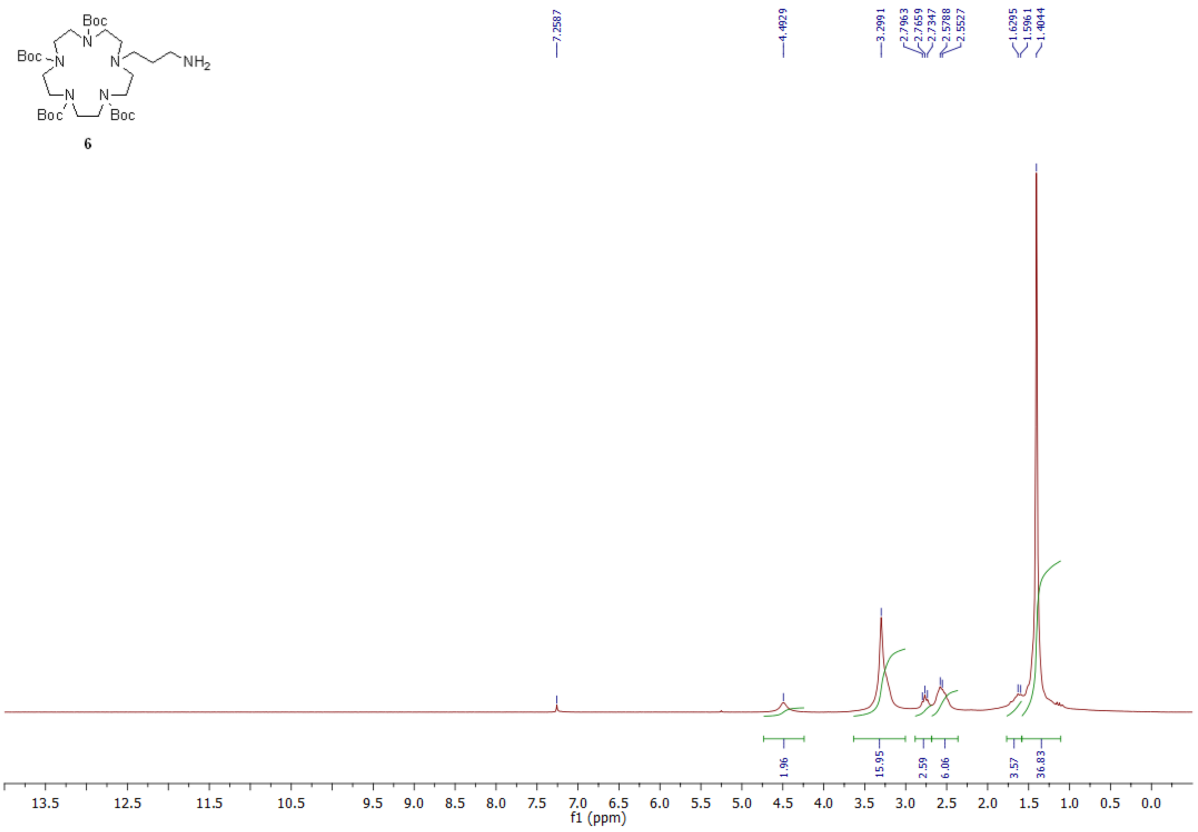

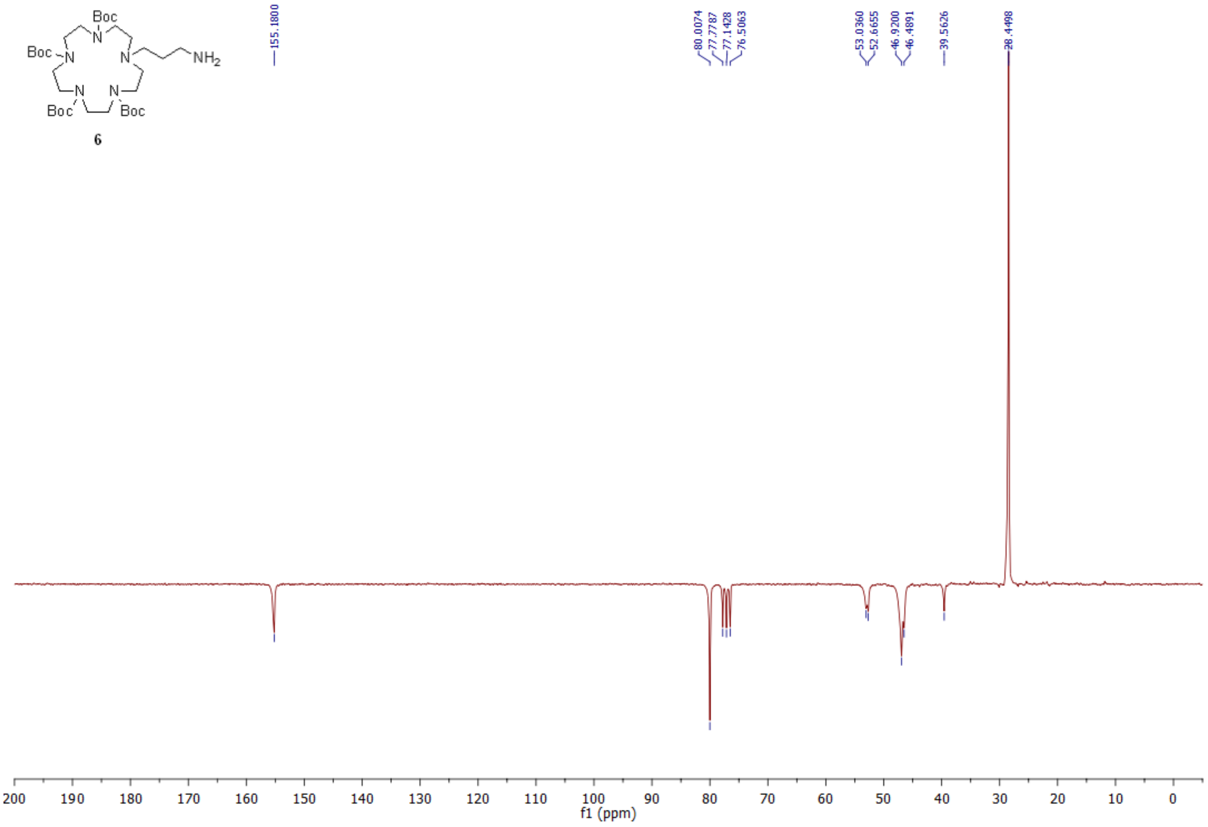

^{1}H (top) and ^{13}C (down) NMR spectra of compound **7** in $CDCl_3$

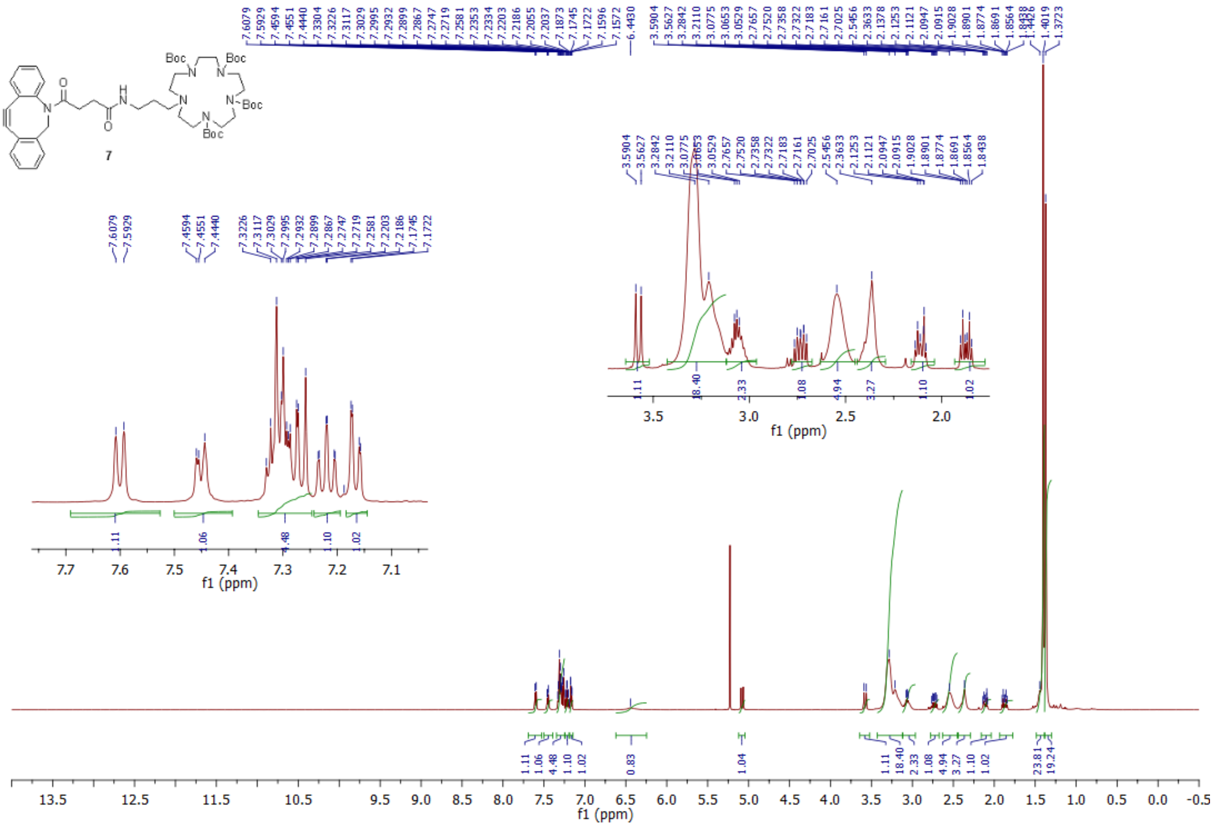

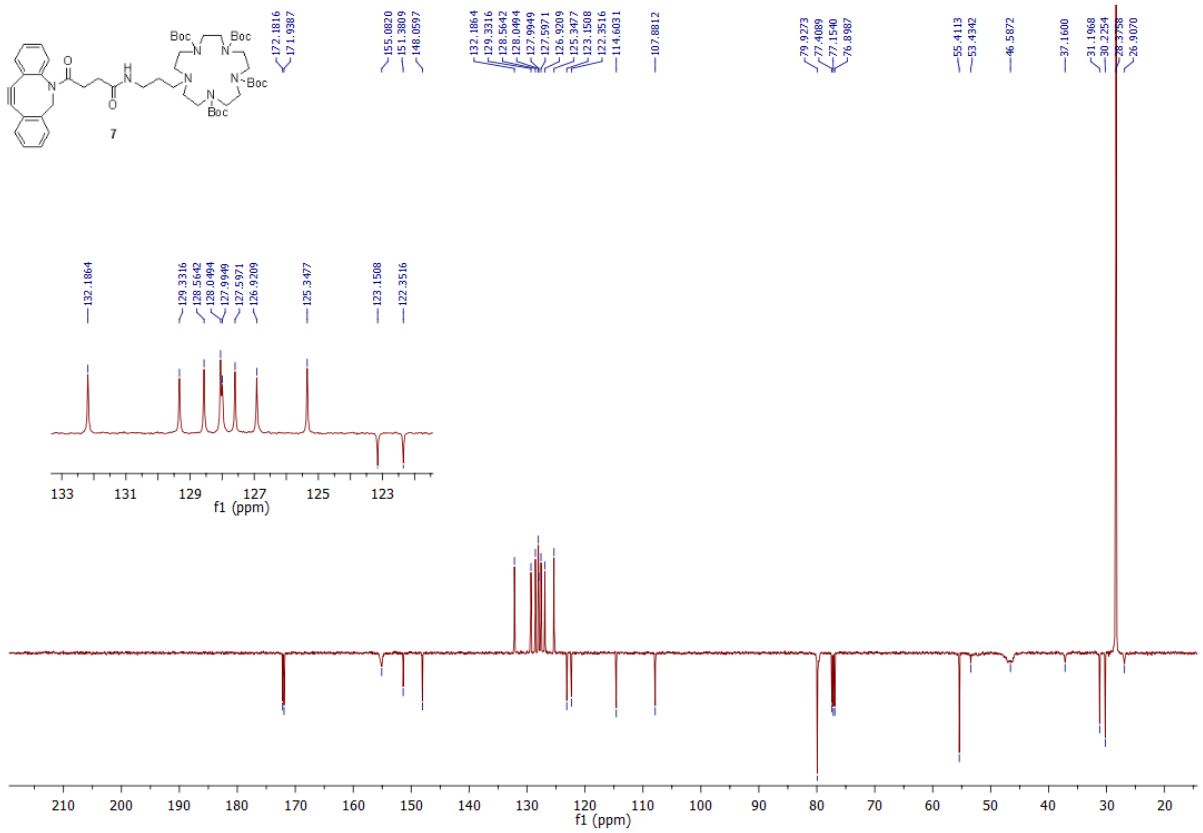

^{1}H (top) and ^{13}C (down) NMR spectra of compound **8** (**15-5-ADIBO**) in CD_3OD

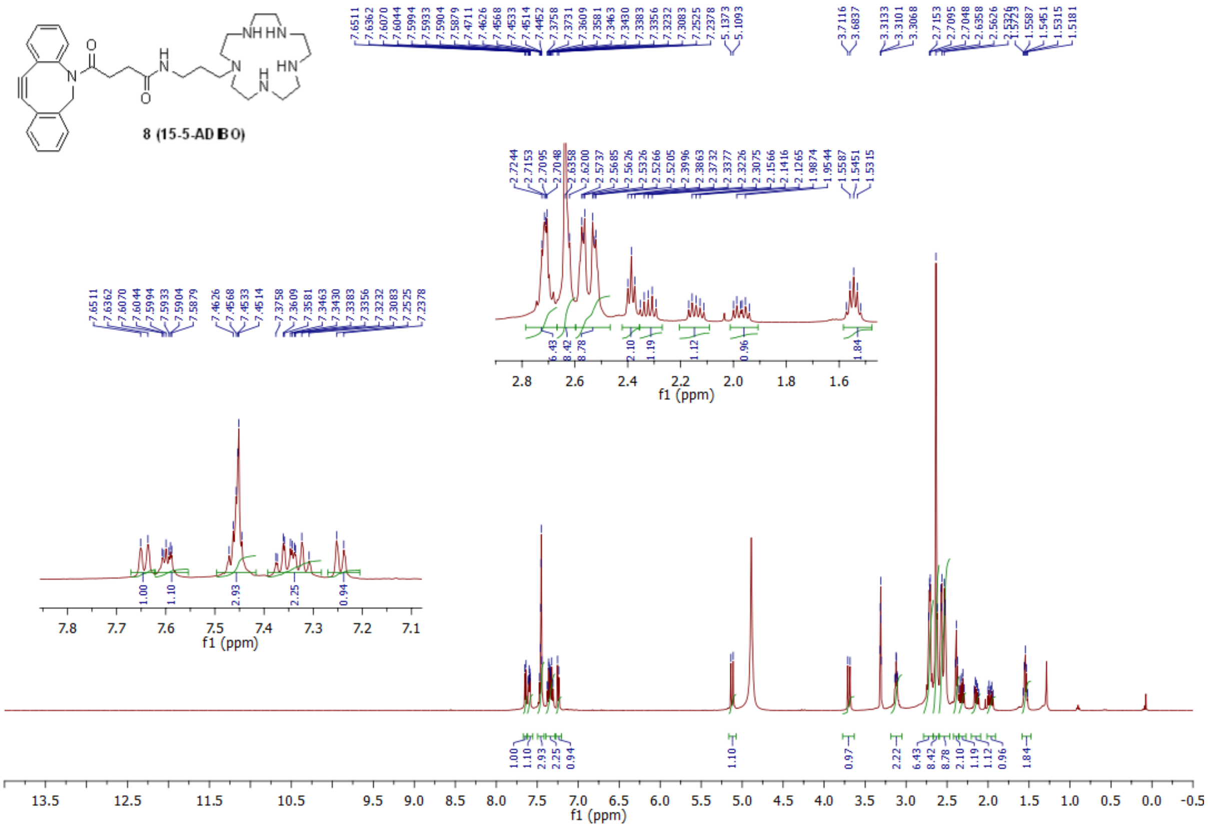

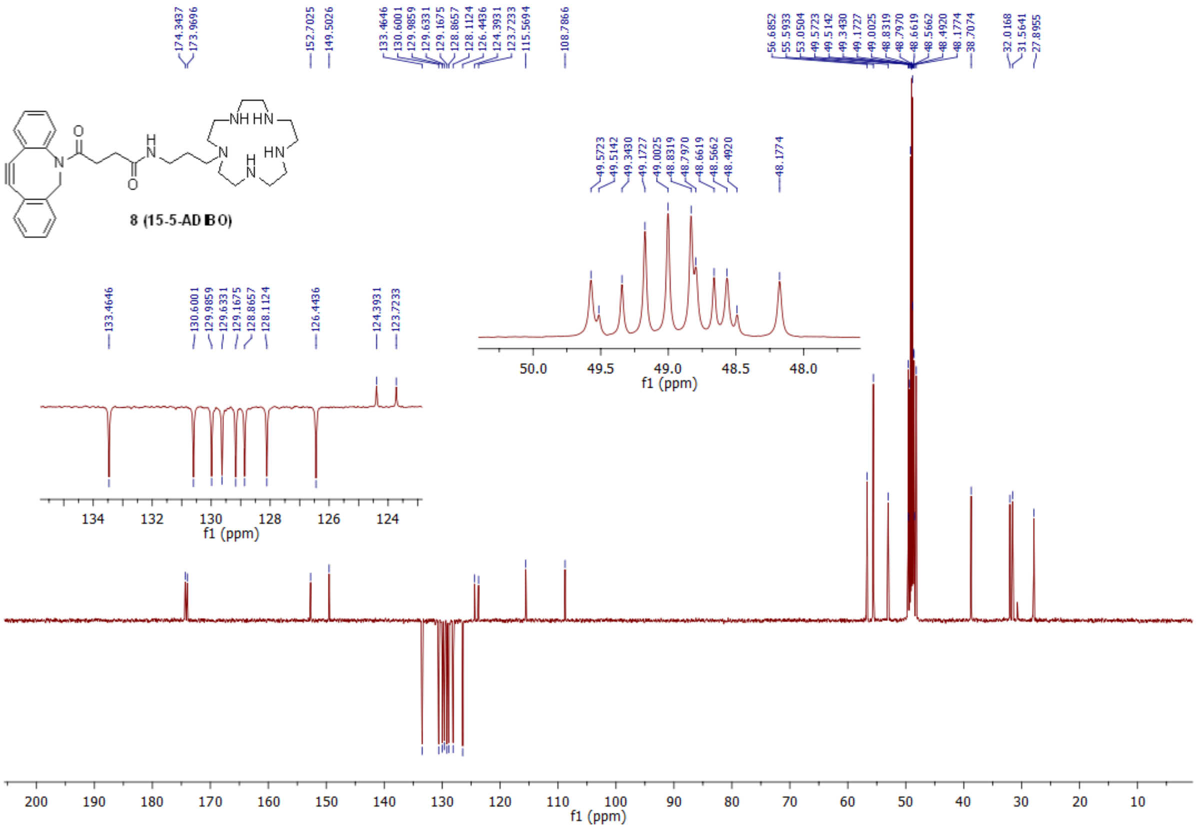

^{1}H (top) and ^{13}C (down) NMR spectra of compound **9** in $CDCl_3$ and CD_3CN, respectively

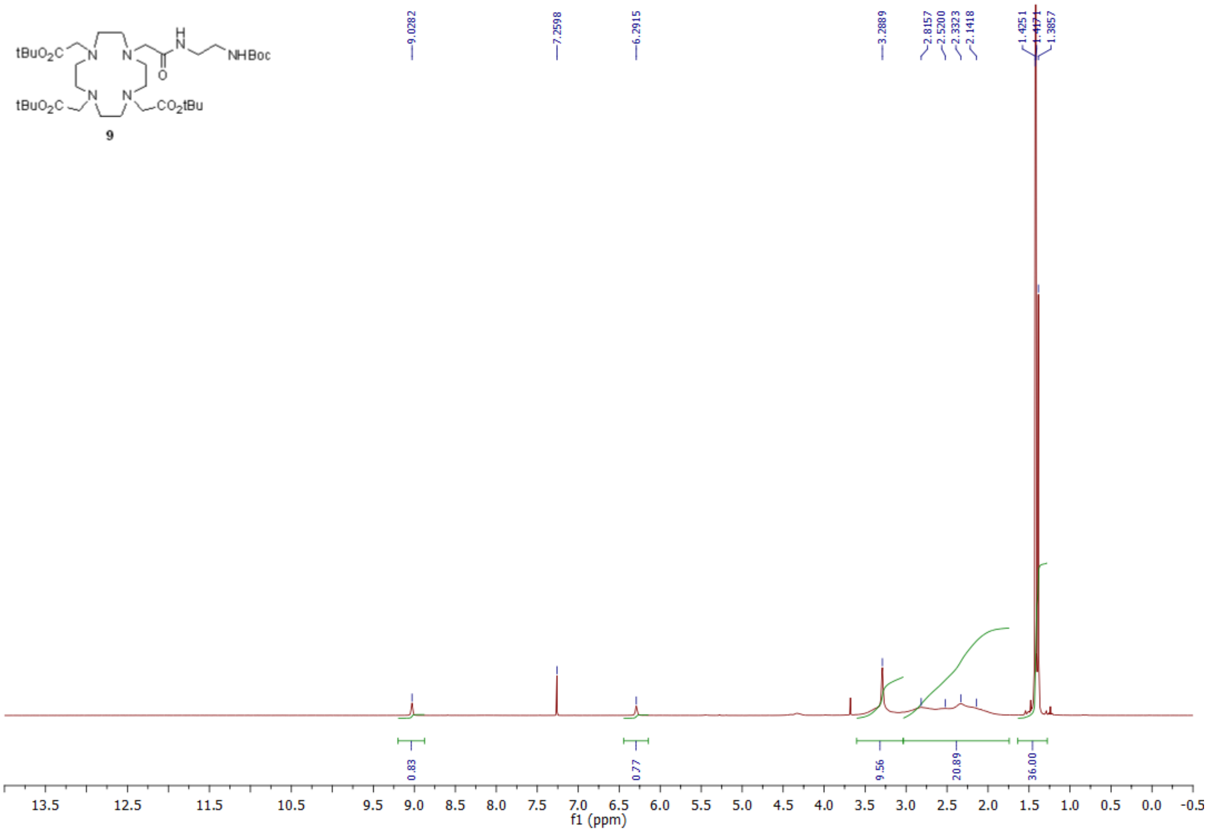

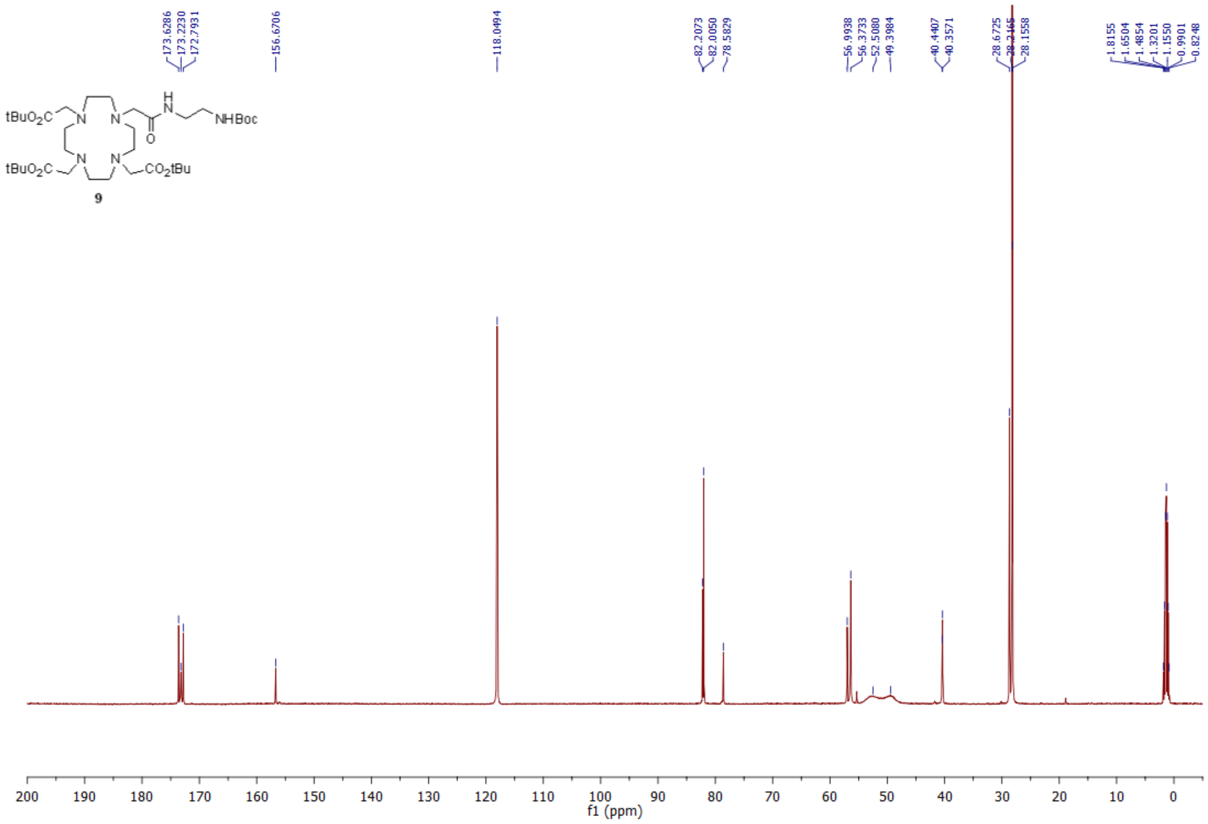

^{1}H (top) and ^{13}C (down) NMR spectra of compound **11** (**DOTA-ADIBO**) in CD_3OD

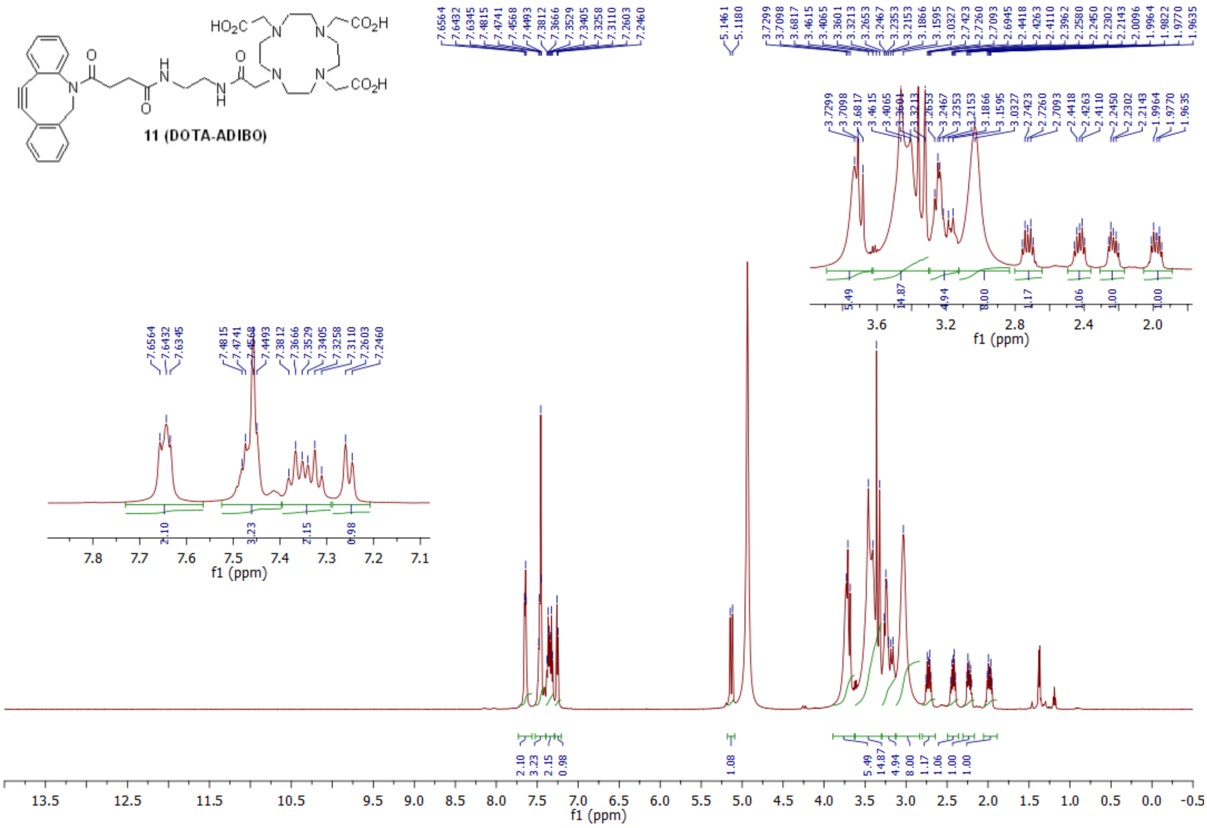

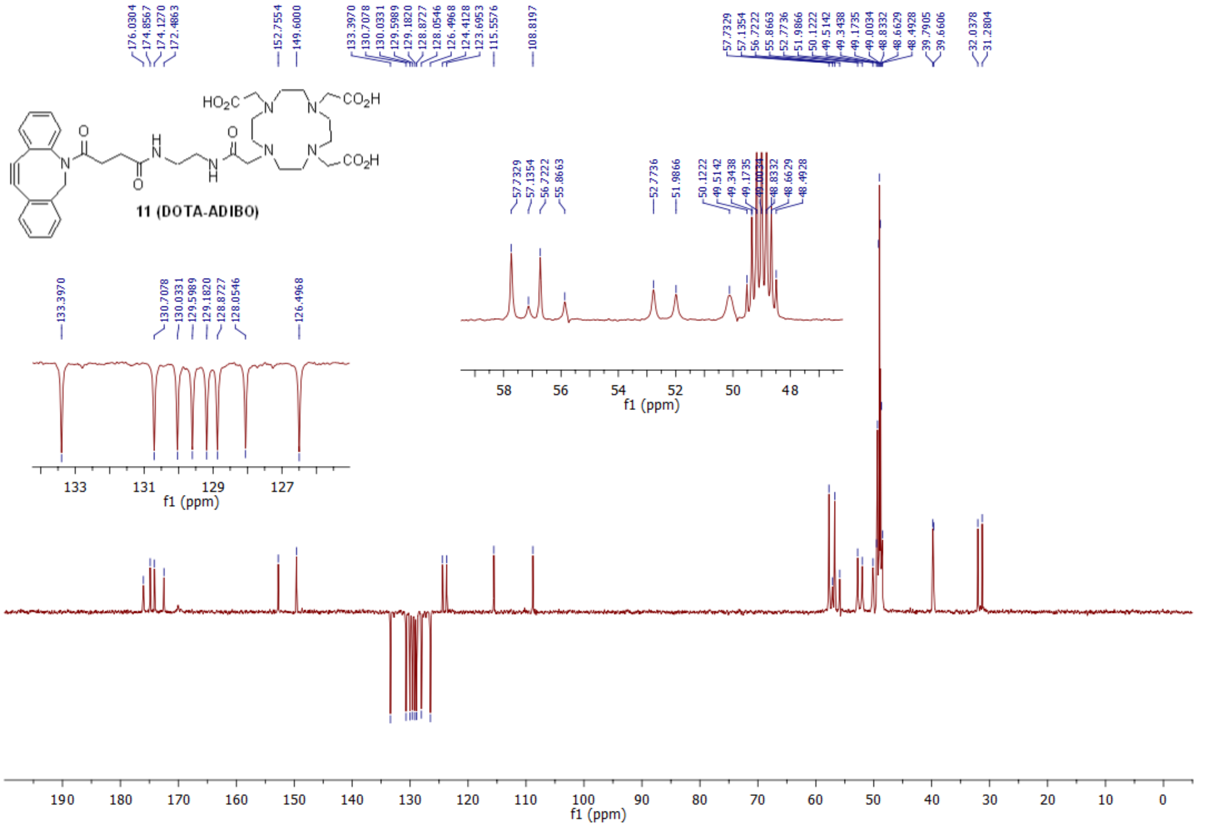

^{1}H (top) and ^{13}C (down) NMR spectra of compound **12** in $CDCl_3$

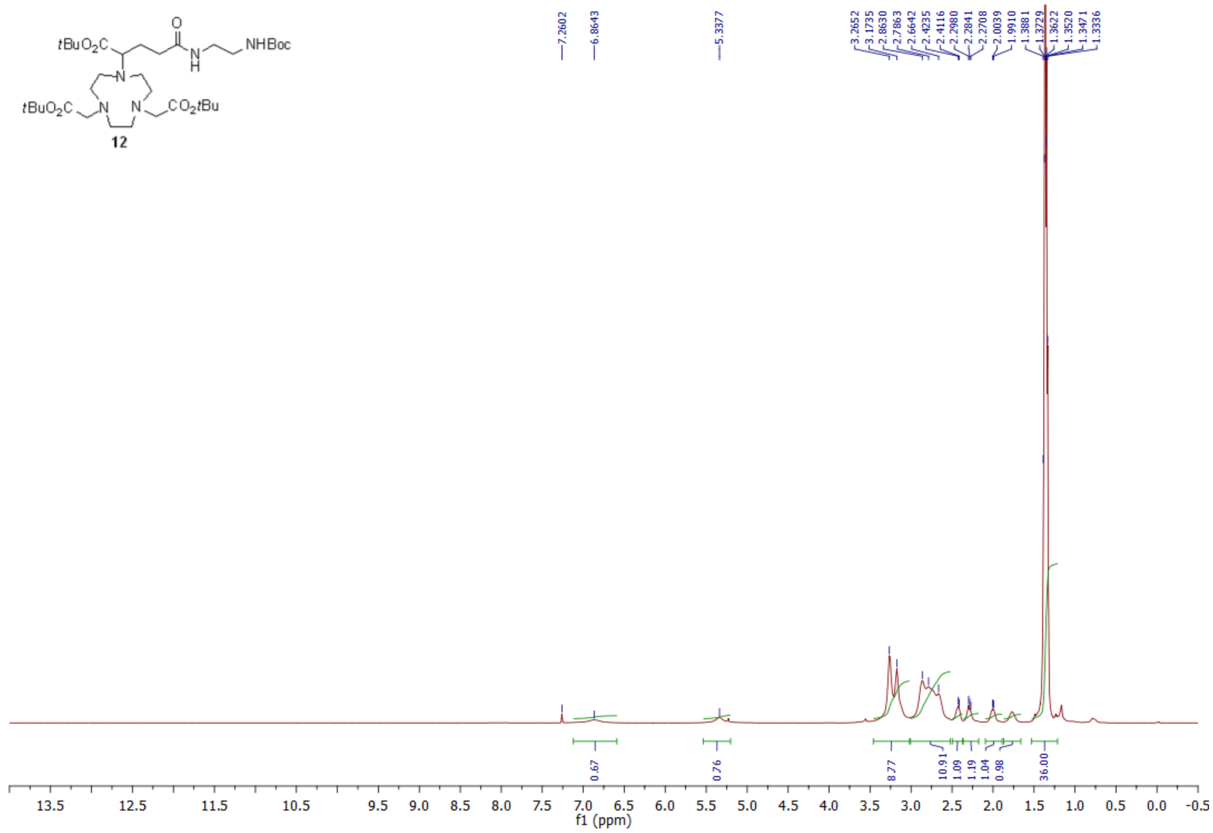

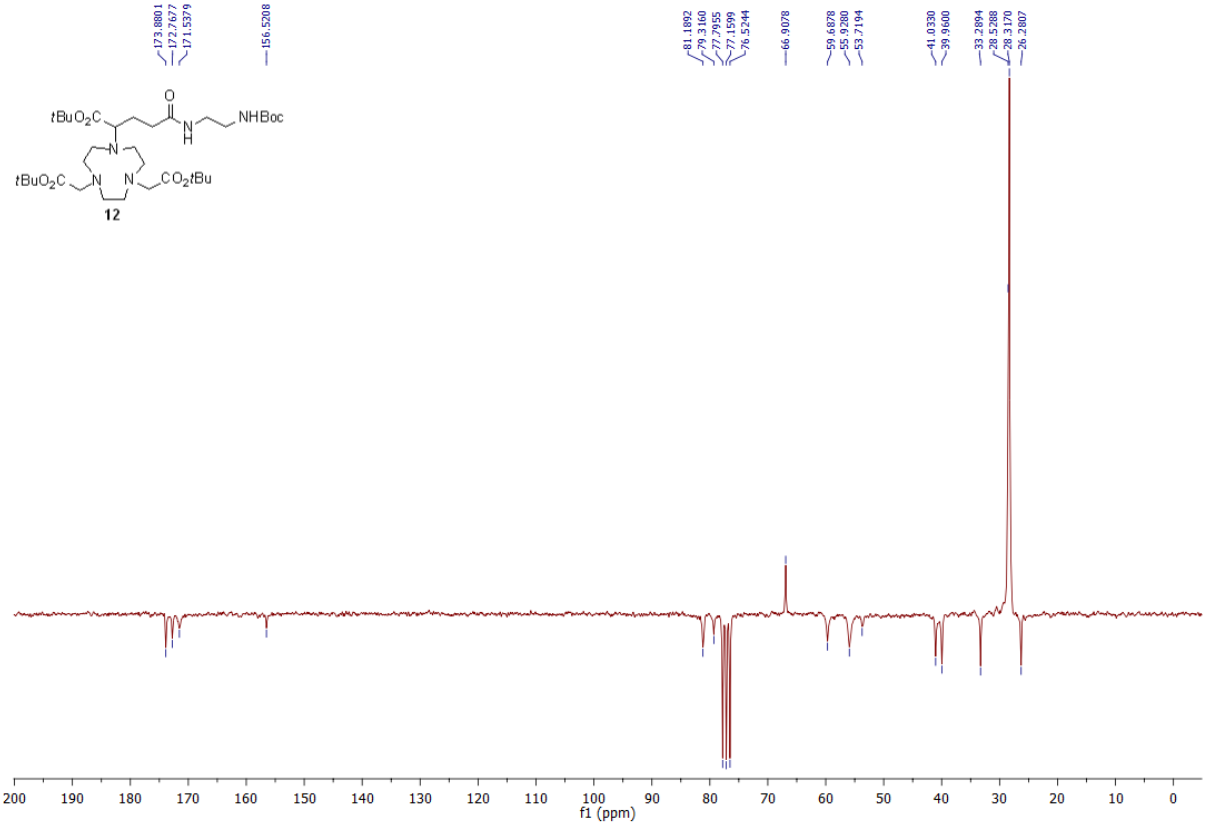

^{1}H (top) and ^{13}C (down) NMR spectra of compound **14** in CD_3OD

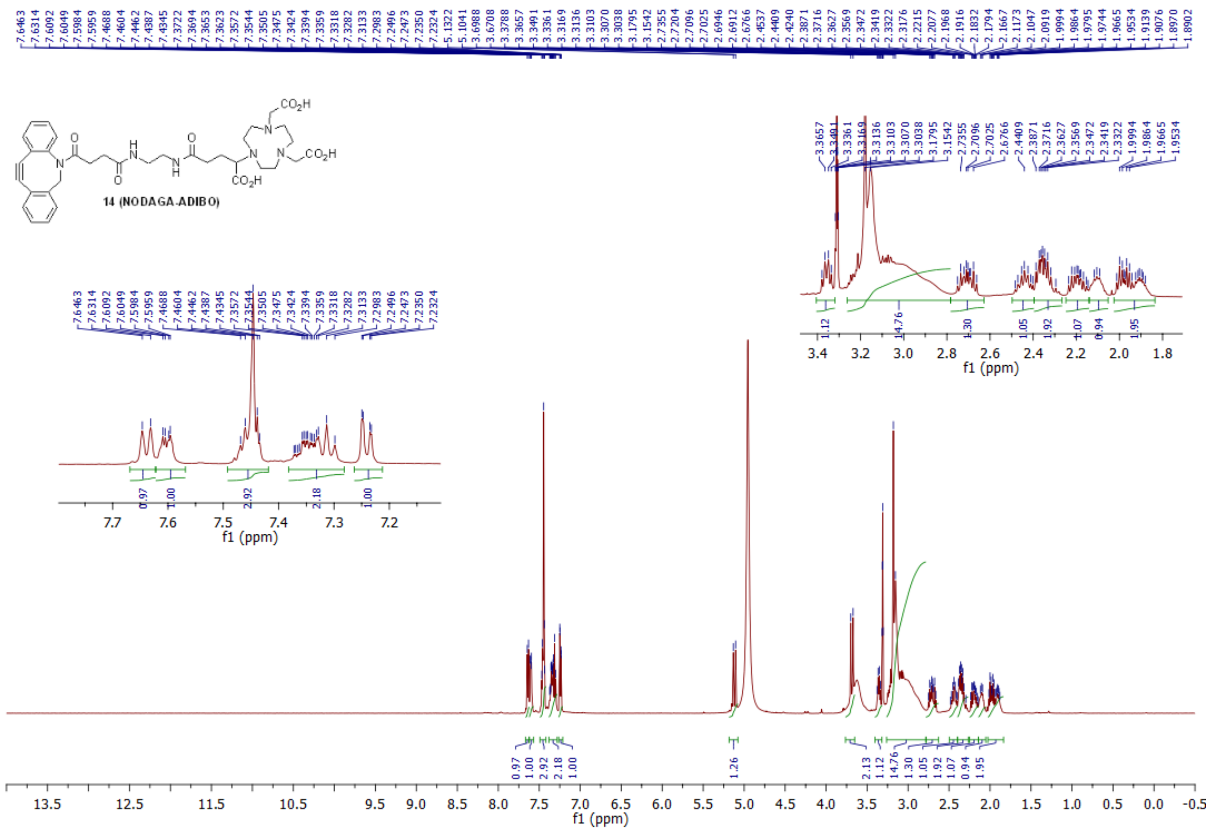

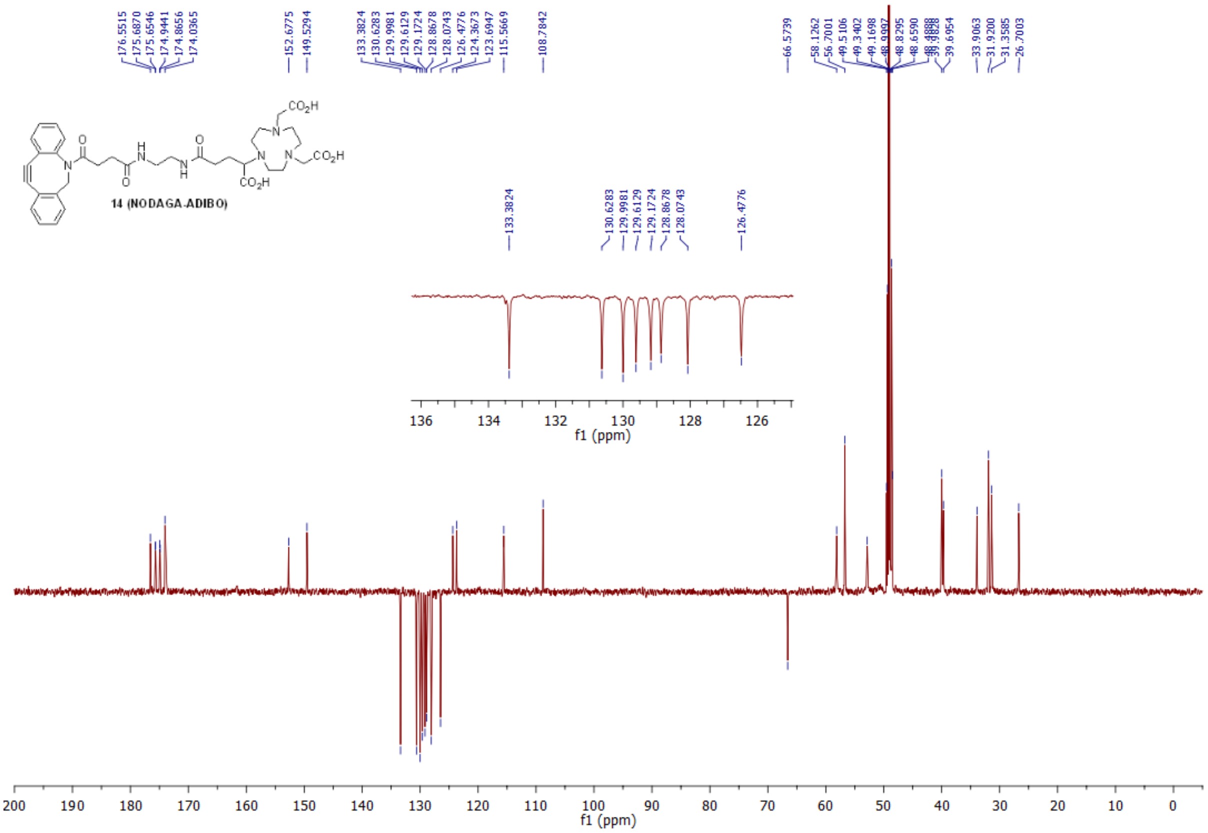

5. References

[1] Armarego, W.L.F.; Chai, C.L.L. Purification of Laboratory Chemicals. 6th ed. Oxford: Elsevier; **2009**.

[2] Chadwick, R.C.; Van Gyzen, S.; Adronov, A. Scalable synthesis of strained cyclooctyne derivatives. *Synthesis* **2014**, *46*, 669-677. https://doi.org/DOI:10.1055/S-0033-1340509.

[3] McNelles, S.A.; Adronov, A. Rapid synthesis of functionalized high-generation polyester dendrimers via strain-promoted alkyne-azide cycloaddition. *Macromolecules* **2017**, *50*, 7993-8001. https://doi.org/10.1021/acs.macromol.7b01765.

[4] Campbell-Verduyn, L.S.; Mirfeizi, L.; Schoonen, A.K.; Dierckx, R.A.; Elsinga, P.H.; Feringa, B.L. Strain promoted copper-free "click" chemisty for ^{18}F-radiolabeling of bombesin. *Angew. Chem. Int. Ed. Engl.* **2011**, *50*, 11117-11120. https://doi.org/10.1002/anie.201105547.

[5] Yale, H.L.; Sowinski, F.A. 5-(Substituted amino-lower alkylene)-5,6-dihydrodibenz[*b*,*f*]azocines. United States Patent 3514443. **1970**.

[6] von Wantoch Rekowski, M.; Kumar, V.; Zhou, Z.; Moschner, J.; Marazioti, A.; Bantzi, M.; Spyroulias, G.A.; van den Akker, F.; Giannis, A.; Papapetropoulos, A. Insights into soluble guanylyl cyclase activation derived from improved heme-mimetics. *J. Med. Chem.* **2013**, *56*, 8948-8952. https://doi.org/10.1021/jm400539d.

[7] Wei, W.H.; Tomohiro, T.; Kodaka, M.; Okuno, H. Selective synthesis and kinetic measurement of 1:1 and 2:2 cyclic compounds containing 1,4,7,10-tetraazacyclododecane and azobenzene units. *J. Org. Chem.* **2000**, *65*, 8979-8987. https://doi.org/10.1021/jo000926p.

[8] Moore, D.A. Selective trialkylation of cyclen with *tert*-butyl bromoacetate [1,4,7,10-tetraazacyclododecane-1,4,7-triacetic acid, tri-*tert*-butyl ester hydrobromide]. *Org Synth.* **2008**, *85*, 10-14. https://doi.org/10.15227/orgsyn.085.0010.

[9] Jagadish, B.; Brickert-Albrecht, G.L.; Nichol, G.S.; Mash, E.A.; Raghunand, N. On the synthesis of 1,4,7-tris(*tert*-butoxycarbonylmethyl)-1,4,7,10-tetraazacyclododecane. *Tetrahedron Lett.* **2011**, *52*, 2058-2061. https://doi.org/10.1016/j.tetlet.2010.10.074.

[10] Strauch, R.C.; Mastarone, D.J.; Sukerkar, P.A.; Song, Y.; Ipsaro, J.J.; Meade, T.J. Reporter protein-targeted probes for magnetic resonance imaging. *J. Am. Chem. Soc.* **2011**, *133*, 16346-16349. https://doi.org/10.1021/ja206134b.

[11] Kohl, S.W.; Kuse, K.; Hummert, M.; Schumann, H.; Mügge, C.; Janek, K.; Weisshoff, H. New synthetic routes for 1-benzyl-1,4,7,10-tetraazacyclododecane and 1,4,7,10-tetraazacyclododecane-1-acetic acid ethyl ester, important starting materials for metal-coded DOTA-based affinity tags. *Z. Naturforsch. B* **2007**, *62b*, 397-406. https://doi.org/10.1515/znb-2007-0313.

[12] Grate, J.W.; Mo, K.F.; Daily, M.D. Triazine-based sequence-defined polymers with side-chain diversity and backbone-backbone interaction motifs. *Angew. Chem. Int. Ed. Engl.* **2016**, *55*, 3925-3930. https://doi.org/10.1002/anie.201509864.

[13] Montagner, D.; Gandin, V.; Marzano, C.; Erxleben, A. Phosphate diester cleavage, DNA interaction and cytotoxic activity of a bimetallic bis(1,4,7-triazacyclononane) zinc complex. *Eur. J. Inorg. Chem.* **2014**, *2014*, 4084-4092. https://doi.org/10.1002/ejic.201402319.

[14] Searle, G.H.; Geue, R.J. Improved Richman-Atkins syntheses of cyclic polyamines particularly 1,4,7-triazacyclononane (tacn) and 1,4,7-triazacyclodecane (tacd), with the aid of cation-exchange in purification and isolation. *Aust. J. Chem.* **1984**, *37*, 959-970. https://doi.org/10.1071/CH9840959.

[15] Zhang, J.; Voegtle, F. Synthesis of *N*,*N'*,*N''*-trisubstituted 1,4,7-triazacyclononane. *Chin. J. Org. Chem.* **1987**, 273-277.

[16] Stavila, V.; Allali, M.; Canaple ; L.; Stortz, Y.; Franc, C.; Maurin, P.; Beuf, O.; Dufay, O.; Samarut, J.; Janier, M.; Hasserodt, J. Significant relaxivity gap between a low-spin and a high-spin iron(II) complex of structural similarity: an attractive off-on system for the potential design of responsive MRI probes. *New J. Chem.* **2008**, *32*, 428-435. https://doi.org/10.1039/B715254J.

[17] Yoshida, M.; Narita, M.; Hara, S. Asymmetric Michael addition of malonates to enones catalyzed by a primary β-amino acid and its lithium salt. *J. Org. Chem.* **2011**, 76, 8513-857. https://doi.org/10.1021/jo201429w.

[18] Albert, R.; Danklmaier. J.; Hönig, H.; Kandolf, H. A simple and convenient synthesis of β-aspartates and γ-glutamates. *Synthesis* **1987**, *1987*, 635-637. https://doi.org/10.1055/s-1987-28030.

[19] Lamarque, L.; Montgomery, C.; Parker, D. Pyridyl-aza(thio)xanthone sensitizer comprizing lanthanide(III) ion complexing compounds, their luminescent lanthanide (III) ion complexes and use thereof as fluorescent labels. WO2010084090. **2010**.

[20] Shetty, D.; Choi, S.Y.; Jeong. J.M.; Lee, J.Y.; Hoigebazar, L.; Lee, Y.S.; Lee, D.S.; Chung, J.K.; Lee, M.C.; Chung, Y.K. Stable aluminium fluoride chelates with triazacyclononane derivatives proved by X-ray crystallography and ^{18}F-labeling study. *Chem. Commun.* **2011**, *47*, 9732-9734. https://doi.org/10.1039/c1cc13151f.

[21] Denoël, T.; Pedrelli, L.; Pantaleo, G.; Prio, J.O. A robust method for assaying the immunoreactive fraction in nonequilibrium systems. *Pharmaceuticals* **2019**, *12*, 177. https://doi.org/10.3390/ph12040177.
